# Supplementary material for: Association Analysis in Young and Middle-Aged Mothers—Relation between Expression of Cardiovascular Disease Associated MicroRNAs and Abnormal Clinical Findings
Source: J Pers Med. 2021 Jan 11;11(1):39. doi: 10.3390/jpm11010039 (PMC7826744; doi:10.3390/jpm11010039)
Supplement: Supplementary file 1 [file jpm-11-00039-s001.zip › Supplementary Material/Supplementary Figure S1.docx]

**
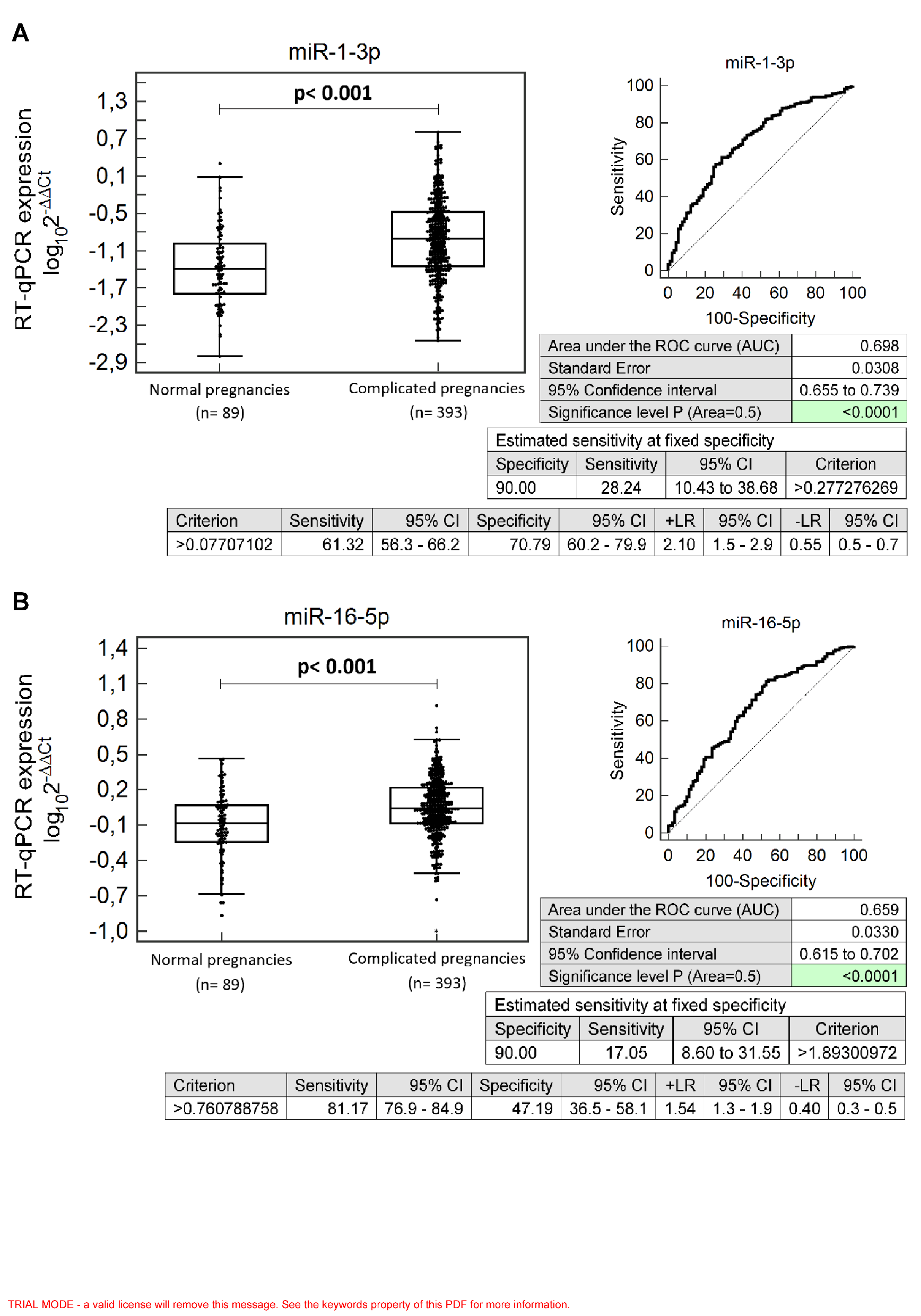
Supplementary Figure S1.**

**
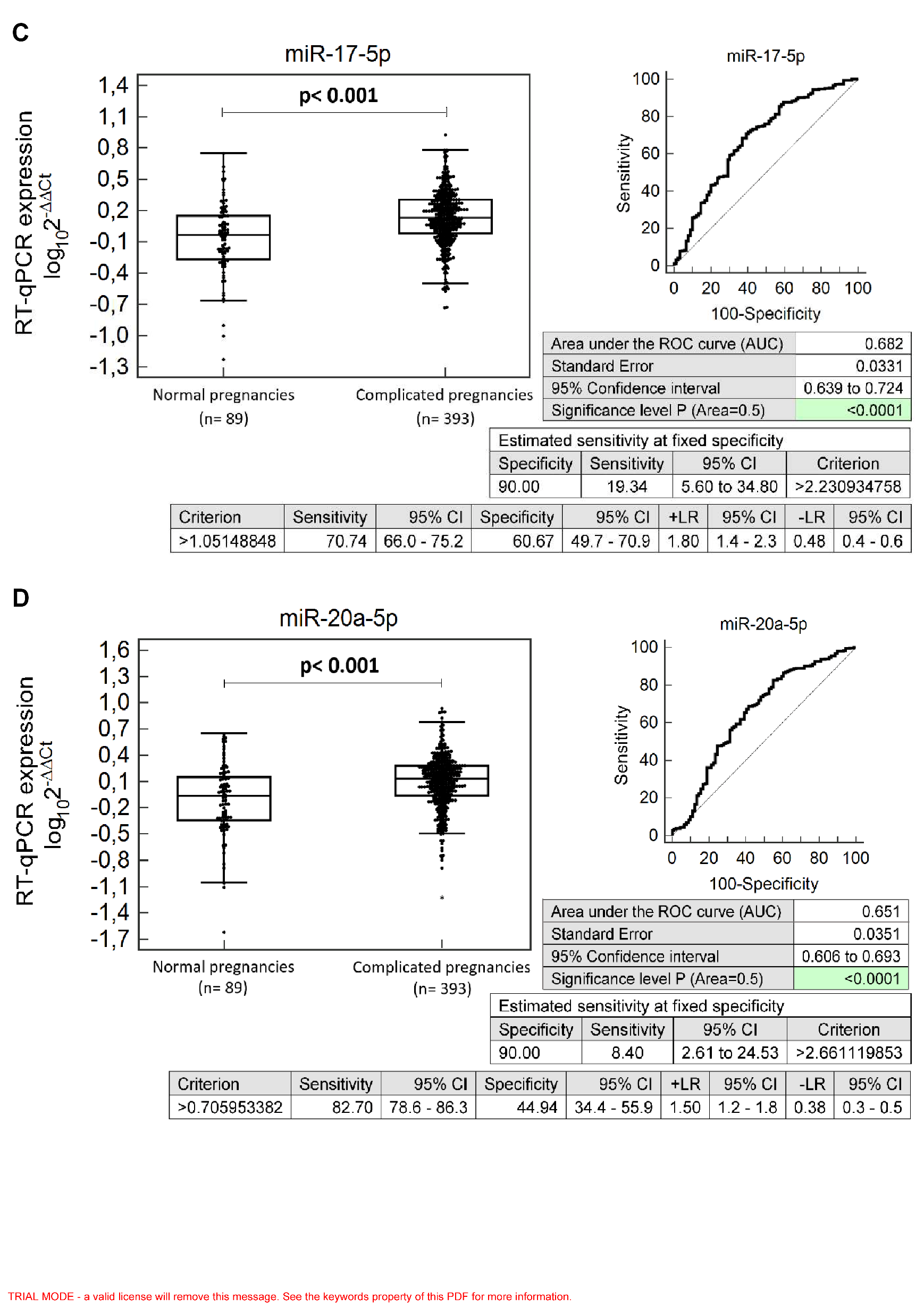
**


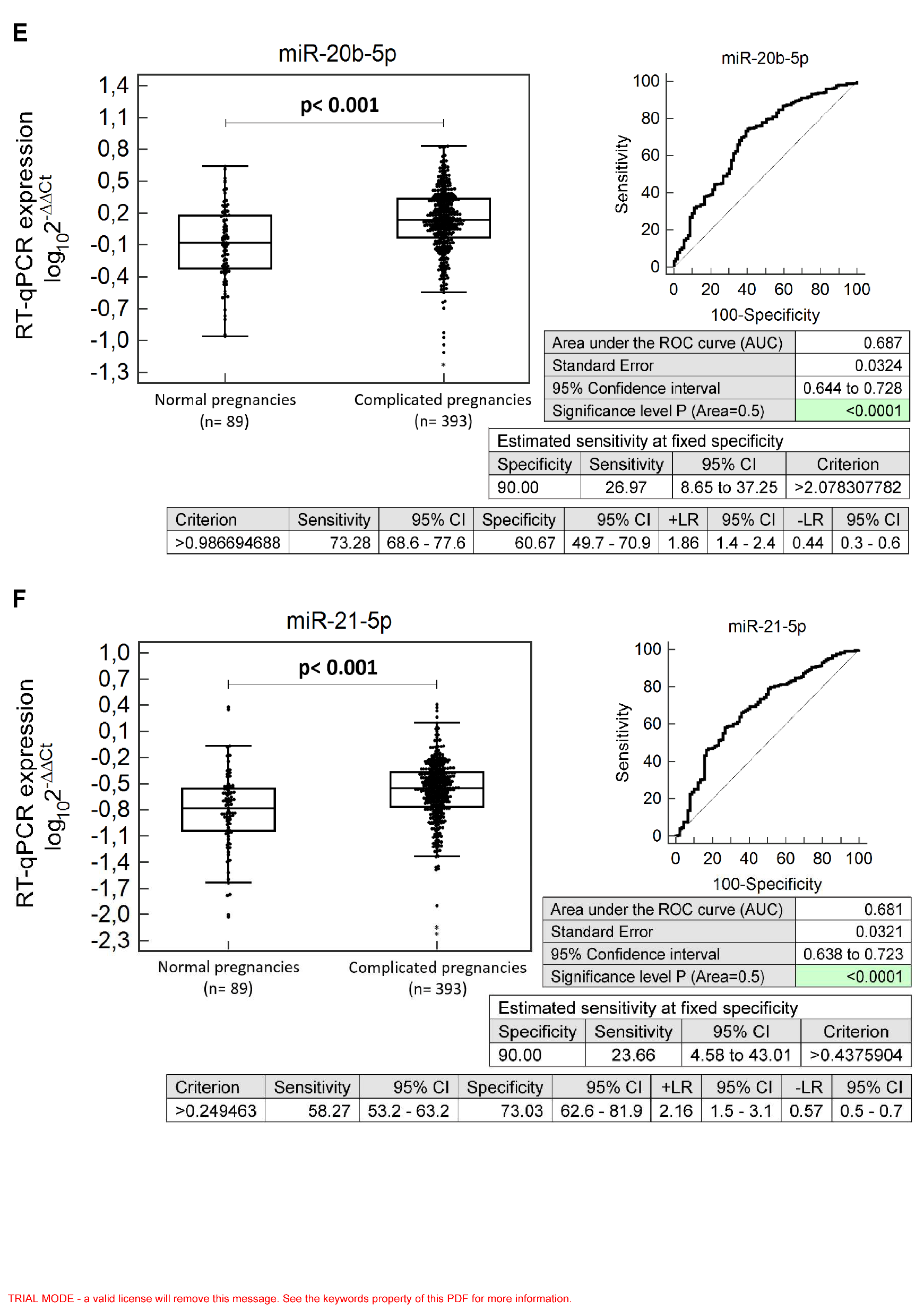


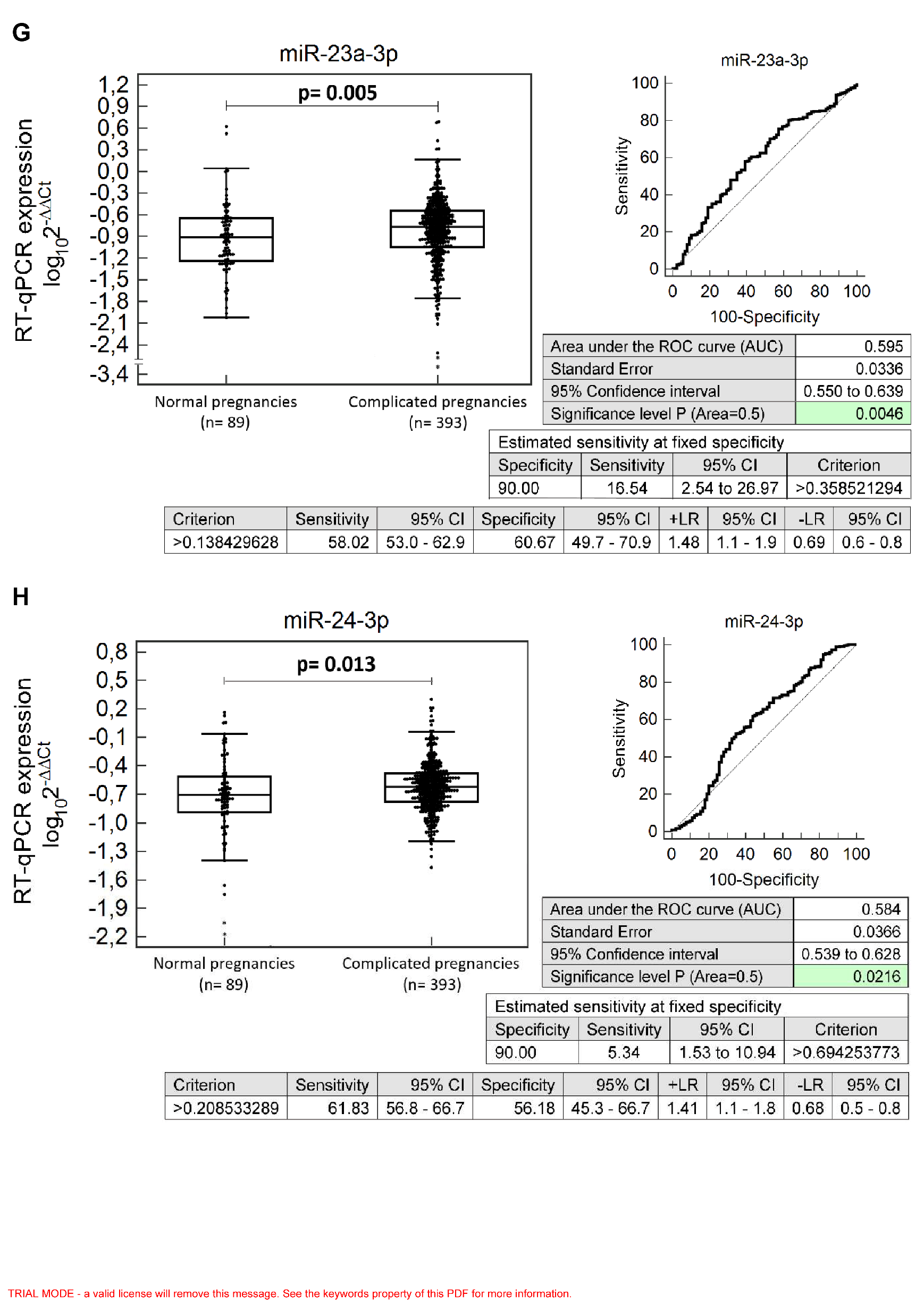


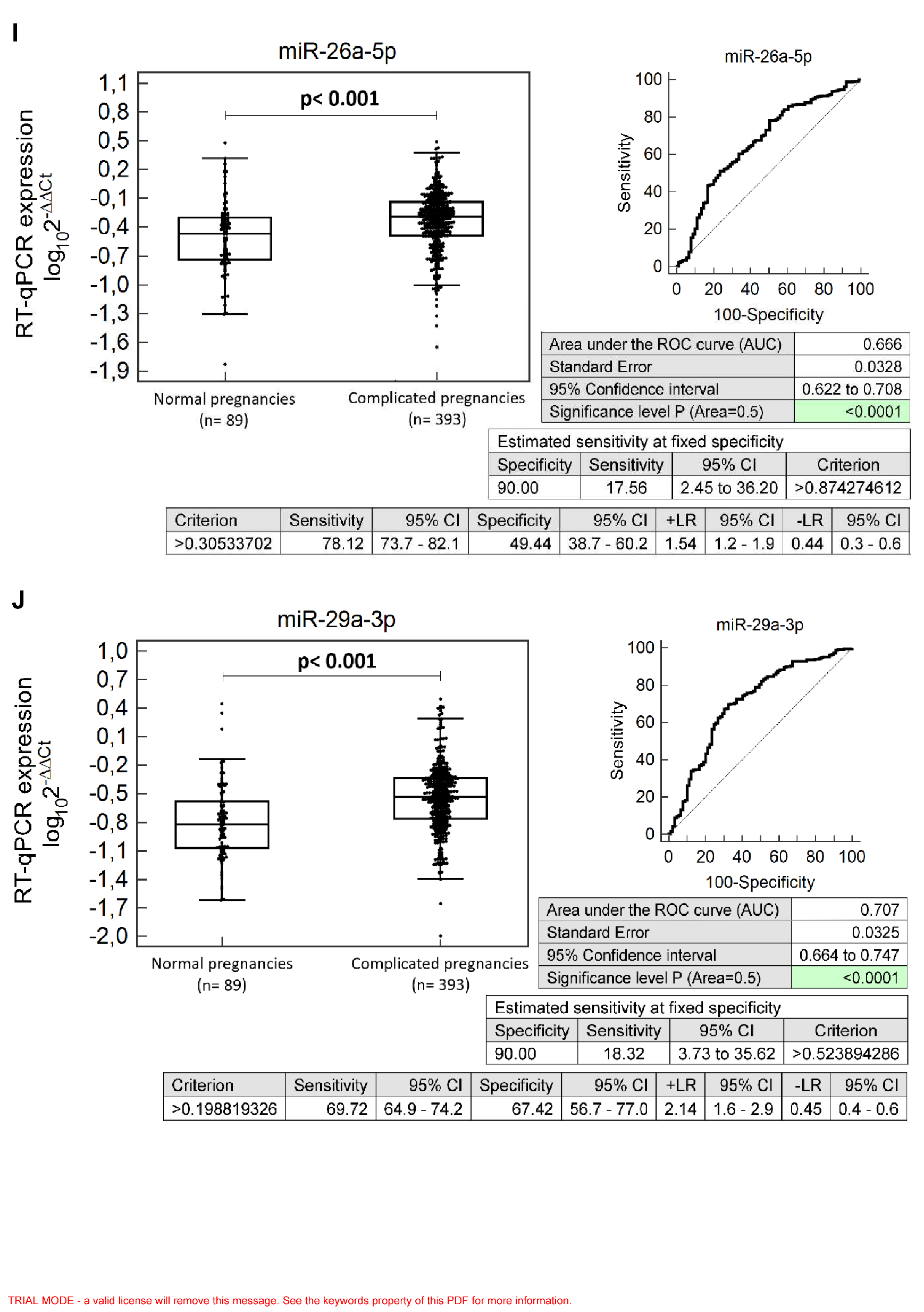


**
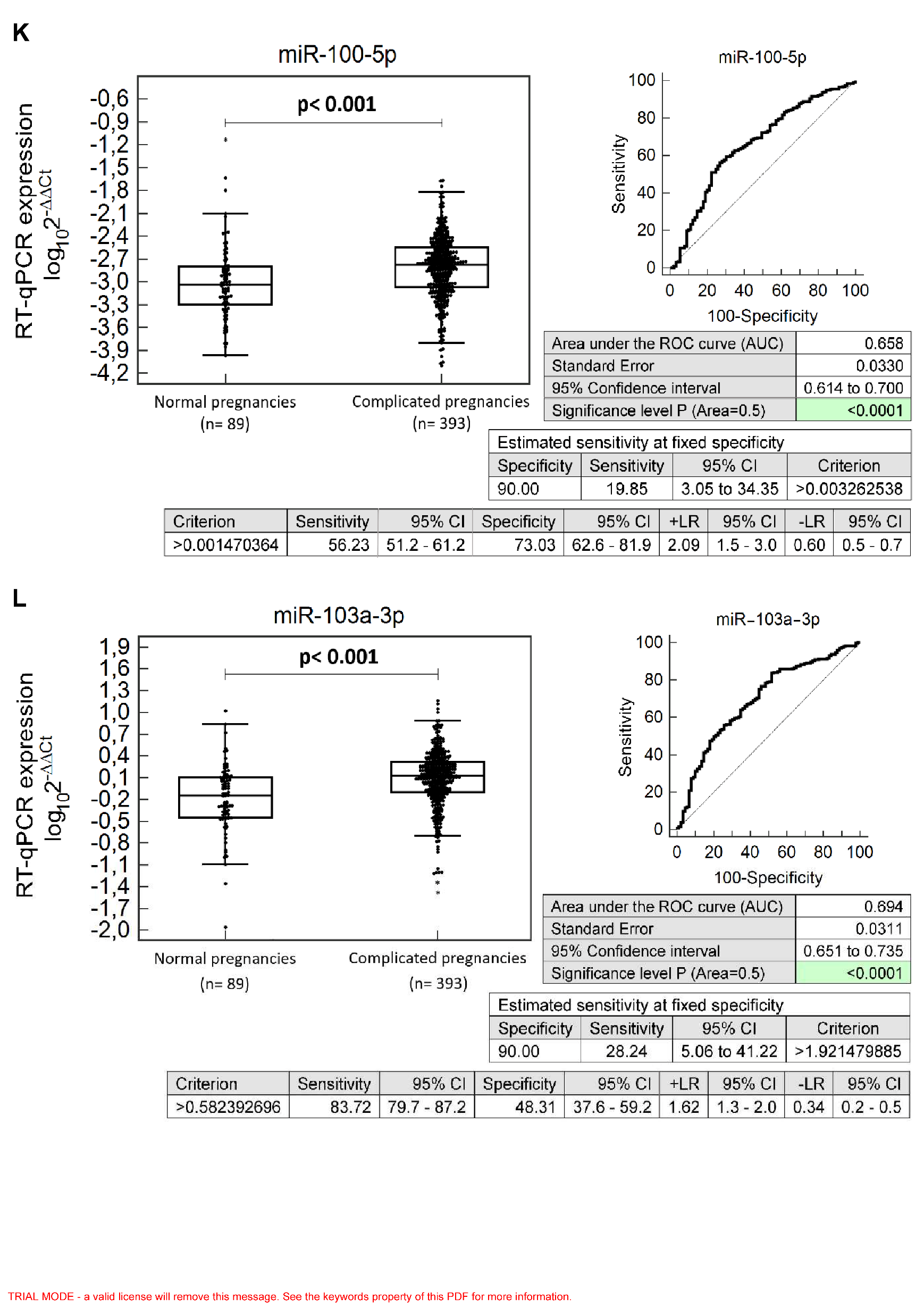
**

**
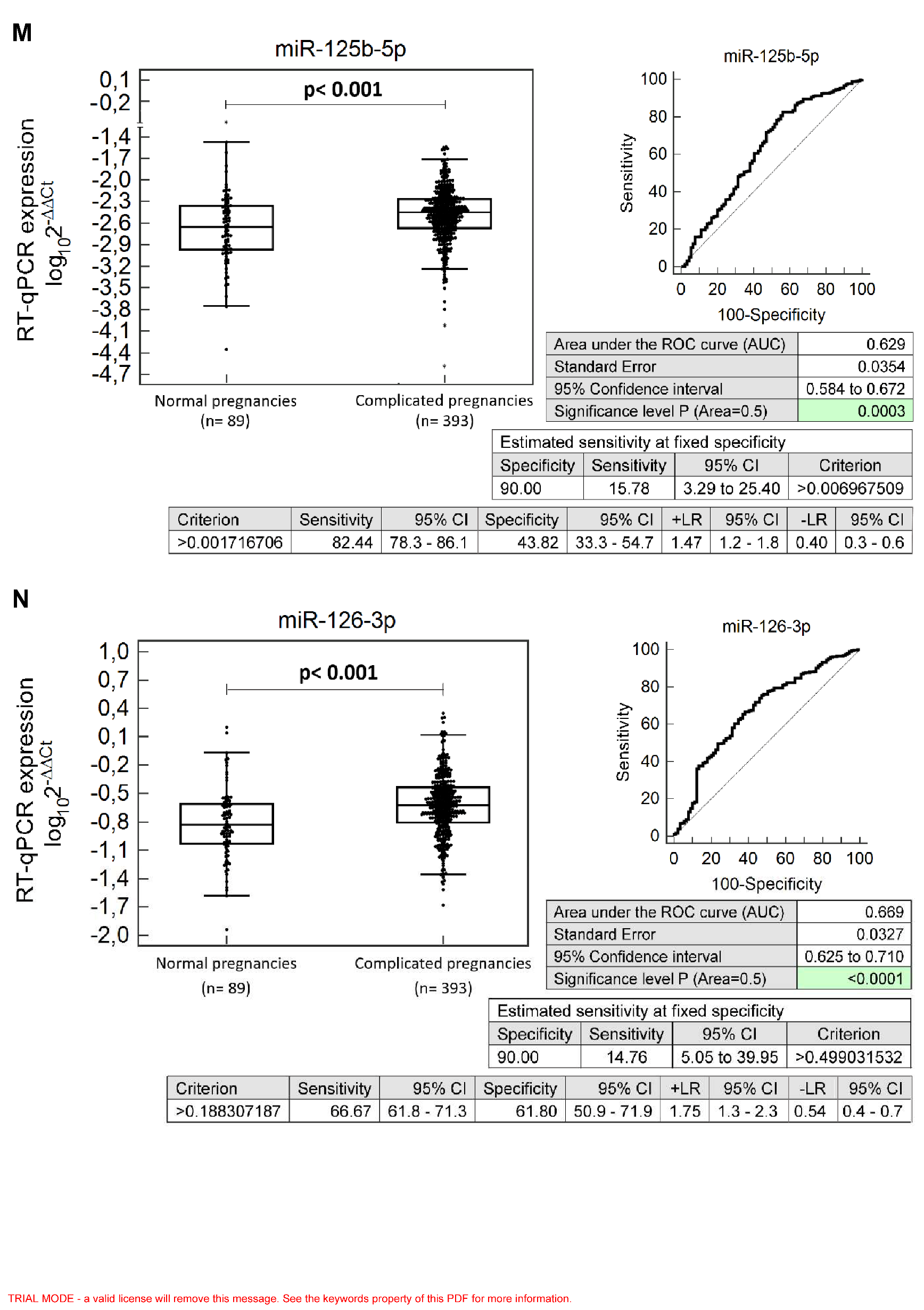
**

**
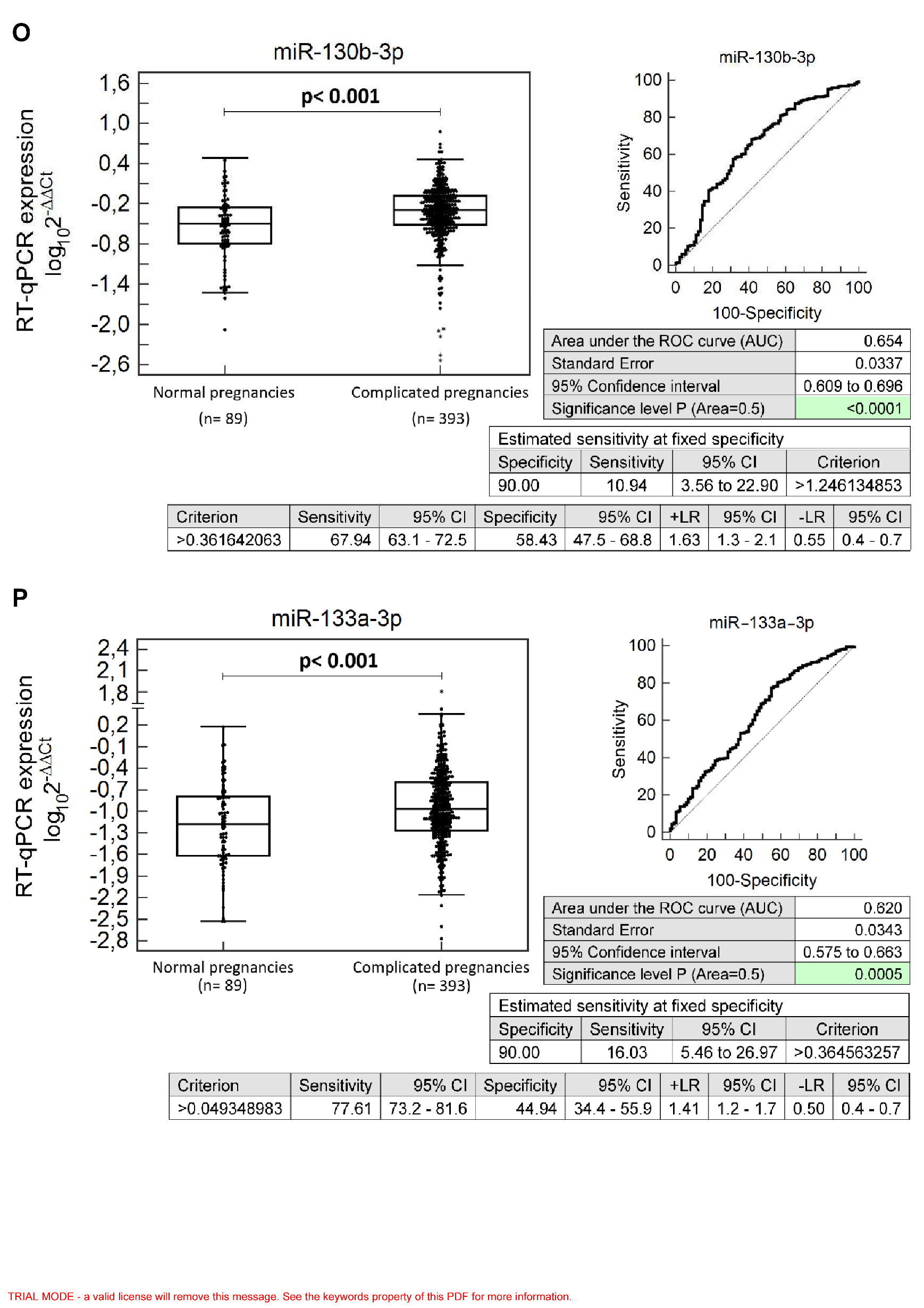
**

**
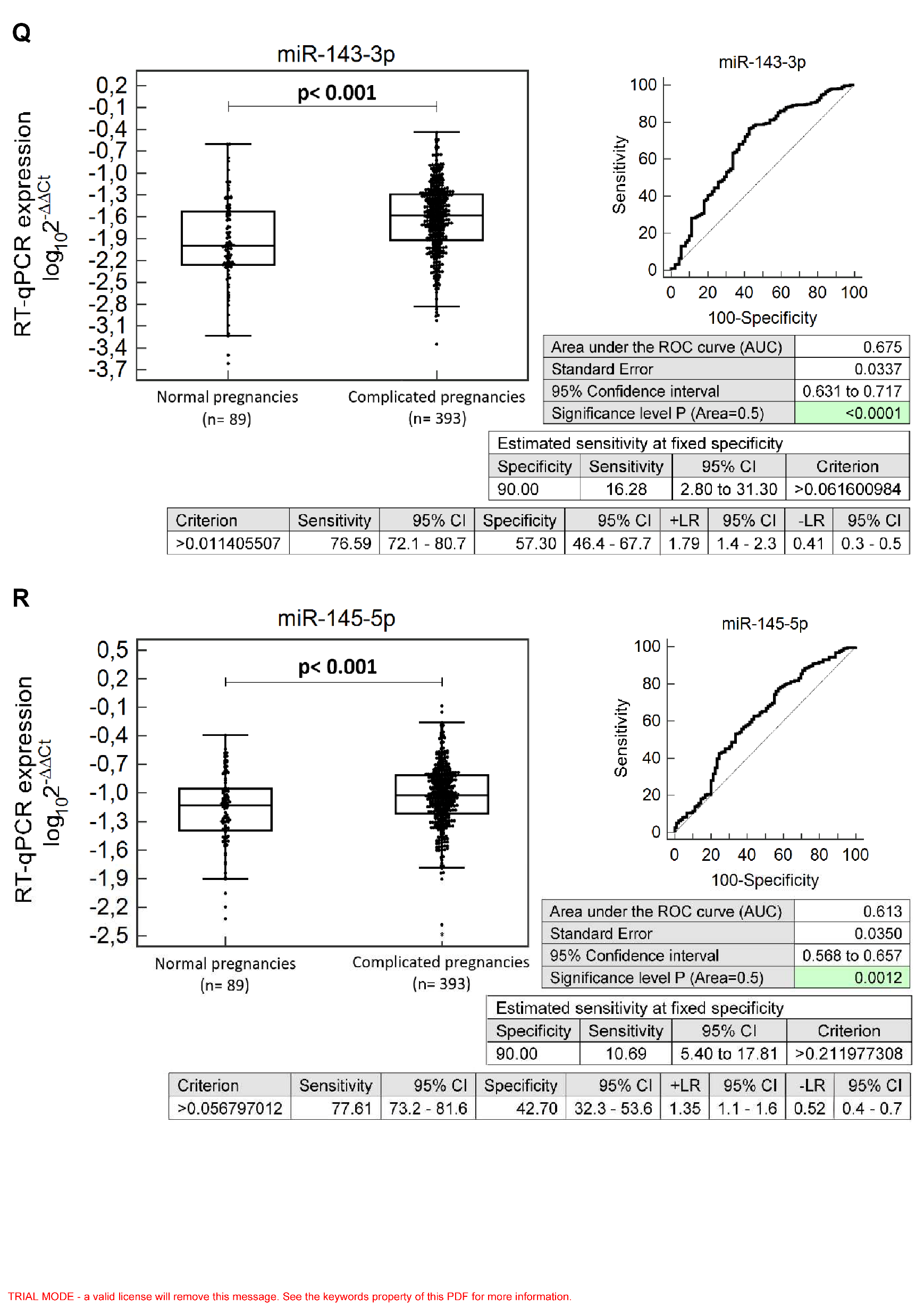
**

**
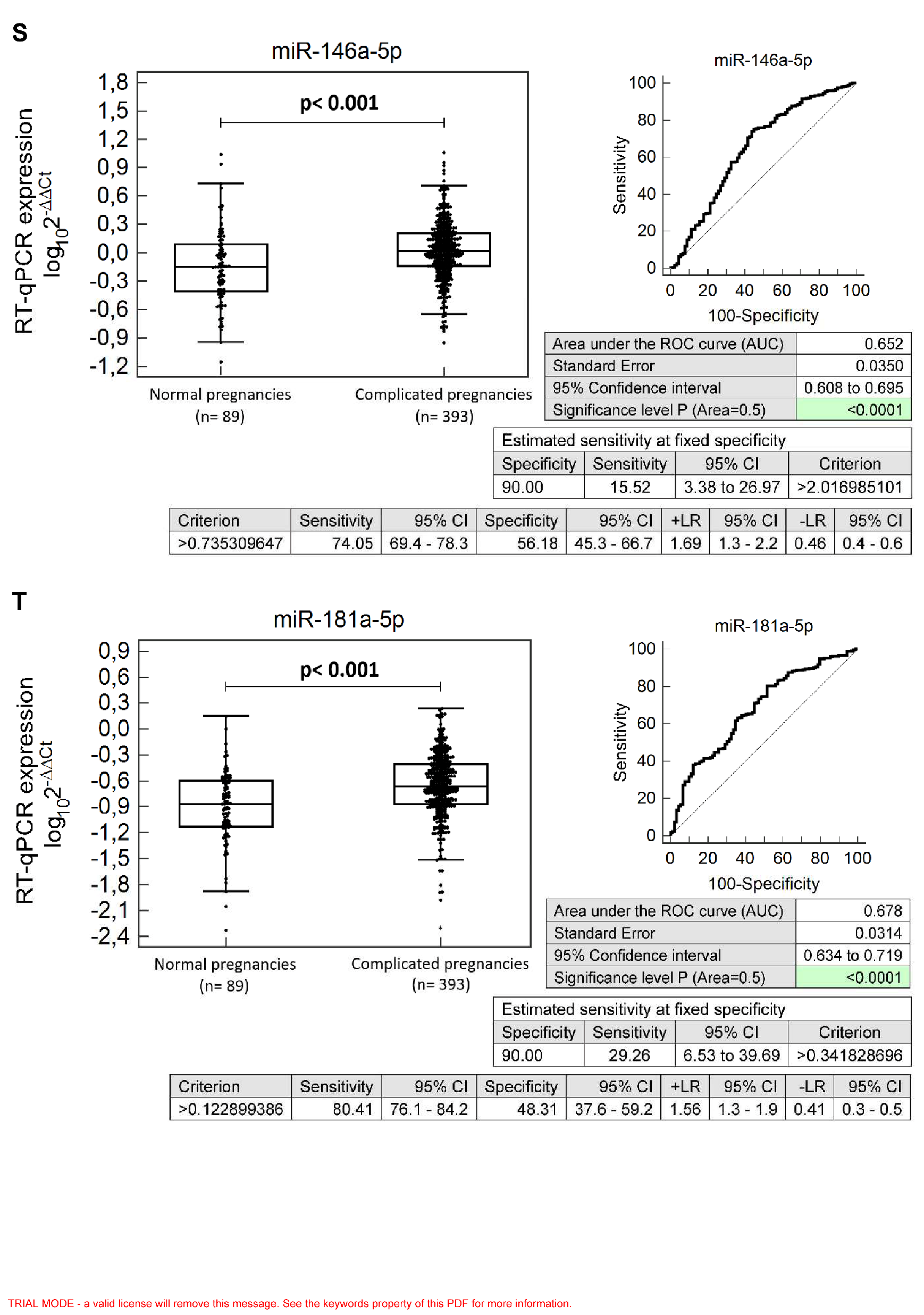
**

**
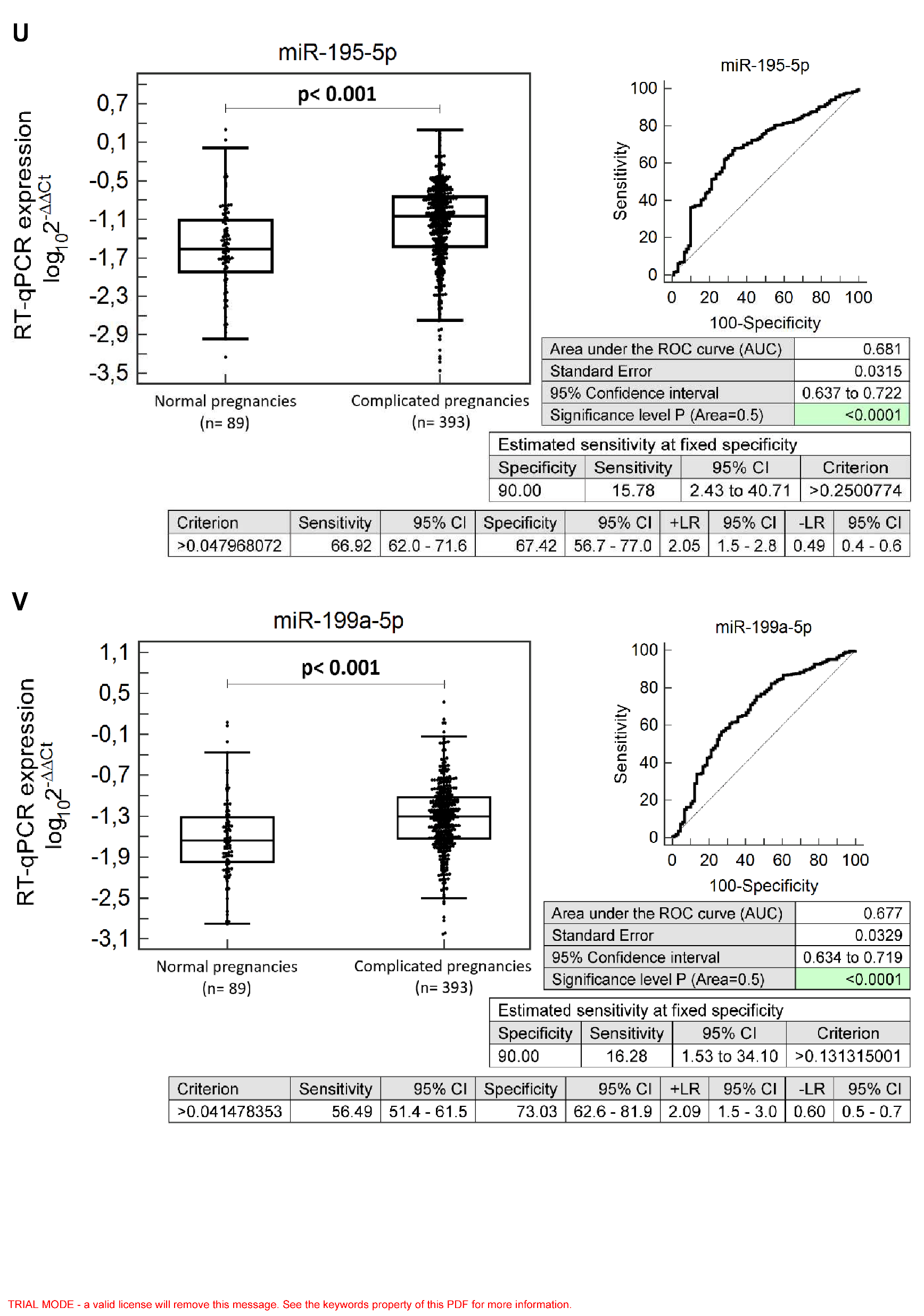
**

**
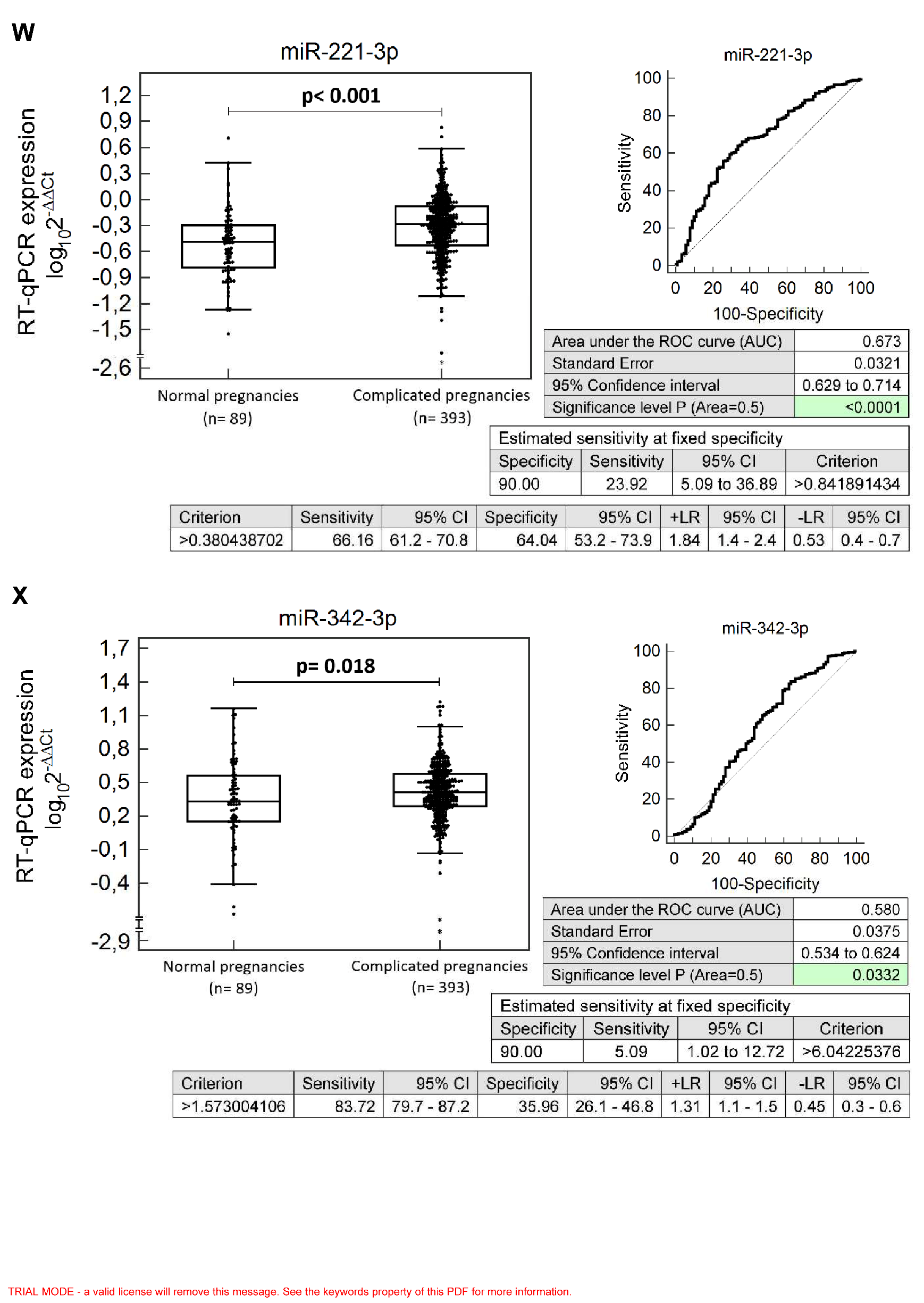
**

**
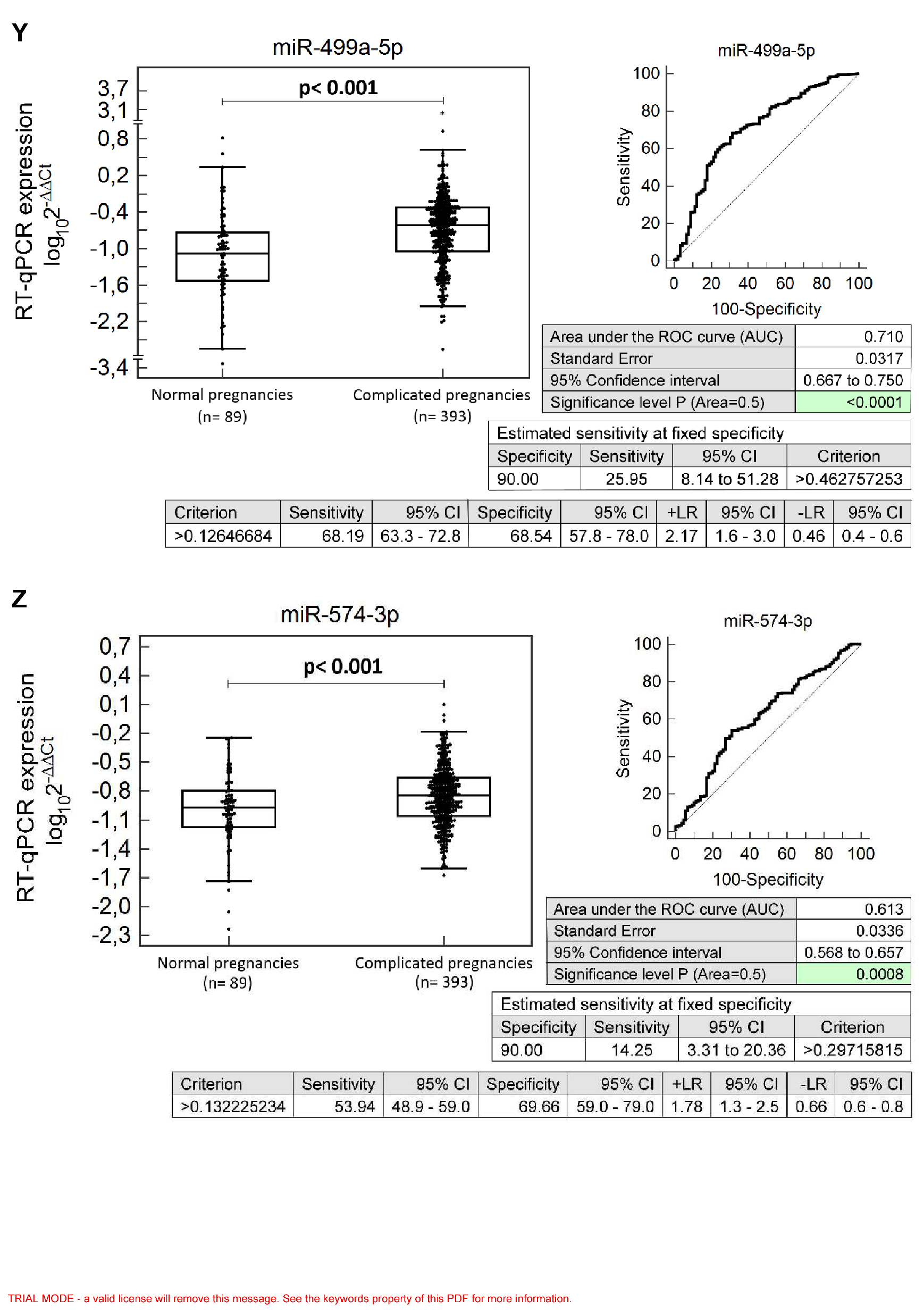
**

**Figure S1:** Aberrant microRNA expression profile in mothers after complicated pregnancies (gestational diabetes mellitus, gestational hypertension, preeclampsia, fetal growth restriction, preterm prelabor rupture of membranes, and/or spontaneous preterm birth). Mothers with a history of any pregnancy-related complication had substantially altered microRNA expression profile of miR-1-3p, miR-16-5p, miR-17-5p, miR-20a-5p, miR-20b-5p, miR-21-5p, miR-23a-3p, miR-24-3p, miR-26a-5p, miR-29a-3p, miR-100-5p, miR-103a-3p, miR-125b-5p, miR-126-3p, miR-130b-3p, miR-133a-3p, miR-143-3p, miR-145-5p, miR-146-5p, miR-181a-5p, miR-195-5p, miR-199a-5p, miR-221-3p, miR-342-3p, miR-499a-5p, and miR-574-3p.
