# Supplementary material for: Association Analysis in Young and Middle-Aged Mothers—Relation between Expression of Cardiovascular Disease Associated MicroRNAs and Abnormal Clinical Findings
Source: J Pers Med. 2021 Jan 11;11(1):39. doi: 10.3390/jpm11010039 (PMC7826744; doi:10.3390/jpm11010039)
Supplement: Supplementary file 1 [file jpm-11-00039-s001.zip › Supplementary Material/Supplementary Figure S10.docx]

**
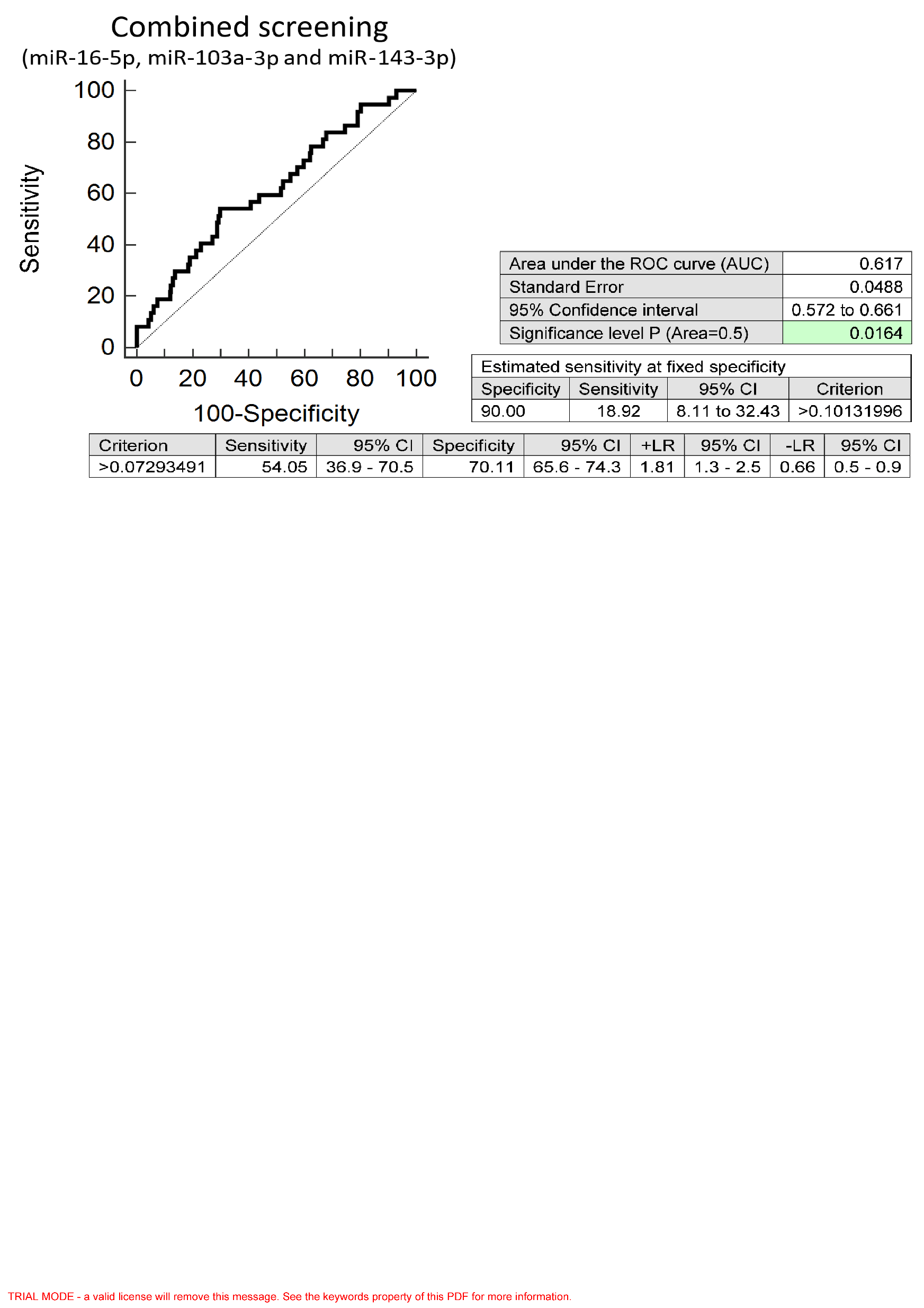
Supplementary Figure S10.**

**Figure S10:** Aberrant microRNA expression profile in mothers with the presence of trombophilic gene mutations. Irrespective of the course of previous pregnancies (normal and complicated pregnancies altogether), screening based on combination of miR-16-5p, miR-103a-3p, and miR-143-3p showed the best performance from various microRNA combinations. At 10.0% FPR 18.92% mothers with the presence of trombophilic gene mutations had substantially altered expression profile of miR-16-5p, miR-103a-3p, and miR-143-3p.
