# Supplementary material for: Association Analysis in Young and Middle-Aged Mothers—Relation between Expression of Cardiovascular Disease Associated MicroRNAs and Abnormal Clinical Findings
Source: J Pers Med. 2021 Jan 11;11(1):39. doi: 10.3390/jpm11010039 (PMC7826744; doi:10.3390/jpm11010039)
Supplement: Supplementary file 1 [file jpm-11-00039-s001.zip › Supplementary Material/Supplementary Figure S11.docx]

**Supplementary Figure S11.**

**
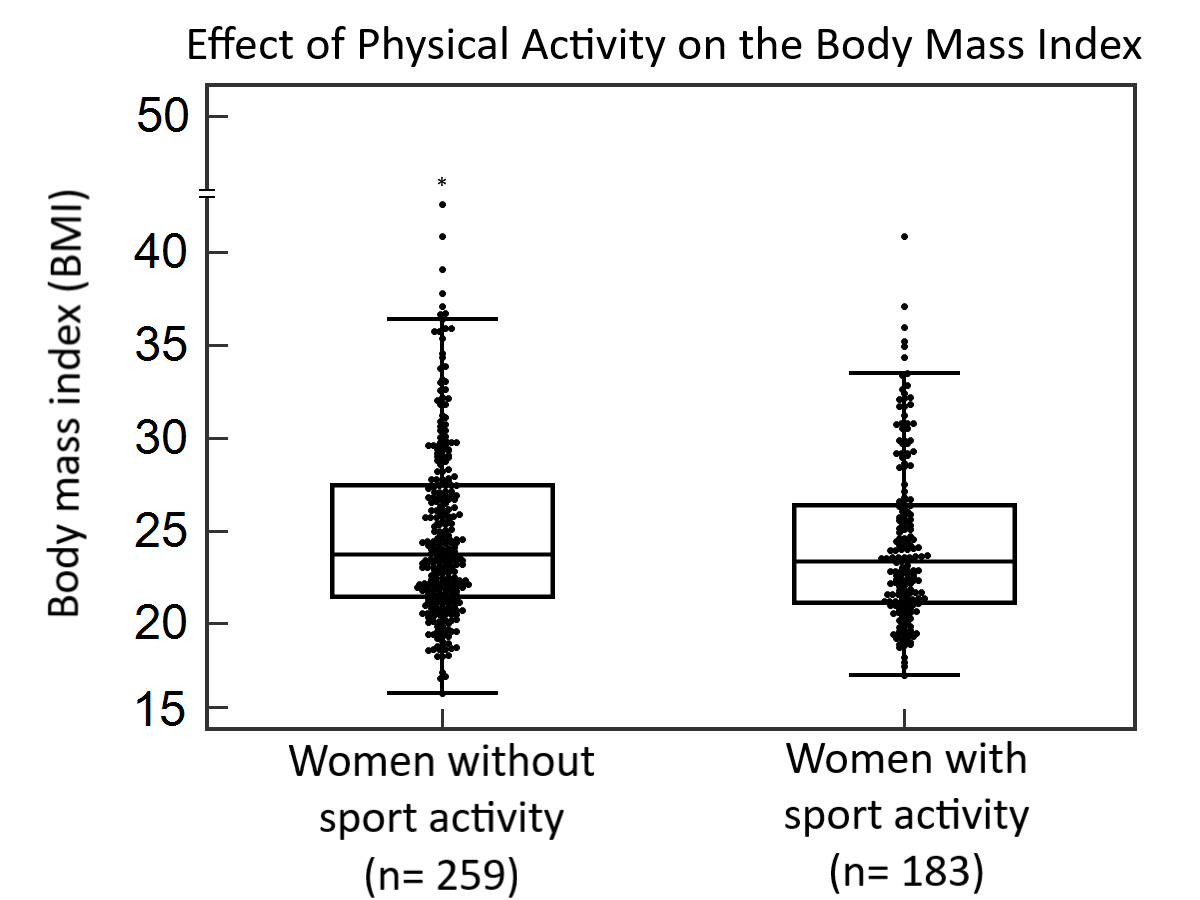
**

**Figure S11:** Association analysis between physical activity and BMI values showed decreased levels of BMI values in women used to run any sport activity from time to time, however the analysis has not yet achieved the statistical significance (Mann-Whitney test, p= 0.186).
