# Supplementary material for: Association Analysis in Young and Middle-Aged Mothers—Relation between Expression of Cardiovascular Disease Associated MicroRNAs and Abnormal Clinical Findings
Source: J Pers Med. 2021 Jan 11;11(1):39. doi: 10.3390/jpm11010039 (PMC7826744; doi:10.3390/jpm11010039)
Supplement: Supplementary file 1 [file jpm-11-00039-s001.zip › Supplementary Material/Supplementary Figure S13.docx]

**Supplementary Figure S13.**

**
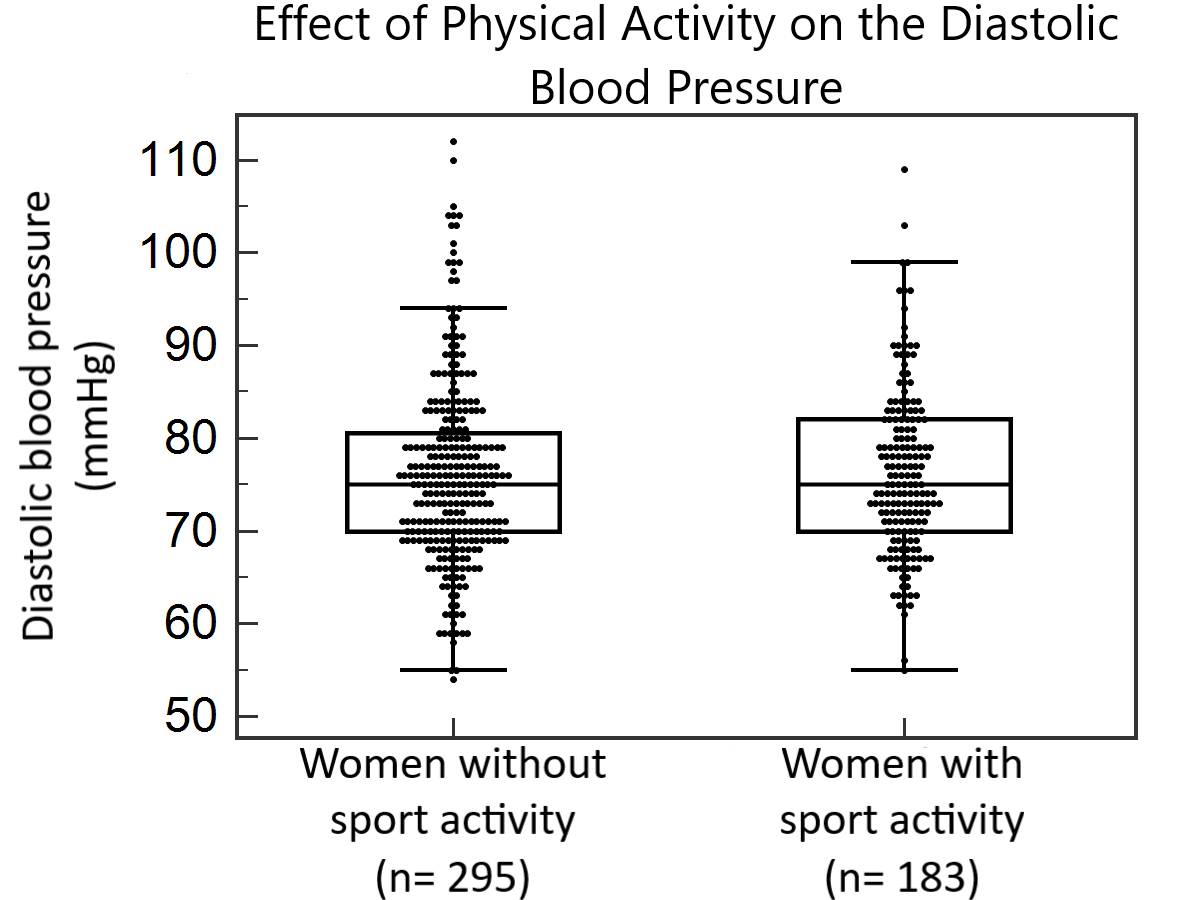
**

**Figure S13:** Occasional physical activity had no impact on diastolic blood pressure (DBP) values (Mann-Whitney test, p= 0.735) in our cohort of patients.
