# Supplementary material for: Association Analysis in Young and Middle-Aged Mothers—Relation between Expression of Cardiovascular Disease Associated MicroRNAs and Abnormal Clinical Findings
Source: J Pers Med. 2021 Jan 11;11(1):39. doi: 10.3390/jpm11010039 (PMC7826744; doi:10.3390/jpm11010039)
Supplement: Supplementary file 1 [file jpm-11-00039-s001.zip › Supplementary Material/Supplementary Figure S2.docx]

**
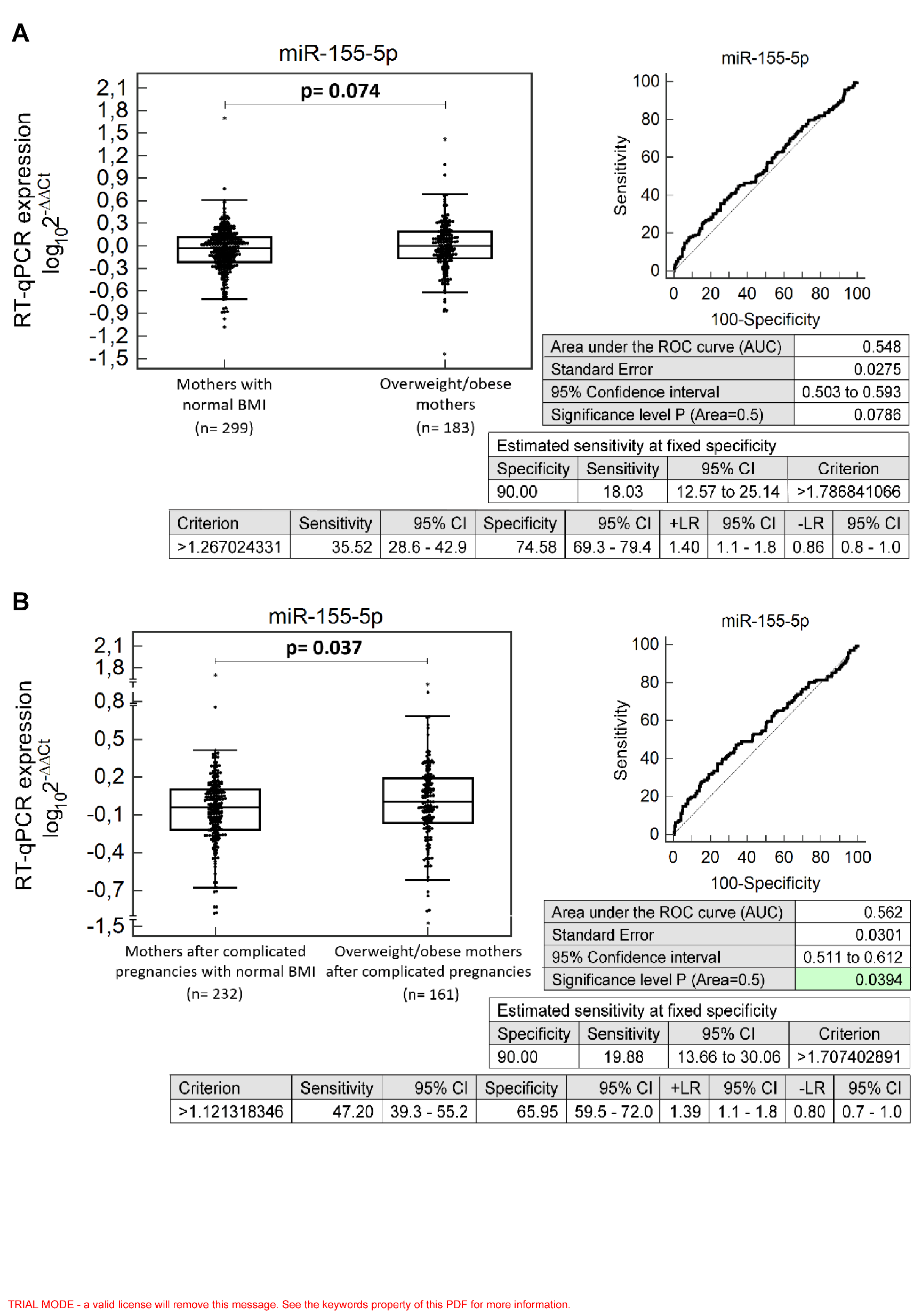
Supplementary Figure S2.**

**Figure S2:** Aberrant miR-155-5p expression profile in overweight/obese mothers. Irrespective of the course of previous pregnancies (normal and complicated pregnancies altogether), at 10.0% FPR 18.03% overweight/obese mothers showed up-regulation of miR-155-5p (A). 19.88% overweight/obese mothers after complicated pregnancies showed up-regulation of miR-155-5p (B).
