# Supplementary material for: Association Analysis in Young and Middle-Aged Mothers—Relation between Expression of Cardiovascular Disease Associated MicroRNAs and Abnormal Clinical Findings
Source: J Pers Med. 2021 Jan 11;11(1):39. doi: 10.3390/jpm11010039 (PMC7826744; doi:10.3390/jpm11010039)
Supplement: Supplementary file 1 [file jpm-11-00039-s001.zip › Supplementary Material/Supplementary Figure S3.docx]

**
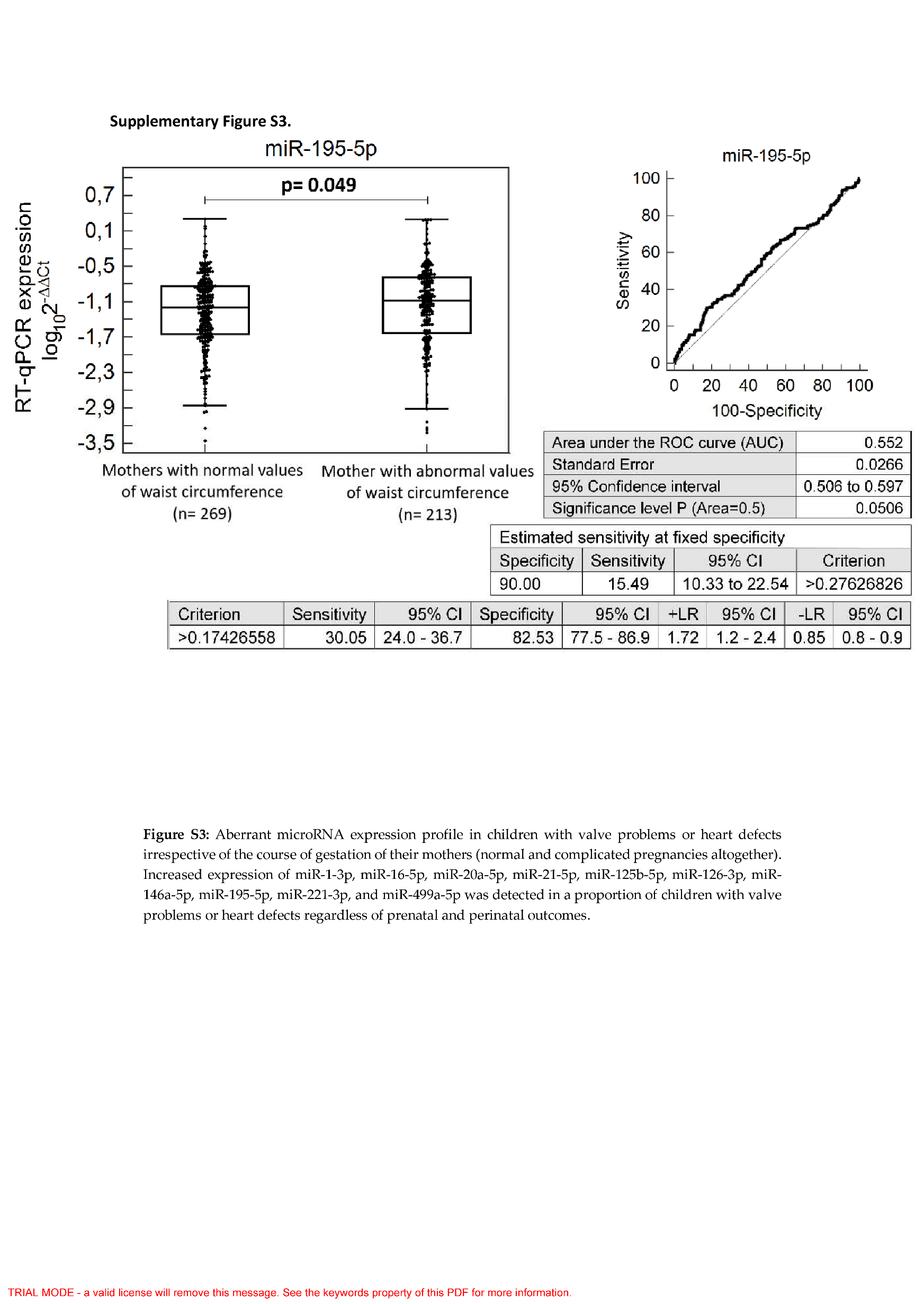
Supplementary Figure S3.**

**Figure S3:** Aberrant miR-195-5p expression profile in mothers with central obesity. Irrespective of the course of previous pregnancies (normal and complicated pregnancies altogether), at 10.0% FPR 15.49% mothers with central obesity showed up-regulation of miR-195-5p.
