# Supplementary material for: Association Analysis in Young and Middle-Aged Mothers—Relation between Expression of Cardiovascular Disease Associated MicroRNAs and Abnormal Clinical Findings
Source: J Pers Med. 2021 Jan 11;11(1):39. doi: 10.3390/jpm11010039 (PMC7826744; doi:10.3390/jpm11010039)
Supplement: Supplementary file 1 [file jpm-11-00039-s001.zip › Supplementary Material/Supplementary Figure S4.docx]

**
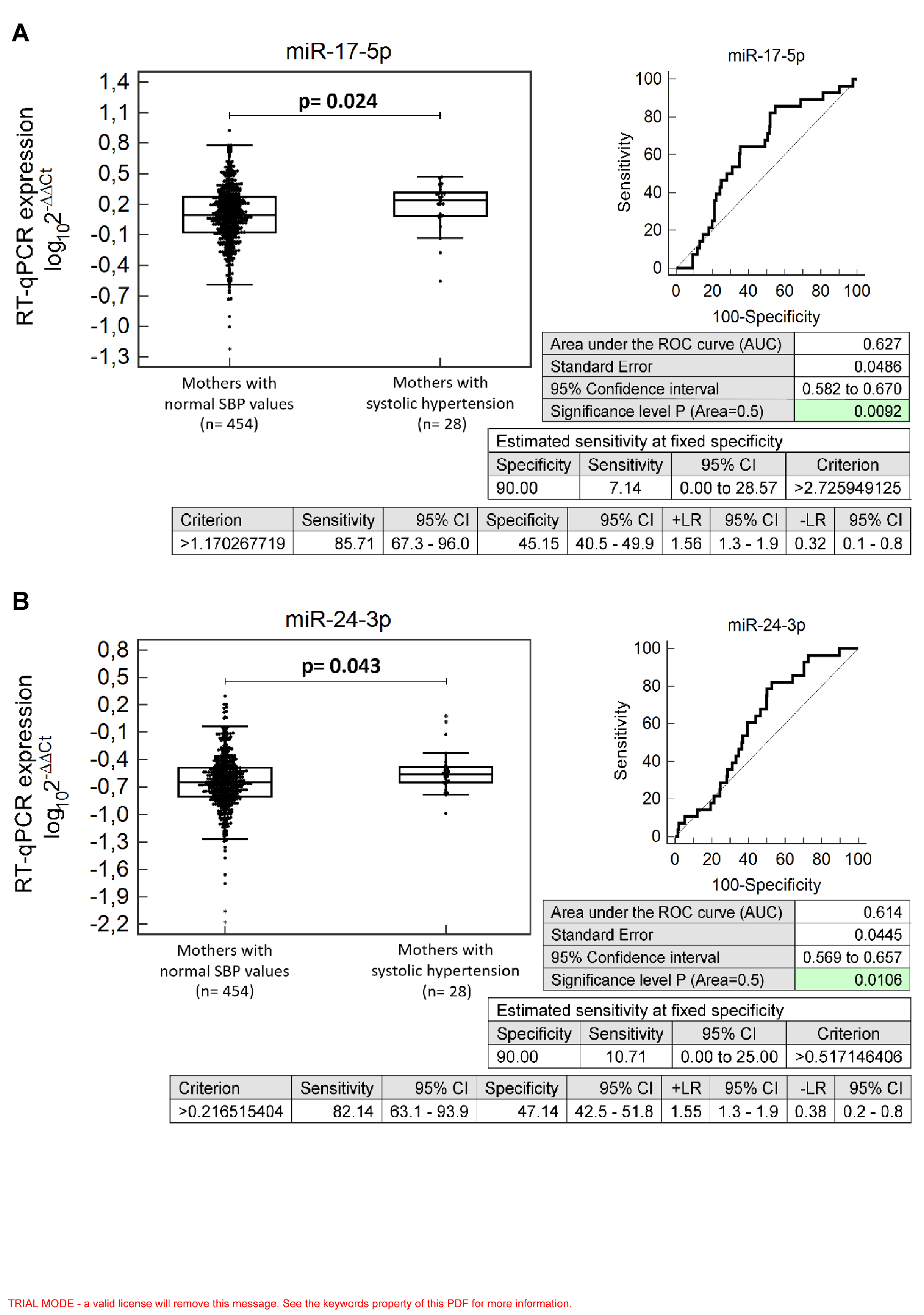
Supplementary Figure S4.**

**
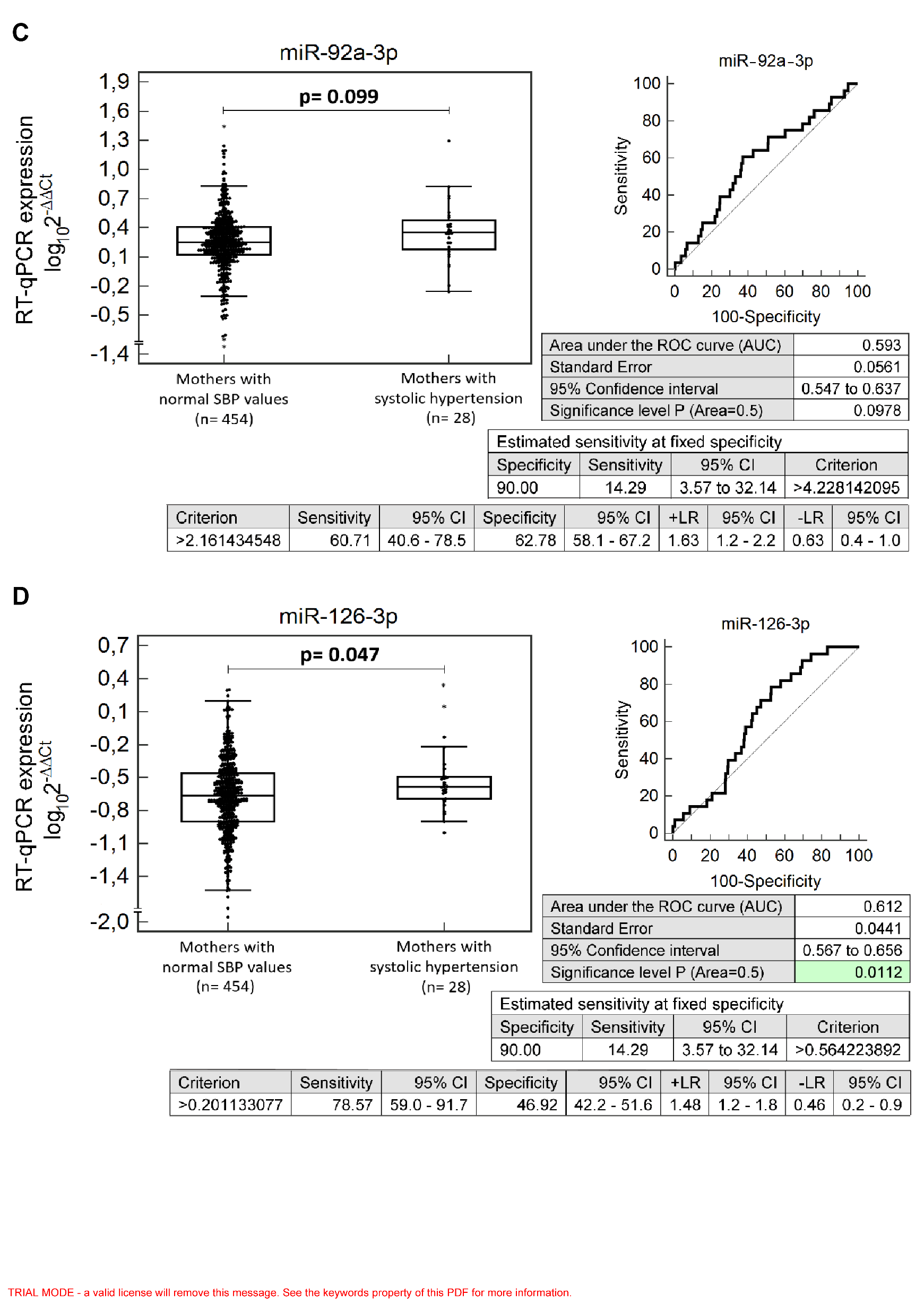
**

**
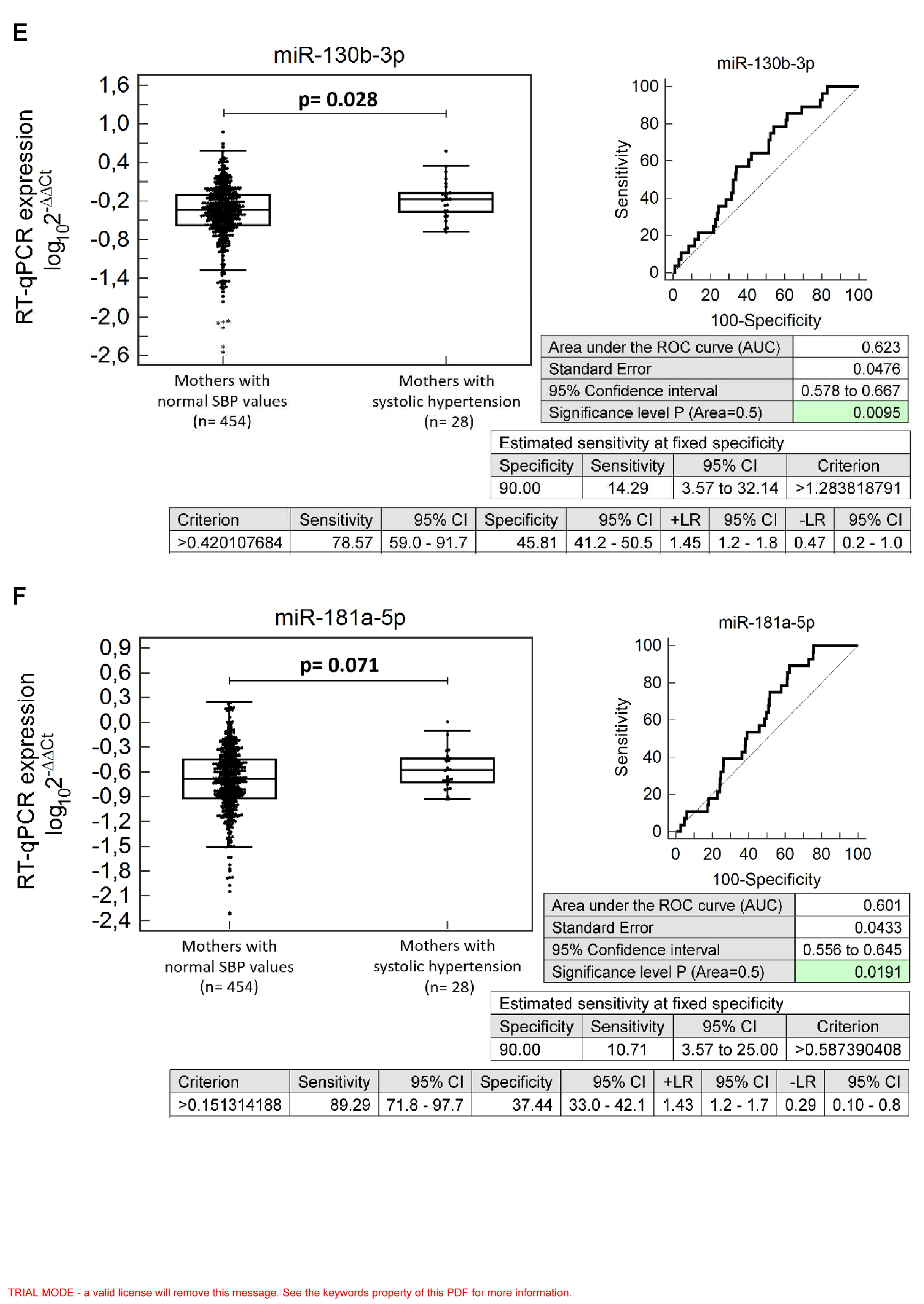
**

**
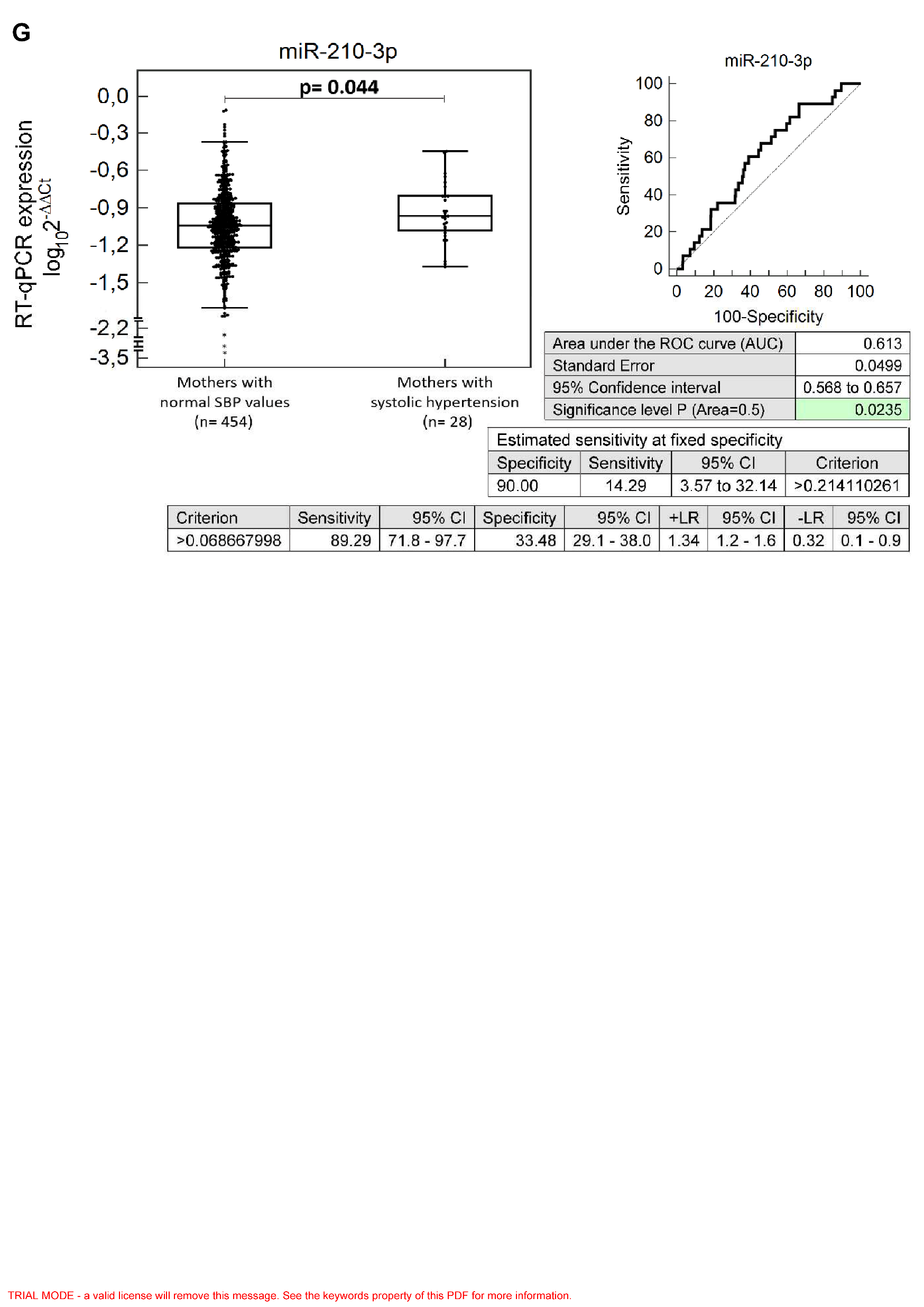
Figure S4:** Aberrant microRNA expression profile in mothers with systolic hypertension. Irrespective of the course of gestation (normal and complicated pregnancies altogether), at 10.0% FPR a proportion of mothers with systolic hypertension had substantially altered expression profile of miR-17-5p, miR-24-3p, miR-92a-3p, miR-126-3p, miR-130b-3p, miR-181a-5p, and miR-210-3p.
