# Supplementary material for: Association Analysis in Young and Middle-Aged Mothers—Relation between Expression of Cardiovascular Disease Associated MicroRNAs and Abnormal Clinical Findings
Source: J Pers Med. 2021 Jan 11;11(1):39. doi: 10.3390/jpm11010039 (PMC7826744; doi:10.3390/jpm11010039)
Supplement: Supplementary file 1 [file jpm-11-00039-s001.zip › Supplementary Material/Supplementary Figure S7.docx]

**
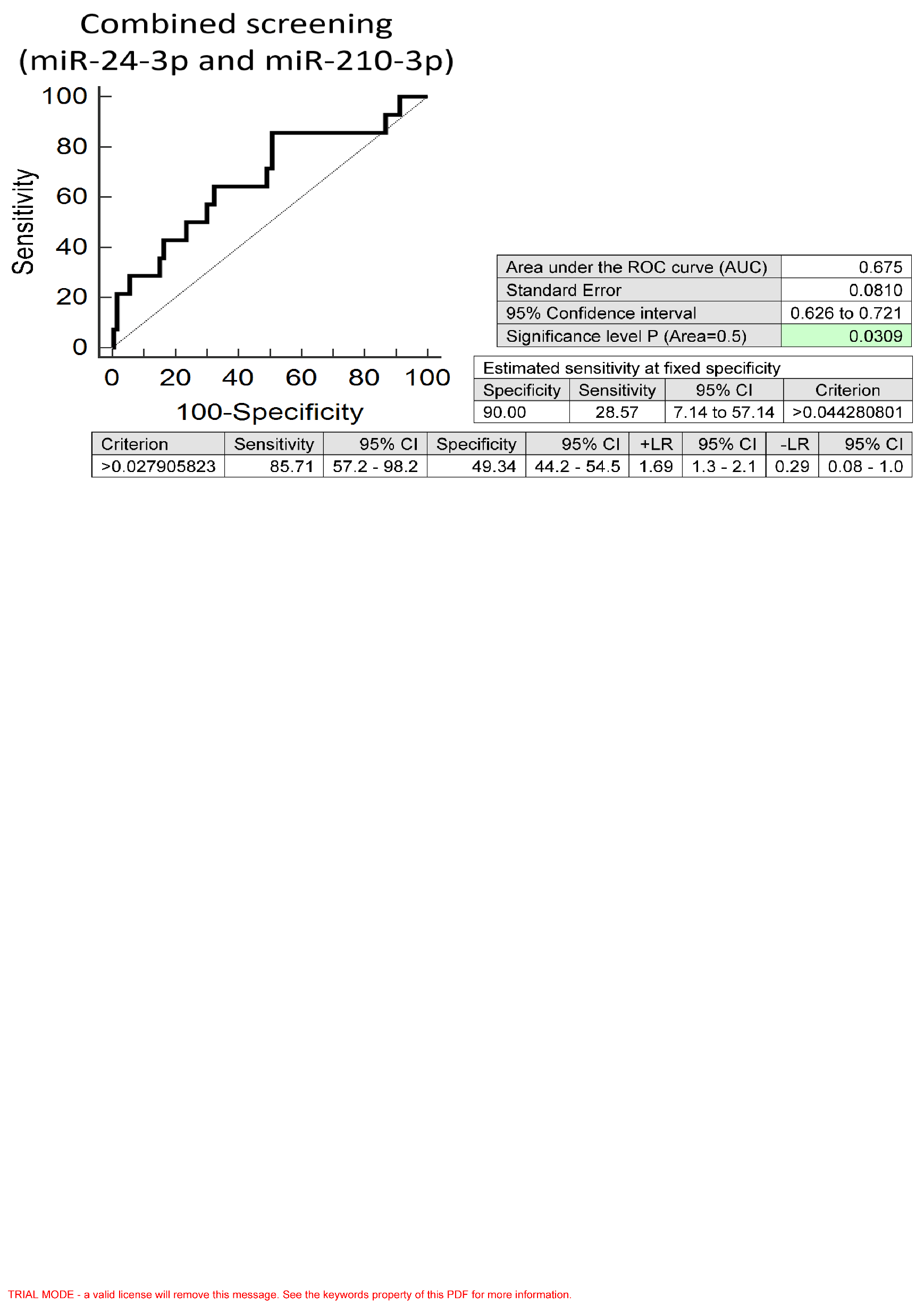
Supplementary Figure S7.**

**Figure S7:** Aberrant microRNA expression profile in mothers on blood pressure treatment. In women after complicated pregnancies only, screening based on combination of miR-24-3p and miR-210-3p showed the best performance from various microRNA combinations. At 10.0% FPR 28.57% mothers on blood pressure treatment had substantially altered expression profile of miR-24-3p and miR-210-3p.
