# Supplementary material for: Association Analysis in Young and Middle-Aged Mothers—Relation between Expression of Cardiovascular Disease Associated MicroRNAs and Abnormal Clinical Findings
Source: J Pers Med. 2021 Jan 11;11(1):39. doi: 10.3390/jpm11010039 (PMC7826744; doi:10.3390/jpm11010039)
Supplement: Supplementary file 1 [file jpm-11-00039-s001.zip › Supplementary Material/Supplementary Figure S8.docx]

**
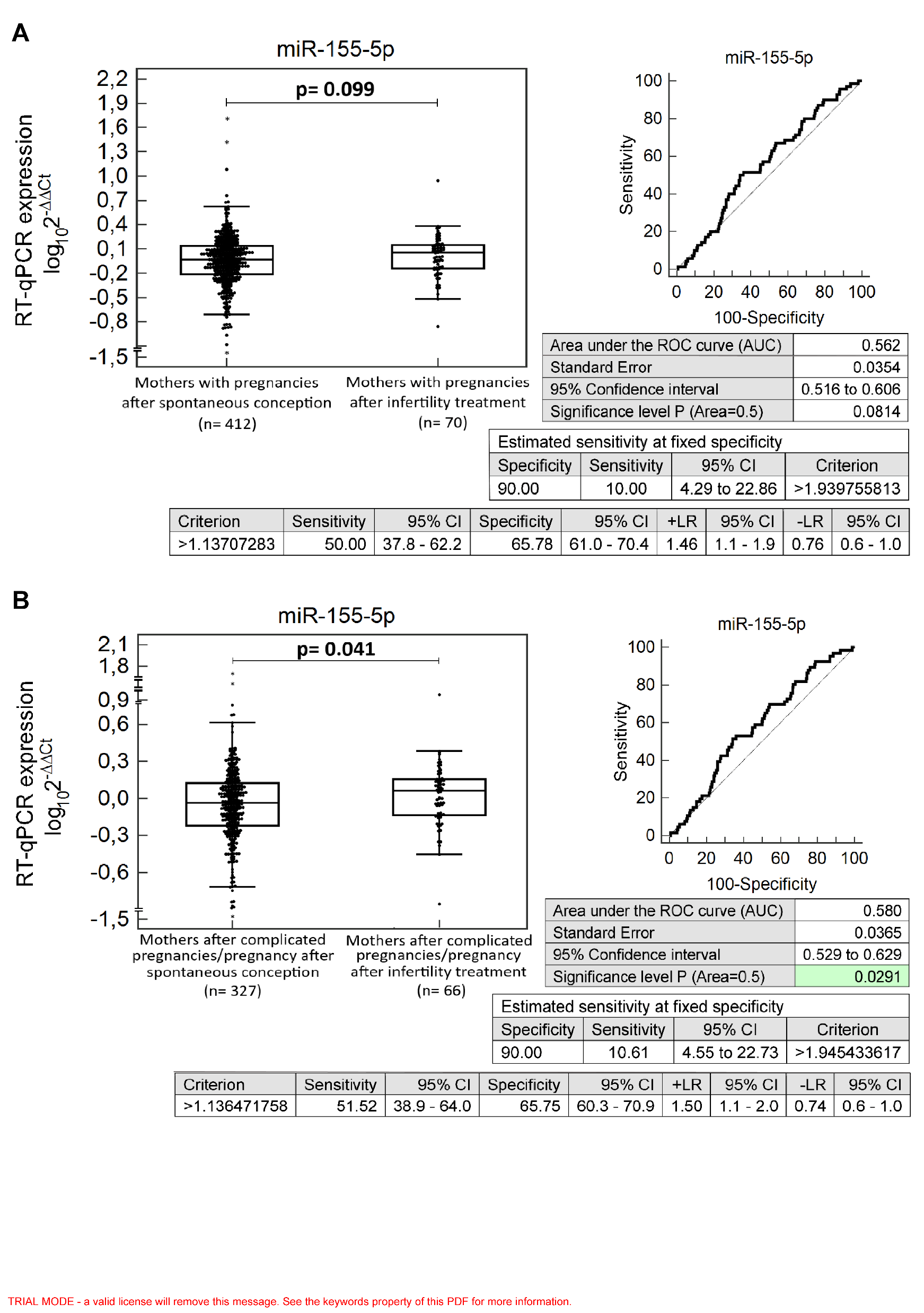
Supplementary Figure S8.**

**Figure S8:** Aberrant miR-155-5p expression profile in mothers with pregnancies after infertility treatment. Irrespective of the course of previous pregnancies (normal and complicated pregnancies altogether), at 10.0% FPR 10.0% mothers with pregnancies after infertility treatment showed up-regulation of miR-155-5p (A). 10.61% mothers after complicated pregnancies, who passed infertility treatment programme, showed up-regulation of miR-155-5p (B).
