# Supplementary material for: Association Analysis in Young and Middle-Aged Mothers—Relation between Expression of Cardiovascular Disease Associated MicroRNAs and Abnormal Clinical Findings
Source: J Pers Med. 2021 Jan 11;11(1):39. doi: 10.3390/jpm11010039 (PMC7826744; doi:10.3390/jpm11010039)
Supplement: Supplementary file 1 [file jpm-11-00039-s001.zip › Supplementary Material/Supplementary Figure S9.docx]

**
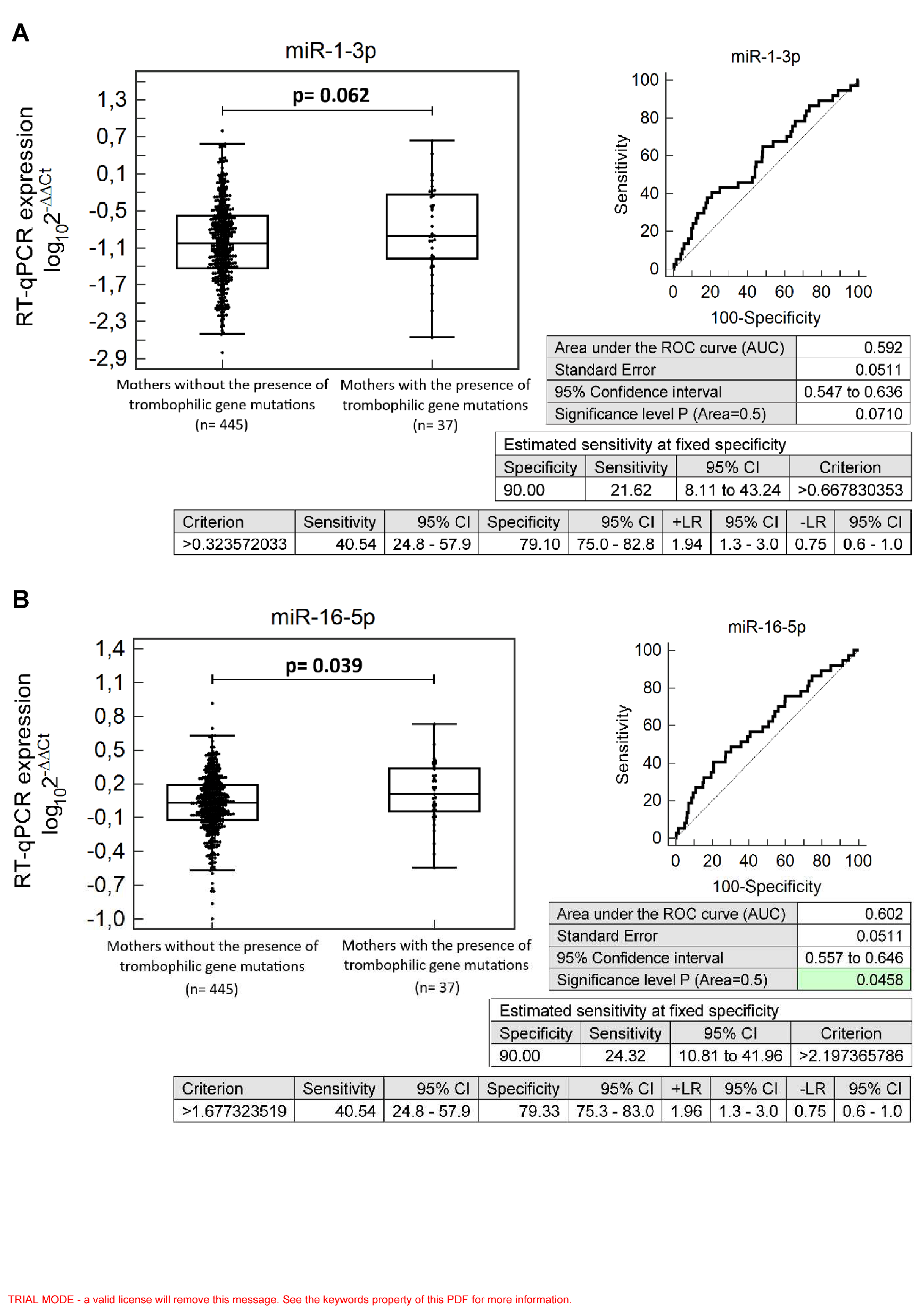
Supplementary Figure S9.**


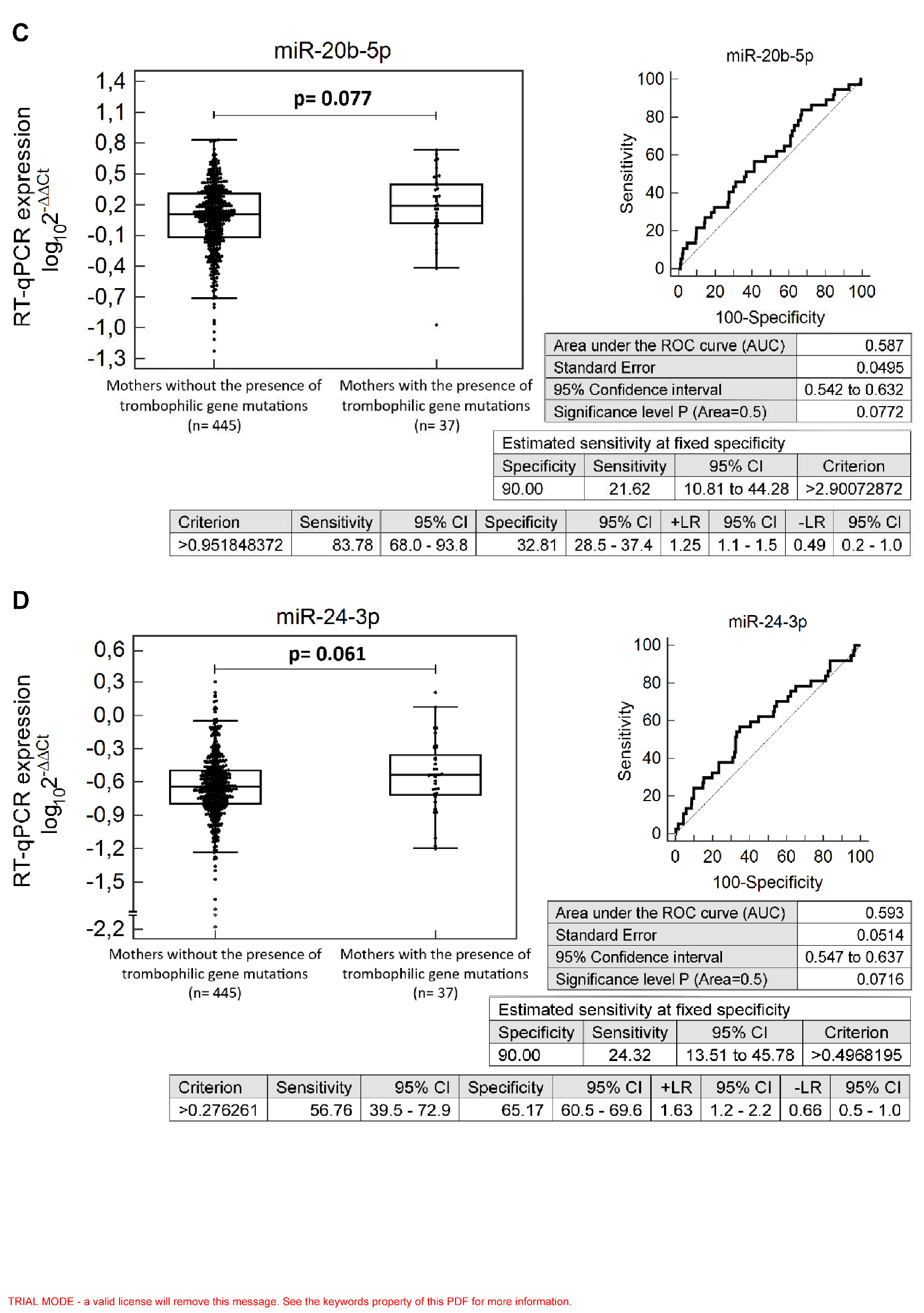


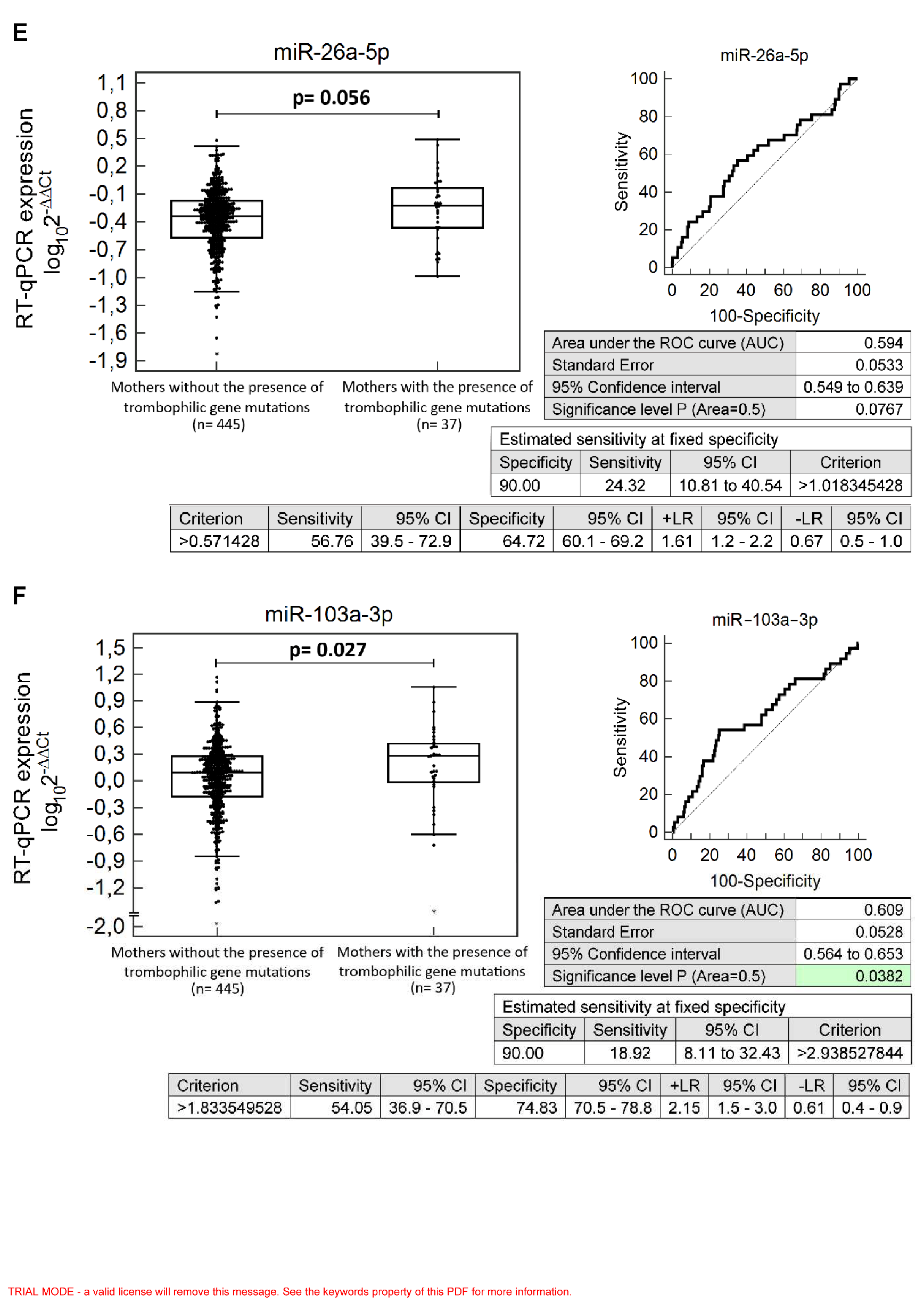


**
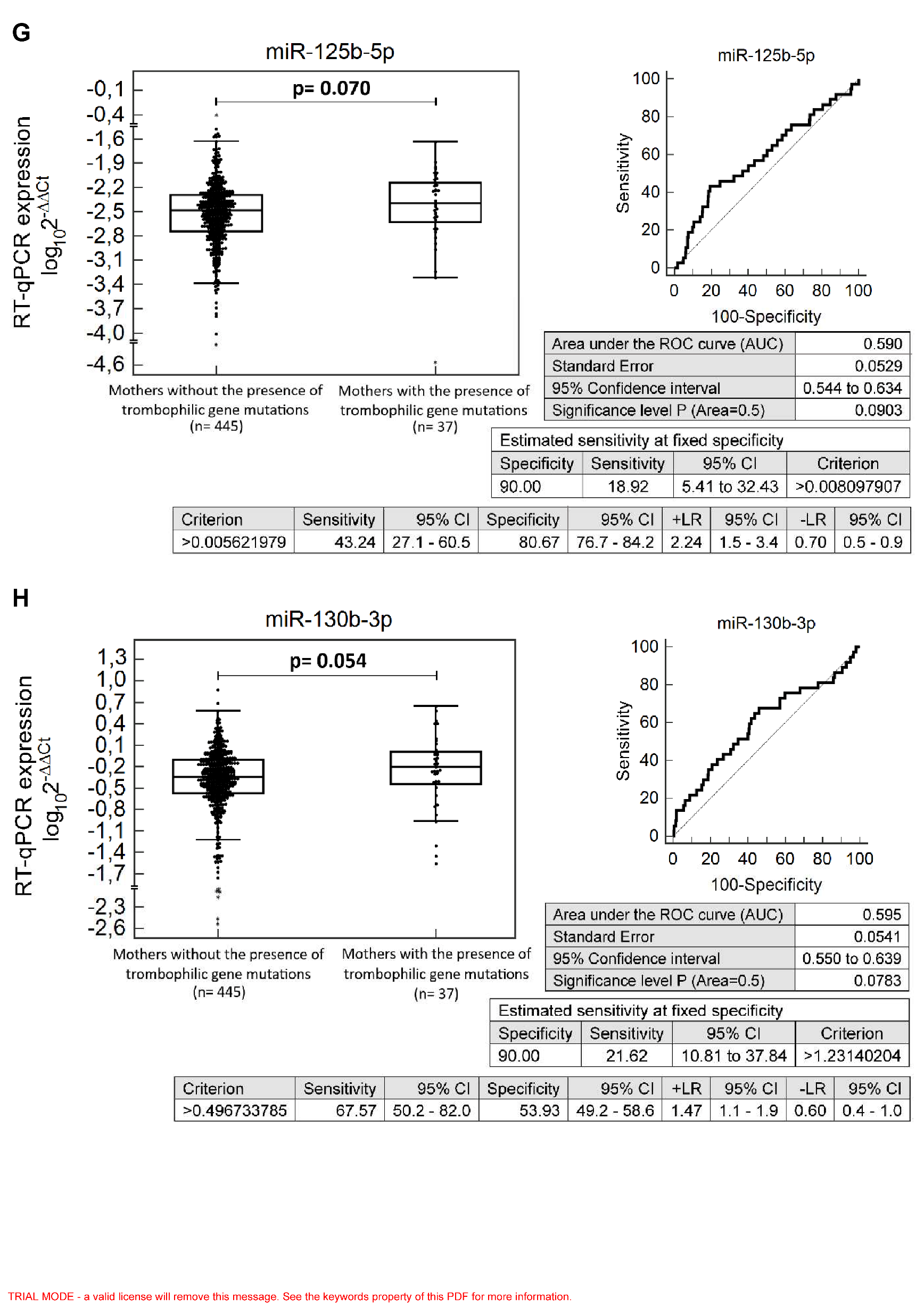
**

**
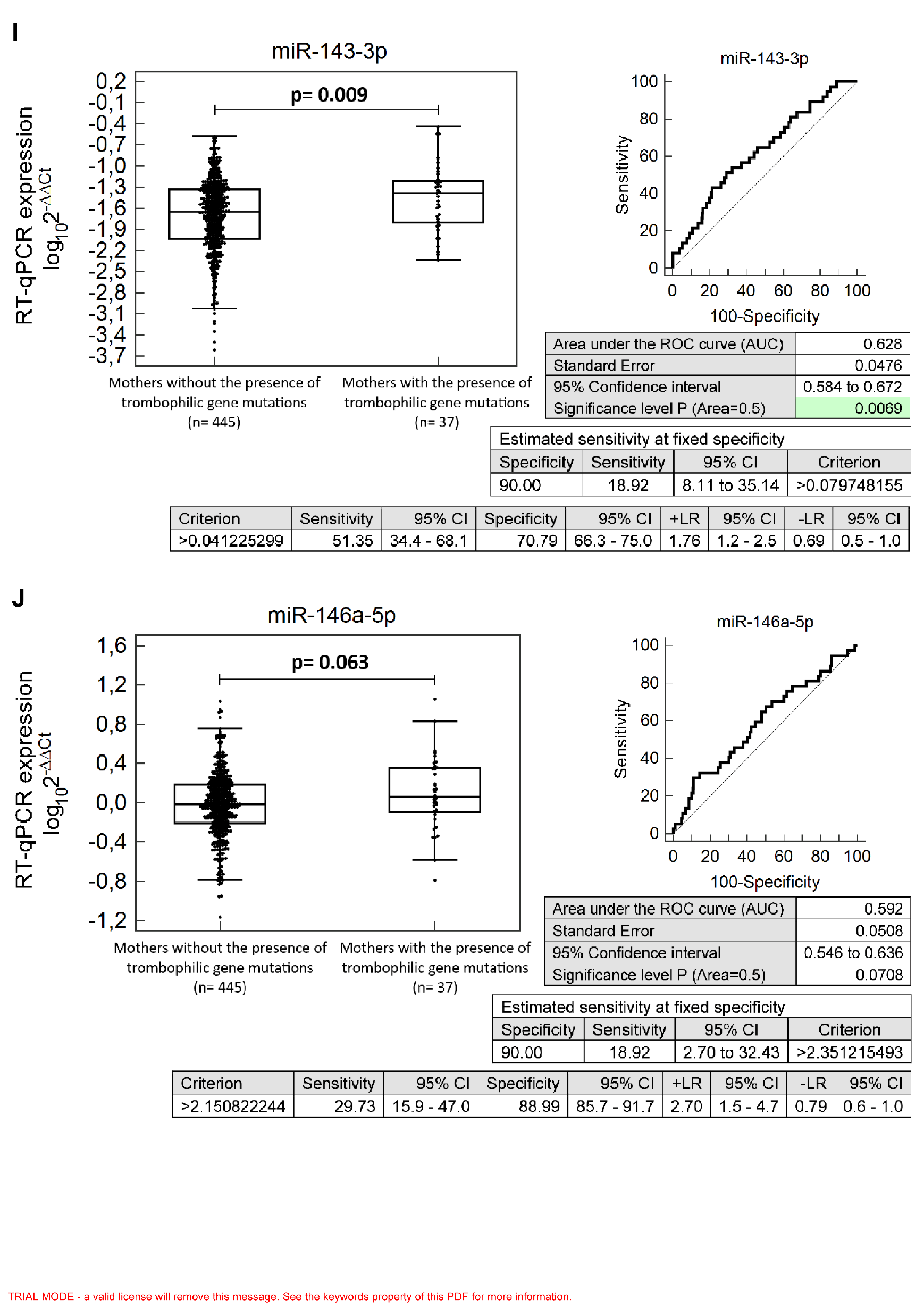
**

**
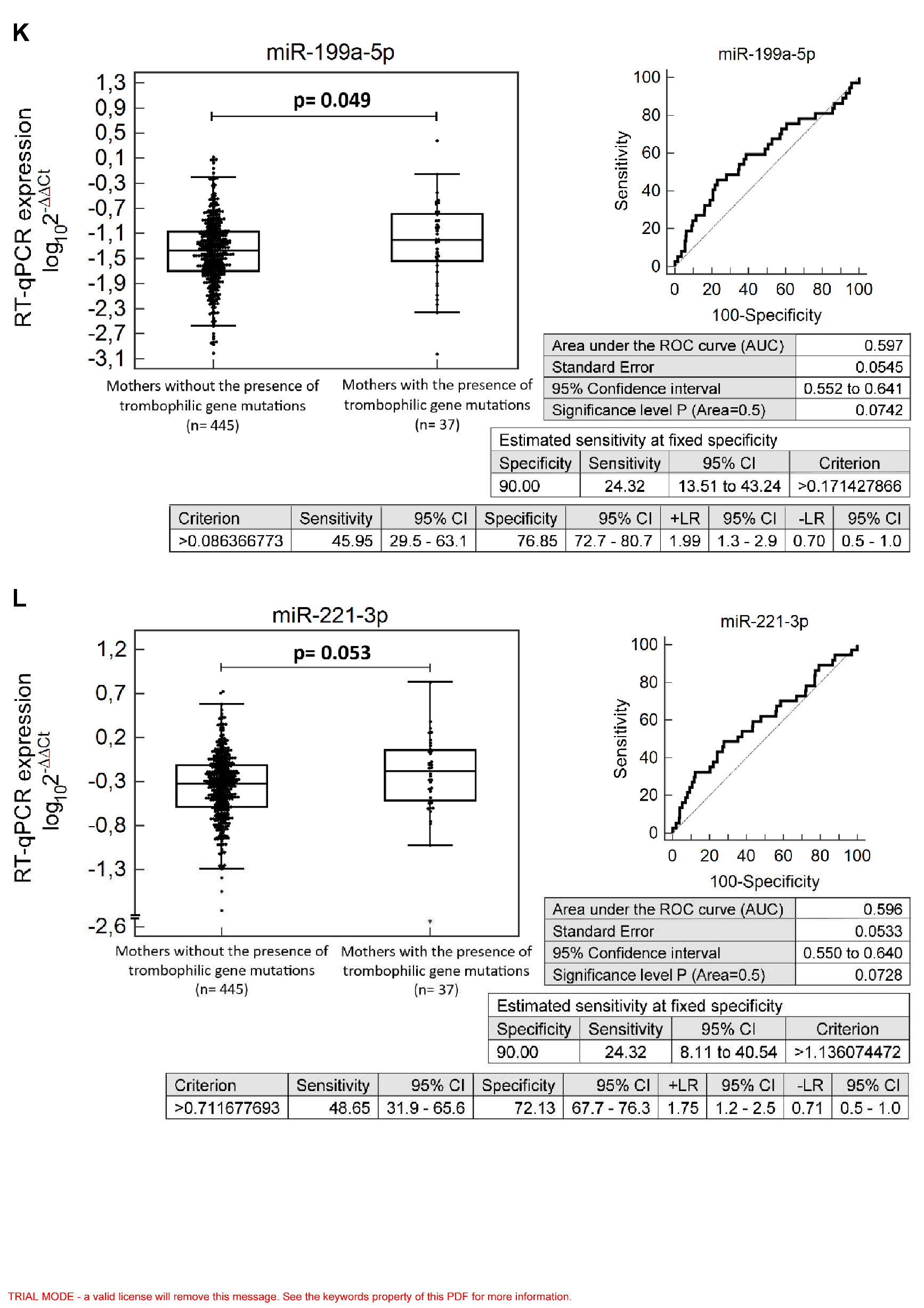
**

**
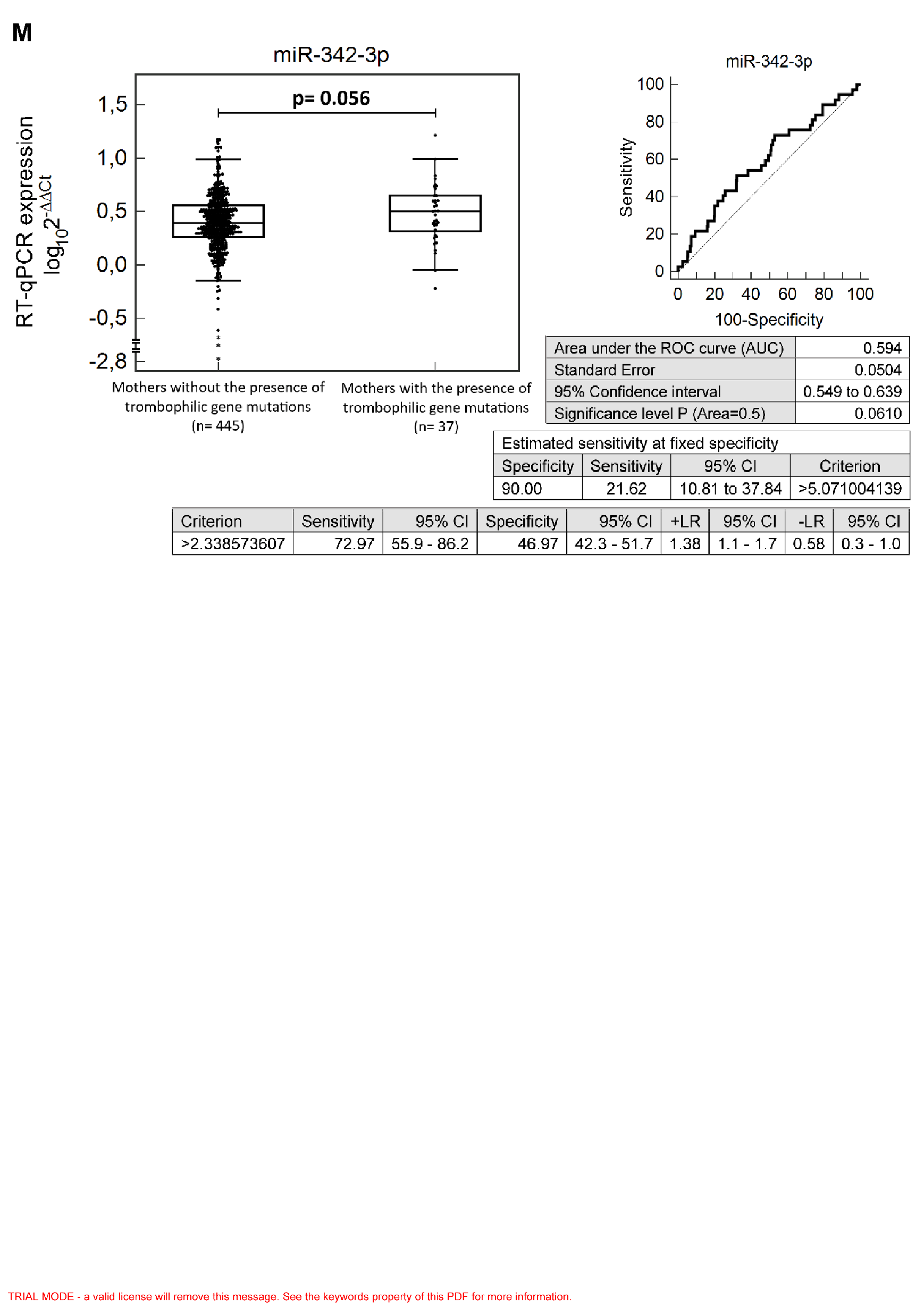
**

**Figure S9:** Aberrant microRNA expression profile in mothers with the presence of trombophilic gene mutations. Irrespective of the course of previous pregnancies (normal and complicated pregnancies altogether), at 10.0% FPR a substantial proportion of mothers showed up-regulation of miR-1-3p, miR-16-5p, miR-20b-5p, miR-24-3p, miR-26a-5p, miR-103a-3p, miR-125b-5p, miR-130b-3p, miR-143-3p, miR-146a-5p, miR-199a-5p, miR-221-3p, and miR-342-3p.
