# Supplementary material for: Association Analysis in Young and Middle-Aged Mothers—Relation between Expression of Cardiovascular Disease Associated MicroRNAs and Abnormal Clinical Findings
Source: J Pers Med. 2021 Jan 11;11(1):39. doi: 10.3390/jpm11010039 (PMC7826744; doi:10.3390/jpm11010039)
Supplement: Supplementary file 1 [file jpm-11-00039-s001.zip › Supplementary Material/Supplementary Methods.docx]

Materials and Methods

1.1. Participants

Normal pregnancies were characterised as birth of healthy infants with the weight > 2500 g after 37 completed weeks of gestation with the absence of medical, obstetrical, or surgical complications.

Gestational diabetes mellitus, glucose intolerance with the first onset during gestation, was diagnosed following the guidelines of The International Association of Diabetes and Pregnancy Study Groups (IADPSG) [1].

Gestational hypertension was diagnosed when hypertension (>140/90 mmHg in two determinations 4 h apart) without the sign of proteinuria appeared firstly after the twentieth week of gestation [2].

Preeclampsia was characterized as hypertension associated with proteinuria (>300 mg/24 h) with the onset after 20 weeks of gestation [2]. Severe preeclampsia was defined by the presence of one or more of the following symptoms: 1) a systolic blood pressure over 160 mmHg or a diastolic blood pressure over 110 mmHg, 2) proteinuria (>5 g of protein in a 24-h sample), 3) very low urine output (<500 mL in 24 h), 4) signs of pulmonary oedema or cyanosis, 5) impairment of liver function, 6) signs of severe headache, visual disturbances, 7) pain in the epigastric area or right upper quadrant, 8) thrombocytopenia, and 9) the presence of severe FGR [2].

FGR was defined as the estimated fetal weight (EFW) below the third percentile or below the 10^th^ percentile for the evaluated gestational age. The adjustments for the appropriate population standards were performed (the Hadlock formula, Astraia Software GmbH). Early FGR (the onset before 32 week of gestation) was diagnosed when the EFW was less than the third percentile or the presence of absent and/or zero diastolic flow was found in the umbilical artery. In addition, early FGR was classified when fetal weight below the 10th percentile was associated with an abnormal pulsatility index in the umbilical artery (>95th percentile) or an abnormal pulsatility index in the uterine artery (>95th percentile). Late FGR (onset after 32 week of gestation) was diagnosed when only one parameter (EWF below the third percentile) or the combination of 2 parameters were present: EFW below the tenth percentile and the cerebro–placental ratio (CPR) below the fifth percentile. CPR was expressed as a ratio between the middle cerebral artery and the umbilical artery pulsatility indexes [3-5].

PTB was defined as the occurrence of regular uterine contractions at a minimum frequency of two contractions per 10 min, along with cervical changes, leading to delivery before the 37th week of gestation. PPROM was defined as amniotic fluid leakage preceding the onset of labour by at least 2 h [6-8]. The exclusion criteria for preterm birth (PTB or PPROM) included gestational hypertension, preeclampsia, diabetes mellitus, significant vaginal bleeding, and signs of fetal growth restriction.

Altogether, women giving birth to children with inborn defects, chromosomal abnormalities, as well as women with pregnancies complicated with other complications were not included into the study.

*1.2. Blood pressure, BMI and waist circumference measurements*

Standardized BP, BMI and waist circumference measurements were performed as previously described [9, 10].

In brief, standardized blood pressure measurements were performed following New AHA Recommendations for Blood Pressure Measurement [11]. Blood pressure was measured 3 times in the right arm after a 5-minute rest period during which the participant sits using an automated device (OMRON M6W, Omron Healthcare Co., Kyoto, Japan). The average of the last 2 systolic and diastolic pressures was used for the data analyses.

Body weight was measured to the nearest 0.05 - 0.1 kg using an electronic scale and height was measured to the nearest 0.1 cm using a built-in stadiometer (calibrated balance scales, RADWAG WPT 100/200 OW, RADWAG, Czech Republic). BMI was calculated as weight divided by height squared. Waist circumference was measured to the nearest 0.1 cm using a measuring tape. Each measurement was taking twice. Regardless of the height or build, for most adults a waist measurement of greater than 80 cm for women is an indicator of the level of internal fat deposits which coat the heart, kidneys, liver, digestive organs and pancreas. This can increase the risk of heart disease and stroke.

1.3. Biological sampling and data collection

For detailed information please see our previous publications [9, 10]. Fasting blood samples (from 11 hours or more) were collected at the time of the study visit. Total serum cholesterol levels, serum high-density lipoprotein (HDL) cholesterol levels, serum low-density lipoprotein (LDL) cholesterol levels, serum triglycerides levels, serum lipoprotein A Lp(a) levels, serum high-sensitivity C-reactive protein (CRP) levels, plasma homocysteine levels, and serum uric acid levels were analysed using standard laboratory methods at the Institute for the Care of the Mother and Child. Information on a history of infertility treatment, the presence of trombophilic gene mutations, actual hormonal contraceptive use and actual smoking status were acquired from patients´ records deposited in hospital information system and during the time of the patients´ visit.

1.4. Processing of samples, reverse transcription, and relative quantification of microRNAs

Samples of unclotted whole peripheral venous blood (200 µl) were processed as previously described [12, 13]. Briefly, homogenized cell lysates were prepared as soon as possible after blood collection using QIAamp RNA Blood Mini Kit (Qiagen, Hilden, Germany, no: 52304).

Afterwards total RNA was extracted using a mirVana microRNA Isolation kit (Ambion, Austin, TX, USA, no: AM1560). The isolated RNA was treated with DNase I (Thermo Fisher Scientific, Carlsbad, CA, USA, no: EN0521).

Particular microRNAs were transcribed into cDNA using microRNA-specific stem-loop RT primers, components of TaqMan MicroRNA Assays, and TaqMan MicroRNA Reverse Transcription Kit (Applied Biosystems, Branchburg, NJ, USA, no: 4366597) as previously described [12, 13]. A total reaction volume of the reaction was 10 µL. Reverse transcriptase reaction was performed following the guidelines for a 7500 Real-Time PCR system (Applied Biosystems, Branchburg, NJ, USA): 30 minutes at 16 °C, 30 minutes at 42 °C, 5 minutes at 85 °C, and then held at 4 °C.

Relative quantification of microRNAs by real-time PCR was performed as previously described [12, 13]. cDNA (3 µL) was mixed with specific TaqMan MGB primers and probes (TaqMan MicroRNA Assay, Applied Biosystems, Branchburg, NJ, USA), and the constituents of the TaqMan Universal PCR Master Mix (Applied Biosystems, Branchburg, NJ, USA, no: 4318157) in a total reaction volume of 15 µL. The samples were regarded as positive if Ct (threshold cycle) was below 40 (Ct <40).

The comparative Ct method was used to determine the expression of each microRNA [14]. The expression of studied microRNAs was normalized to geometric mean of RNU58A and RNU38B, endogenous controls with the lowest expression variability between studied samples [15]. A reference sample was used throughout the study for relative quantification. As a reference sample we used small RNAs enriched RNA fraction extracted from the fetal part of one randomly selected placenta of normally ongoing pregnancy.

1.5. Statistical analysis

The data normality was assessed using the Shapiro-Wilk test [16]. Since the experimental data did not show a normal distribution, microRNA levels were compared among the groups of children using the Mann-Whitney test.

Receivers operating characteristic (ROC) curves were used to assess the areas under the curves (AUC), the optimal cut-off points, and the respective sensitivities at 10.0% false positive rate (FPR) for particular microRNAs (MedCalc Software bvba, Ostend, Belgium). The significance level was established at a *p-*value of *p* < 0.05.

To identify the optimal combinations of microRNA biomarkers logistic regression combined with ROC curve analysis was used (MedCalc Software bvba, Ostend, Belgium). In brief, in this setting, the power of the model's predicted values to discriminate between positive and negative cases is quantified by the area under the ROC curve. To perform a full ROC curve analysis the predicted probabilities are first saved and next used as a new variable in [ROC curve analysis](mk:@MSITStore:C:\Users\ILONAH~2\AppData\Local\Temp\MedCalc\medcalc.chm::/roc-curves.htm). The dependent variable used in logistic regression then acts as the classification variable in the ROC curve analysis dialog box.

Statistica software (version 9.0; StatSoft, Inc., Tulsa, OK, USA) was used to generate box plots of log-normalized gene expression values (RT-qPCR expression, log_10_ 2^-ΔΔCt^) for particular microRNAs. The box plots display the medians, the 75^th^ and 25^th^ percentiles (the upper and lower limits of the boxes), the maximum and minimum values (the upper and lower whiskers), outliers (circles), and extremes (asterisks). Dot plots, all observations are also displayed in the charts.

References:

1. International Association of Diabetes and Pregnancy Study Groups Consensus Panel; Metzger, B.E.; Gabbe, S.G.; Persson, B.; Buchanan, T.A.; Catalano, P.A.; Damm, P.; Dyer, A.R.; Leiva, A.D.; Hod, M.; Kitzmiler, J.L.; Lowe, L.P.; McIntyre, H.D.; Oats, J.J.; Omori, Y.; Schmidt, M.I. International association of diabetes and pregnancy study groups recommendations on the diagnosis and classification of hyperglycemia in pregnancy. *Diabetes Care* **2010**, *33*, 676-682.
2. ACOG practice bulletin. Diagnosis and management of preeclampsia and eclampsia. *Obstet Gynecol* **2002**, *99*, 159–167.
3. [Figueras, F](https://www.ncbi.nlm.nih.gov/pubmed/?term=Figueras%20F%5BAuthor%5D&cauthor=true&cauthor_uid=24839087).; [Gratacos; E](https://www.ncbi.nlm.nih.gov/pubmed/?term=Gratacos%20E%5BAuthor%5D&cauthor=true&cauthor_uid=24839087). Stage-based approach to the management of fetal growth restriction. [*Prenat* *Diagn*](https://www.ncbi.nlm.nih.gov/pubmed/24839087) **2014**, *34*, 655-659.
4. [Baschat, A.A](https://www.ncbi.nlm.nih.gov/pubmed/?term=Baschat%20AA%5BAuthor%5D&cauthor=true&cauthor_uid=21520312). Neurodevelopment following fetal growth restriction and its relationship with antepartum parameters of placental dysfunction. [*Ultrasound Obstet Gynecol*](https://www.ncbi.nlm.nih.gov/pubmed/21520312) **2011**, *37*, 501-514.
5. [Nardozza, L.M](https://www.ncbi.nlm.nih.gov/pubmed/?term=Nardozza%20LM%5BAuthor%5D&cauthor=true&cauthor_uid=28285426).; [Caetano, A.C](https://www.ncbi.nlm.nih.gov/pubmed/?term=Caetano%20AC%5BAuthor%5D&cauthor=true&cauthor_uid=28285426).; [Zamarian, A.C](https://www.ncbi.nlm.nih.gov/pubmed/?term=Zamarian%20AC%5BAuthor%5D&cauthor=true&cauthor_uid=28285426).; [Mazzola, J.B](https://www.ncbi.nlm.nih.gov/pubmed/?term=Mazzola%20JB%5BAuthor%5D&cauthor=true&cauthor_uid=28285426).; [Silva, C.P](https://www.ncbi.nlm.nih.gov/pubmed/?term=Silva%20CP%5BAuthor%5D&cauthor=true&cauthor_uid=28285426).; [Marçal, V.M](https://www.ncbi.nlm.nih.gov/pubmed/?term=Mar%C3%A7al%20VM%5BAuthor%5D&cauthor=true&cauthor_uid=28285426).; [Lobo, T.F](https://www.ncbi.nlm.nih.gov/pubmed/?term=Lobo%20TF%5BAuthor%5D&cauthor=true&cauthor_uid=28285426).; [Peixoto, A.B](https://www.ncbi.nlm.nih.gov/pubmed/?term=Peixoto%20AB%5BAuthor%5D&cauthor=true&cauthor_uid=28285426).; [Araujo Júnior, E](https://www.ncbi.nlm.nih.gov/pubmed/?term=Araujo%20J%C3%BAnior%20E%5BAuthor%5D&cauthor=true&cauthor_uid=28285426). Fetal growth restriction: current knowledge. [*Arch Gynecol Obstet*](https://www.ncbi.nlm.nih.gov/pubmed/28285426) **2017**, *295*, 1061-1077.
6. Goldenberg, R.L.; Culhane, J.F.; Iams, J.D.; Romero, R. Epidemiology and causes of preterm birth. *Lancet* **2008**, *371*, 75-84.
7. Moutquin, J.M.; Milot Roy V.; Irion O. Preterm prevention: effectivenss of current strategies. *J Soc Obstet Gynaecol Can* **1996**, *18*, 571-588.
8. [Romero, R](http://www.ncbi.nlm.nih.gov/pubmed/?term=Romero%20R%5BAuthor%5D&cauthor=true&cauthor_uid=17206962).; [Espinoza, J](http://www.ncbi.nlm.nih.gov/pubmed/?term=Espinoza%20J%5BAuthor%5D&cauthor=true&cauthor_uid=17206962).; [Kusanovic, J.P](http://www.ncbi.nlm.nih.gov/pubmed/?term=Kusanovic%20JP%5BAuthor%5D&cauthor=true&cauthor_uid=17206962).; [Gotsch, F](http://www.ncbi.nlm.nih.gov/pubmed/?term=Gotsch%20F%5BAuthor%5D&cauthor=true&cauthor_uid=17206962).; [Hassan, S](http://www.ncbi.nlm.nih.gov/pubmed/?term=Hassan%20S%5BAuthor%5D&cauthor=true&cauthor_uid=17206962).; [Erez, O](http://www.ncbi.nlm.nih.gov/pubmed/?term=Erez%20O%5BAuthor%5D&cauthor=true&cauthor_uid=17206962).; [Chaiworapongsa, T](http://www.ncbi.nlm.nih.gov/pubmed/?term=Chaiworapongsa%20T%5BAuthor%5D&cauthor=true&cauthor_uid=17206962)., [Mazor, M](http://www.ncbi.nlm.nih.gov/pubmed/?term=Mazor%20M%5BAuthor%5D&cauthor=true&cauthor_uid=17206962). The preterm parturition syndrome. *BJOG* **2006**, *113*, 17-42.
9. Hromadnikova, I.; Kotlabova, K.; Dvorakova, L.; Krofta, L. [Maternal Cardiovascular Risk Assessment 3-to-11 Years Postpartum in Relation to Previous Occurrence of Pregnancy-Related Complications.](https://www.ncbi.nlm.nih.gov/pubmed/31010048) *J Clin Med* **2019**, *8*, pii: E544.
10. Hromadnikova, I.; Kotlabova, K.; Dvorakova, L.; Krofta, L. Evaluation of Vascular Endothelial Function in Young and Middle-Aged Women with Respect to a History of Pregnancy, Pregnancy-Related Complications, Classical Cardiovascular Risk Factors, and Epigenetics. *Int J Mol Sci* **2020**, *21*, pii: E430.
11. Practice Guidelines: New AHA Recommendations for Blood Pressure Measurement. *Am Fam Physician* **2005**, *72*, 1391–1398.
12. Hromadnikova. I.; Kotlabova, K.; Dvorakova, L.; Krofta, L. [Postpartum profiling of microRNAs involved in pathogenesis of cardiovascular/cerebrovascular diseases in women exposed to pregnancy-related complications.](https://www.ncbi.nlm.nih.gov/pubmed/31151766) *Int J Cardiol* **2019**, *291*, 158-167.
13. Hromadnikova, I.; Kotlabova, K.; Dvorakova, L.; Krofta, L. Diabetes Mellitus and Cardiovascular Risk Assessment in Mothers with a History of Gestational Diabetes Mellitus Based on Postpartal Expression Profile of MicroRNAs Associated with Diabetes Mellitus and Cardiovascular and Cerebrovascular Diseases. *Int J Mol Sci* **2020**, *21*, 2437.
14. Livak, K.J.; Schmittgen, T.D. Analysis of relative gene expression data using real-time quantitative PCR and the 2(-Delta Delta C(T)) Method. *Methods* **2001**, 25, 402–408.
15. Vandesompele, J.; de Preter, K.; Pattyn, F.; Poppe, B.; Van Roy, N.; de Paepe, A.; Speleman, F. Accurate normalization of real-time quantitative RT-PCR data by geometric averaging of multiple internal control genes. *Genome Biol* **2002**, *3*, research0034.
16. Shapiro, S.S.; Wilk, M.B. An Analysis of Variance Test for Normality (Complete Samples) *Biometrika* **1965**, *52*, 591–611.
