# Supplementary material for: Association Analysis in Young and Middle-Aged Mothers—Relation between Expression of Cardiovascular Disease Associated MicroRNAs and Abnormal Clinical Findings
Source: J Pers Med. 2021 Jan 11;11(1):39. doi: 10.3390/jpm11010039 (PMC7826744; doi:10.3390/jpm11010039)
Supplement: Supplementary file 1 [file jpm-11-00039-s001.zip › Supplementary Material/Supplementary Table 1.docx]

**Supplementary Table S1.**

**Table S1.** The role of selected microRNAs in the pathogenesis of diabetes mellitus and cardiovascular/cerebrovascular diseases

| **miRBase ID** | **Gene location on chromosome** | **Role in the pathogenesis of diabetes mellitus and cardiovascular/cerebrovascular diseases** |
| --- | --- | --- |
| hsa-miR-1-3p | 20q13.3 [1]  18q11.2 | Acute myocardial infarction, heart ischemia, post-myocardial infarction complications [2], thoracic aortic aneurysm [3], diabetes mellitus [4, 5], vascular endothelial dysfunction [6] |
| hsa-miR-16-5p | 13q14.2 | Myocardial infarction [7, 8], heart failure [9], acute coronary syndrome, cerebral ischaemic events [10], gestational diabetes mellitus [11-13], diabetes mellitus [14-16] |
| hsa-miR-17-5p | 13q31.3 [17, 18] | Cardiac development [19], ischemia/reperfusion-induced cardiac injury [20], kidney ischemia-reperfusion injury [21], diffuse myocardial fibrosis in hypertrophic cardiomyopathy [22], acute ischemic stroke [23], coronary artery disease [24], adipogenic differentiation [25], gestational diabetes mellitus [11, 12], diabetes mellitus [16 ,26] |
| hsa-miR-20a-5p | 13q31.3 [27] | Pulmonary hypertension [28], gestational diabetes mellitus [11, 12, 29], diabetic retinopathy [30], diabetes with abdominal aortic aneurysm [31] |
| hsa-miR-20b-5p | Xq26.2 [27] | Hypertension-induced heart failure [32], insulin resistance [33], T2DM [34, 35], diabetic retinopathy [36] |
| hsa-miR-21-5p | 17q23.2 [37] | Homeostasis of the cardiovascular system [38], cardiac fibrosis and heart failure [39, 40], thoracic aortic aneurysm [3], ascending aortic aneurysm [41], regulation of hypertension-related genes [42], myocardial infarction [43], insulin resistance [33], T2DM [44], T2DM with major cardiovascular events [45], T1DM [46-48], diabetic nephropathy [49] |
| hsa-miR-23a-3p | 19p13.12 | Heart failure [50], coronary artery disease [51], cerebral ischemia-reperfusion [52], vascular endothelial dysfunction [6], small and large abdominal aortic aneurysm [53], obesity and insulin resistance [54] |
| hsa-miR-24-3p | 19p13.12 | Asymptomatic carotid stenosis [55], familial hypercholesterolemia and coronary artery disease [56], angina pectoris [57], ischemic dilated cardiomyopathy [58], small and large abdominal aortic aneurysm [53], myocardial ischemia/reperfusion [59, 60], diabetes mellitus [5, 16, 20, 22] |
| hsa-miR-26a-5p | 3p22.2 [61]  12q14.1 | Heart failure, cardiac hypertrophy [62], myocardial infarction [43, 63, 64], ischemia/reperfusion injury [65], pulmonary arterial hypertension [66], T1DM [67], diabetic nephropathy [49] |
| hsa-miR-29a-3p | 7q32.3 | Ischemia/reperfusion-induced cardiac injury [68], cardiac cachexia, heart failure [69], atrial fibrillation [70], diffuse myocardial fibrosis in hypertrophic cardiomyopathy [22], coronary artery disease [71], pulmonary arterial hypertension [66], gestational diabetes mellitus [72], diabetes mellitus [4, 15, 73, 74] |
| hsa-miR-92a-3p | 13q31.3  Xq26.2 | Mitral chordae tendineae rupture [75], children with rheumatic carditis [76], myocardial infarction [77], heart failure [78], coronary artery disease [79], renal injury – associated atherosclerosis [80] |
| hsa-miR-100-5p | 11q24.1 | Failing human heart, idiopathic dilated cardiomyopathy, ischemic cardiomyopathy [58], regulation of hypertension-related genes [42], T1DM [46] |
| hsa-miR-103a-3p | 5q34 [81]  20p13 | Hypertension [82], hypoxia-induced pulmonary hypertension [83], myocardial ischemia/reperfusion injury, acute myocardial infarction [84], ischemic dilated cardiomyopathy [58], obesity, regulation of insulin sensitivity [85], T1DM [86] |
| hsa-miR-125b-5p | 11q24.1 [86]  21q21.1 | Acute ischemic stroke [87], acute myocardial infarction [88, 89], ischemic dilated cardiomyopathy [58], ascending aortic aneurysm [41], gestational diabetes mellitus [90], T1DM [91, 92], T2DM [93] |
| hsa-miR-126-3p | 9q34.3 [94] | Acute myocardial infarction [64], thoracic aortic aneurysm [3], T2DM [45, 95], T2DM with major cardiovascular events [45], gestational diabetes mellitus [96] |
| hsa-miR-130b-3p | 22q11.21 | Hypertriglyceridemia [97, 98], intracranial aneurysms [99], hyperacute cerebral infarction [100], T2DM [44, 101, 102], gestational diabetes mellitus [96] |
| hsa-miR-133a-3p | 18q11.2 [103]  20q13.33 | Heart failure [104], myocardial fibrosis in hypertrophic cardiomyopathy [22, 105], arrhythmogenesis in the hypertrophic and failing hearts [106, 107], coronary artery calcification [108], thoracic aortic aneurysm [3], ascending aortic aneurysm [41], diabetes mellitus [4, 5] |
| hsa-miR-143-3p | 5q33 | Intracranial aneurysms [109], coronary heart disease [110], myocardial infarction [111], myocardial hypertrophy [112], dilated cardiomyopathy [113], pulmonary arterial hypertension [114], acute ischemic stroke [87], ascending aortic aneurysm [41] |
| hsa-miR-145-5p | 5q33 | Hypertension [115, 116], dilated cardiomyopathy [117], myocardial infarction [118, 119], stroke [119], acute cerebral ischemic/reperfusion [120], T2DM [61, 121], T1DM [44], diabetic retinopathy [122], gestational diabetes mellitus [123] |
| hsa-miR-146a-5p | 5q33.3 [124, 125] | Angiogenesis [126], hypoxia, ischemia/reperfusion-induced cardiac injury [127], myocardial infarction [8], coronary atherosclerosis, coronary heart disease in patients with subclinical hypothyroidism [128], thoracic aortic aneurysm [3], acute ischemic stroke, acute cerebral ischemia [129], T2DM [16, 44], T1DM [67], diabetic nephropathy [49] |
| hsa-miR-155-5p | 21q21.3 | Thoracic aortic aneurysm [3], type 1 diabetes [85], gestational diabetes mellitus [13], adolescent obesity [130], diet-induced obesity and obesity resistance [131], atherosclerosis [132], hyperlipidemia – associated endotoxemia [133], coronary plaque rupture [134], children with cyanotic heart disease [135], chronic kidney disease and nocturnal hypertension [136], atrial fibrillation [137] |
| hsa-miR-181a-5p | 1q32.1 [138]  9q33.3 | Regulation of hypertension-related genes [23], atherosclerosis [138], metabolic syndrome, coronary artery disease [139], non-alcoholic fatty liver disease [140], ischaemic stroke, transient ischaemic attack, acute myocardial infarction [141, 142], obesity and insulin resistance [54, 138, 139], T1DM [44, 143], T2DM [138, 142] |
| hsa-miR-195-5p | 17p13.1 [144] | Cardiac hypertrophy, heart failure [145, 146], abdominal aortic aneurysms [147], aortic stenosis [148], T2DM [121], gestational diabetes mellitus [149] |
| hsa-miR-199a-5p | 1q24.3  19p13.2 | T1DM, T2DM, gestational diabetes mellitus [150], diabetic retinopathy [151], cerebral ischemic injury [152], heart failure [153], hypertension [154, 155], congenital heart disease [156], pulmonary artery hypertension [157], unstable angina [158], hypoxia in myocardium [156], acute kidney injury [159] |
| hsa-miR-210-3p | 11p15.5 | Cardiac hypertrophy [160], acute kidney injury [161], myocardial infarction [162], atherosclerosis [163] |
| hsa-miR-221-3p | Xp11.3 | Asymptomatic carotid stenosis [55], cardiac amyloidosis [164], heart failure [165], atherosclerosis [166, 167], aortic stenosis [168], acute myocardial infarction [169], acute ischemic stroke [170], focal cerebral ischemia [171], pulmonary artery hypertension [172], obesity [173] |
| hsa-miR-342-3p | 14q32.2 | Cardiac amyloidosis [164], obesity [174], T1DM [44, 150, 175], T2DM [150, 176, 177], gestational diabetes mellitus [150], endothelial dysfunction [178] |
| hsa-miR-499a-5p | 20q11.22 | Myocardial infarction [8, 179], hypoxia [180], cardiac regeneration [181], vascular endothelial dysfunction [6] |
| hsa-miR-574-3p | 4p14 | Myocardial infarction [182], coronary artery disease [98], cardiac amyloidosis [164], stroke [183], T2DM [102, 184] |

T1DM, diabetes mellitus type 1; T2DM, diabetes mellitus type 2.

References:

1. Li, J.; Dong, X.; Wang, Z.; Wu, J. MicroRNA-1 in Cardiac Diseases and Cancers. *Korean J Physiol Pharmacol* **2014**, *18*, 359-363.
2. Li, Y.Q.; Zhang, M.F.; Wen, H.Y.; Hu, C.L.; Liu, R.; Wei, H.Y.; Ai, C.M.; Wang, G.; Liao, X.X.; Li, X. Comparing the diagnostic values of circulating microRNAs and cardiac troponin T in patients with acute myocardial infarction. *Clinics (Sao Paulo)* **2013**, *68*, 75-80.
3. Gasiulė, S.; Stankevičius, V.; Patamsytė, V.; Ražanskas, R.; Zukovas, G.; Kapustina, Z.; Zaliaduonytė, D.; Benetis, R.; Lesauskaitė, V.; Vilkaitis, G. Tissue-Specific miRNAs Regulate the Development of Thoracic Aortic Aneurysm: The Emerging Role of KLF4 Network. *J Clin Med* **2019**, *8*, 1609.
4. Gerlinger-Romero, F.; Yonamine, C.Y.; Junior, D.C.; Esteves, J.V.; Machado, U.F. Dysregulation between TRIM63/FBXO32 expression and soleus muscle wasting in diabetic rats: potential role of miR-1-3p, -29a/b-3p, and -133a/b-3p. *Mol Cell Biochem* **2017**, *427*, 187-199.
5. Kokkinopoulou, I.; Maratou, E.; Mitrou, P.; Boutati, E.; Sideris, D.C.; Fragoulis, E.G.; Christodoulou, M.I. Decreased expression of microRNAs targeting type-2 diabetes susceptibility genes in peripheral blood of patients and predisposed individuals. *Endocrine* **2019**, *66*, 226-239.
6. Hromadnikova, I.; Kotlabova, K.; Dvorakova, L.; Krofta, L. Evaluation of Vascular Endothelial Function in Young and Middle-Aged Women with Respect to a History of Pregnancy, Pregnancy-Related Complications, Classical Cardiovascular Risk Factors, and Epigenetics. *Int J Mol Sci* **2020**, *21*, pii: E430.
7. Wang, X.; Shang, Y.; Dai, S.; Wu, W.; Yi, F.; Cheng, L. MicroRNA-16-5p aggravates myocardial infarction injury by targeting expression of insulin receptor substrates 1 and mediating myocardial apoptosis and angiogenesis. *Curr Neurovasc Res* **2019**, [Epub ahead of print].
8. O´Sullivan J.F.; Neylon, A.; McGorrian, C.; Blake, G.J. miRNA-93-5p and other miRNAs as predictors of coronary artery disease and STEMI. *Int J Cardiol* **2016**, *224*, 310-316.
9. Vegter, E.L.; Schmitter, D.; Hagemeijer, Y.; Ovchinnikova, E.S.; van der Harst, P.; Teerlink, J.R.; O'Connor, C.M.; Metra, M.; Davison, B.A.; Bloomfield, D.; Cotter, G.; Cleland, J.G.; Givertz, M.M.; Ponikowski, P.; van Veldhuisen, D.J.; van der Meer, P.; Berezikov, E.; Voors, A.A.; Khan, M.A. Use of biomarkers to establish potential role and function of circulating microRNAs in acute heart failure. *Int J Cardiol* **2016**, *224*, 231-239.
10. Gacoń, J.; Badacz, R.; Stępień, E.; Karch, I.; Enguita, F.J.; Żmudka, K.; Przewłocki, T.; Kabłak-Ziembicka, A. Diagnostic and prognostic micro-RNAs in ischaemic stroke due to carotid artery stenosis and in acute coronary syndrome: a four-year prospective study. *Kardiol Pol* **2018**, *76*, 362-369.
11. Zhu, Y.; Tian, F.; Li, H.; Zhou, Y.; Lu, J.; Ge, Q. Profiling maternal plasma microRNA expression in early pregnancy to predict gestational diabetes mellitus. *Int J Gynaecol Obstet* **2015**, *130*, 49-53.
12. Cao, Y.L.; Jia, Y.J.; Xing, B.H.; Shi, D.D.; Dong, X.J. Plasma microRNA-16-5p, -17-5p and -20a-5p: Novel diagnostic biomarkers for gestational diabetes mellitus. *J Obstet Gynaecol Res* **2017**, *43*, 974-981.
13. Hocaoglu, M.; Demirer, S.; Senturk, H.; Turgut, A.; Komurcu-Bayrak, E. Differential expression of candidate circulating microRNAs in maternal blood leukocytes of the patients with preeclampsia and gestational diabetes mellitus. *Pregnancy Hypertens* **2019**, *17*, 5-11.
14. Duan, Y.R,; Chen, B.P.; Chen, F.; Yang, S.X.; Zhu, C.Y.; Ma, Y.L.; Li, Y.; Shi, J. Exosomal microRNA-16-5p from human urine-derived stem cells ameliorates diabetic nephropathy through protection of podocyte. *J Cell Mol Med* **2019**, [Epub ahead of print].
15. Assmann, T.S.; Recamonde-Mendoza, M.; Costa, A.R.; Puñales, M.; Tschiedel, B.; Canani, L.H.; Bauer, A.C.; Crispim, D. Circulating miRNAs in diabetic kidney disease: case-control study and in silico analyses. *Acta Diabetol* **2019**, *56*, 55-65.
16. Alicka, M.; Major, P.; Wysocki, M.; Marycz, K. Adipose-Derived Mesenchymal Stem Cells Isolated from Patients with Type 2 Diabetes Show Reduced "Stemness" through an Altered Secretome Profile, Impaired Anti-Oxidative Protection, and Mitochondrial Dynamics Deterioration. *J Clin Med* **2019**, *8*, pii: E765.
17. Mogilyansky, E.; Rigoutsos, I. The miR-17/92 cluster: a comprehensive update on its genomics, genetics, functions and increasingly important and numerous roles in health and disease. *Cell Death Differ* **2013**, *20*, 1603-1614.
18. Zhou, L.; Qi, R.Q.; Liu, M.; Xu, Y.P.; Li, G.; Weiland, M.; Kaplan, D.H.; Mi, Q.S. microRNA miR-17-92 cluster is highly expressed in epidermal Langerhans cells but not required for its development. *Genes Immun* **2014**, *15*, 57-61.
19. Danielson, L.S.; Park, D.S.; Rotllan, N.; Chamorro-Jorganes, A.; Guijarro, M.V.; Fernandez-Hernando, C.; Fishman, G.I.; Phoon, C.K.; Hernando, E. Cardiovascular dysregulation of miR-17-92 causes a lethal hypertrophic cardiomyopathy and arrhythmogenesis. *FASEB J* **2013**, *27*, 1460-1467.
20. Du, W.; Pan, Z.; Chen, X.; Wang, L.; Zhang, Y.; Li, S.; Liang, H.; Xu, C.; Zhang, Y.; Wu, Y.; Shan, H.; Lu, Y. By targeting Stat3 microRNA-17-5p promotes cardiomyocyte apoptosis in response to ischemia followed by reperfusion. *Cell Physiol Biochem* **2014**, *34*, 955-965.
21. Kaucsár, T.; Révész, C.; Godó, M.; Krenács, T.; Albert, M.; Szalay, C.I.; Rosivall, L.; Benyó, Z.; Bátkai, S.; Thum, T.; Szénási, G.; Hamar, P. Activation of the miR-17 family and miR-21 during murine kidney ischemia-reperfusion injury. *Nucleic Acid Ther* **2013**, *23*, 344-354.
22. Fang, L.; Ellims, A.H.; Moore, X.L.; White, D.A.; Taylor, A.J.; Chin-Dusting, J.; Dart, A.M. Circulating microRNAs as biomarkers for diffuse myocardial fibrosis in patients with hypertrophic cardiomyopathy. *J Transl Med* **2015**, *13*, 314.
23. Wu, J.; Du, K.; Lu, X. Elevated expressions of serum miR-15a, miR-16, and miR-17-5p are associated with acute ischemic stroke. *Int J Clin Exp Med* **2015**, *8*, 21071-21079.
24. Chen, J.; Xu, L.; Hu, Q.; Yang, S.; Zhang, B.; Jiang, H. MiR-17-5p as circulating biomarkers for the severity of coronary atherosclerosis in coronary artery disease. *Int J Cardiol* **2015**, *197*, 123-124.
25. Tian, L.; Song, Z.; Shao, W.; Du, W.W.; Zhao, L.R.; Zeng, K.; Yang, B.B.; Jin, T. Curcumin represses mouse 3T3-L1 cell adipogenic differentiation via inhibiting miR-17-5p and stimulating the Wnt signalling pathway effector Tcf7l2. *Cell Death Dis* **2017**, *8*, e2559.
26. Chen, T.C.; Sung, M.L.; Kuo, H.C.; Chien, S.J.; Yen, C.K.; Chen, C.N. Differential regulation of human aortic smooth muscle cell proliferation by monocyte-derived macrophages from diabetic patients. *PLoS One* **2014**, *9*, e113752.
27. Mendell, J.T. miRiad roles for the miR-17-92 cluster in development and disease. *Cell* **2008**, *133*, 217-222.
28. Brock, M.; Samillan, V.J.; Trenkmann, M.; Schwarzwald, C.; Ulrich, S.; Gay, R.E.; Gassmann, M.; Ostergaard, L.; Gay, S.; Speich, R.; Huber, L.C. AntagomiR directed against miR-20a restores functional BMPR2 signalling and prevents vascular remodelling in hypoxia-induced pulmonary hypertension. *Eur Heart J* **2014**, *35*, 3203-3211.
29. Pheiffer, C.; Dias, S.; Rheeder, P.; Adam, S. Decreased Expression of Circulating miR-20a-5p in South African Women with Gestational Diabetes Mellitus. *Mol Diagn Ther* **2018**, *22*, 345-352.
30. Platania, C.B.M.; Maisto, R.; Trotta, M.C.; D'Amico, M.; Rossi, S.; Gesualdo, C.; D'Amico, G.; Balta, C.; Herman, H.; Hermenean, A.; Ferraraccio, F.; Panarese, I.; Drago, F.; Bucolo, C. Retinal and circulating miRNA expression patterns in diabetic retinopathy: An in silico and in vivo approach. *Br J Pharmacol* **2019**, *176*, 2179-2194.
31. Lareyre, F.; Clément, M.; Moratal, C.; Loyer, X.; Jean-Baptiste, E.; Hassen-Khodja, R.; Chinetti, G.; Mallat, Z.; Raffort, J. Differential micro-RNA expression in diabetic patients with abdominal aortic aneurysm. *Biochimie* **2019**, *162*, 1-7.
32. Dickinson, B.A.; Semus, H.M.; Montgomery, R.L.; Stack, C.; Latimer, P.A.; Lewton, S.M.; Lynch, J.M.; Hullinger, T.G.; Seto, A.G.; van Rooij, E. Plasma microRNAs serve as biomarkers of therapeutic efficacy and disease progression in hypertension-induced heart failure. *Eur J Heart Fail* **2013**, *15*, 650-659.
33. Flowers, E.; Aouizerat, B.E.; Abbasi, F.; Lamendola, C.; Grove, K.M.; Fukuoka, Y.; Reaven, G.M. Circulating microRNA-320a and microRNA-486 predict thiazolidinedione response: Moving towards precision health for diabetes prevention. *Metabolism* **2015**, *64*, 1051-1059.
34. Katayama, M.; Wiklander, O.P.B.; Fritz, T.; Caidahl, K.; El-Andaloussi, S.; Zierath, J.R.; Krook, A. Circulating Exosomal miR-20b-5p Is Elevated in Type 2 Diabetes and Could Impair Insulin Action in Human Skeletal Muscle. *Diabetes* **2019**, *68*, 515-526.
35. Xiong, Y.; Chen, L.; Yan, C.; Zhou, W.; Endo, Y.; Liu, J.; Hu, L.; Hu, Y.; Mi, B.; Liu, G. Circulating Exosomal miR-20b-5p Inhibition Restores Wnt9b Signaling and Reverses Diabetes-Associated Impaired Wound Healing. *Small* **2020**, *16*, e1904044.
36. Zhu, K.; Hu, X.; Chen, H.; Li, F.; Yin, N.; Liu, A.L.; Shan, K.; Qin, Y.W.; Huang, X.; Chang, Q.; Xu, G.Z.; Wang, Z. Downregulation of circRNA DMNT3B contributes to diabetic retinal vascular dysfunction through targeting miR-20b-5p and BAMBI. *EBioMedicine* **2019**, *49*, 341-353.
37. Sekar, D.; Venugopal, B.; Sekar, P.; Ramalingam, K. Role of microRNA 21 in diabetes and associated/related diseases. *Gene* **2016**, *582*, 14-18.
38. Suárez, Y.; Fernández-Hernando, C.; Pober, J.S.; Sessa, W.C. Dicer dependent microRNAs regulate gene expression and functions in human endothelial cells. *Circ Res* **2007**, *100*, 1164-1173.
39. Dong, S.; Ma, W.; Hao, B.; Hu, F.; Yan, L.; Yan, X.; Wang, Y.; Chen, Z.; Wang, Z. microRNA-21 promotes cardiac fibrosis and development of heart failure with preserved left ventricular ejection fraction by up-regulating Bcl-2. *Int J Clin Exp Pathol* **2014**, *7*, 565-574.
40. Zhang, J.; Xing, Q.; Zhou, X.; Li, J.; Li, Y.; Zhang, L.; Zhou, Q.; Tang, B. Circulating miRNA 21 is a promising biomarker for heart failure. *Mol Med Rep* **2017**, *16*, 7766-7774.
41. Licholai, S.; Blaż, M.; Kapelak, B.; Sanak, M. Unbiased Profile of MicroRNA Expression in Ascending Aortic Aneurysm Tissue Appoints Molecular Pathways Contributing to the Pathology. *Ann Thorac Surg* **2016**, *102*, 1245-1252.
42. Kriegel, A.J.; Baker, M.A.; Liu, Y.; Liu, P.; Cowley, A.W. Jr.; Liang, M. Endogenous microRNAs in human microvascular endothelial cells regulate mRNAs encoded by hypertension-related genes. *Hypertension* **2015**, *66*, 793-799.
43. Velle-Forbord, T.; Eidlaug, M.; Debik, J.; Sæther, J.C.; Follestad, T.; Nauman, J.; Gigante, B.; Røsjø, H.; Omland, T.; Langaas, M.; Bye, A. Circulating microRNAs as predictive biomarkers of myocardial infarction: Evidence from the HUNT study. *Atherosclerosis* **2019**, *289*, 1-7.
44. Demirsoy, İ.H.; Ertural, D.Y.; Balci, Ş.; Çınkır, Ü.; Sezer, K.; Tamer, L.; Aras, N. Profiles of Circulating MiRNAs Following Metformin Treatment in Patients with Type 2 Diabetes. *J Med Biochem* **2018**, *37*, 499-506.
45. Olivieri, F.; Spazzafumo, L.; Bonafè, M.; Recchioni, R.; Prattichizzo, F.; Marcheselli, F.; Micolucci, L.; Mensà, E.; Giuliani, A.; Santini, G.; Gobbi, M.; Lazzarini, R.; Boemi, M.; Testa, R.; Antonicelli, R.; Procopio, A.D.; Bonfigli, A.R. MiR-21-5p and miR-126a-3p levels in plasma and circulating angiogenic cells: relationship with type 2 diabetes complications. *Oncotarget* **2015**, *6*, 35372-35382.
46. Assmann, T.S.; Recamonde-Mendoza, M.; De Souza, B.M.; Crispim, D. MicroRNA expression profiles and type 1 diabetes mellitus: systematic review and bioinformatic analysis. *Endocr Connect* **2017**, *6*, 773-790.
47. Lakhter, A.J.; Pratt, R.E.; Moore, R.E.; Doucette, K.K.; Maier, B.F.; DiMeglio, L.A.; Sims, E.K. Beta cell extracellular vesicle miR-21-5p cargo is increased in response to inflammatory cytokines and serves as a biomarker of type 1 diabetes. *Diabetologia* **2018**, *61*, 1124-1134.
48. Grieco, G.E.; Cataldo, D.; Ceccarelli, E.; Nigi, L.; Catalano, G.; Brusco, N.; Mancarella, F.; Ventriglia, G.; Fondelli, C.; Guarino, E.; Crisci, I.; Sebastiani, G.; Dotta, F. Serum Levels of miR-148a and miR-21-5p Are Increased in Type 1 Diabetic Patients and Correlated with Markers of Bone Strength and Metabolism. *Noncoding RNA* **2018**, *4*, pii: E37.
49. Gholaminejad, A.; Abdul Tehrani, H.; Gholami Fesharaki, M. Identification of candidate microRNA biomarkers in diabetic nephropathy: a meta-analysis of profiling studies. *J Nephrol* **2018**, *31*, 813-831.
50. Long, B.; Gan, T.Y.; Zhang, R.C.; Zhang, Y.H. miR-23a Regulates Cardiomyocyte Apoptosis by Targeting Manganese Superoxide Dismutase. *Mol Cells* **2017**, *40*, 542-549.
51. Wang, S.; He, W.; Wang, C. MiR-23a Regulates the Vasculogenesis of Coronary Artery Disease by Targeting Epidermal Growth Factor Receptor. *Cardiovasc Ther* **2016**, *34*, 199-208.
52. Cong, X.; Li, Y.; Lu, N.; Dai, Y.; Zhang, H.; Zhao, X.; Liu, Y. Resveratrol attenuates the inflammatory reaction induced by ischemia/reperfusion in the rat heart. *Mol Med Rep* **2014**, *9*, 2528-2532.
53. Černá, V.; Ostašov, P.; Pitule, P.; Moláček, J.; Třeška, V.; Pešta, M. The Expression Profile of MicroRNAs in Small and Large Abdominal Aortic Aneurysms. *Cardiol Res Pract* **2019**, *2019*, 8645840.
54. Lozano-Bartolomé, J.; Llauradó, G.; Portero-Otin, M.; Altuna-Coy, A.; Rojo-Martínez, G.; Vendrell, J.; Jorba, R.; Rodríguez-Gallego, E.; Chacón, M.R. Altered Expression of miR-181a-5p and miR-23a-3p Is Associated With Obesity and TNFα-Induced Insulin Resistance. *J Clin Endocrinol Metab* **2018**, *103*, 1447-1458.
55. Dolz, S.; Górriz, D.; Tembl, J.I.; Sánchez, D.; Fortea, G.; Parkhutik, V.; Lago, A. Circulating MicroRNAs as Novel Biomarkers of Stenosis Progression in Asymptomatic Carotid Stenosis. *Stroke* **2017**, *48*, 10-16.
56. De Gonzalo-Calvo, D.; Cenarro, A.; Garlaschelli, K.; Pellegatta, F.; Vilades, D.; Nasarre, L.; Camino-Lopez, S.; Crespo, J.; Carreras, F.; Leta, R.; Catapano, A.L.; Norata, G.D.; Civeira, F.; Llorente-Cortes, V. Translating the microRNA signature of microvesicles derived from human coronary artery smooth muscle cells in patients with familial hypercholesterolemia and coronary artery disease. *J Mol Cell Cardiol* **2017**, *106*, 55–67.
57. Gecys, D.; Tatarunas, V.; Veikutiene, A.; Lesauskaite, V. New potential modulators of CYP4F2 enzyme activity in angina pectoris: hsa-miR-24-3p and hsa-miR-34a-5p. *Biomarkers* **2020**, *25*, 40-47.
58. Onrat, S.T.; Onrat, E.; Ercan Onay, E.; Yalım, Z.; Avşar, A. The Genetic Determination of the Differentiation between Ischemic Dilated Cardiomyopathy and Idiopathic Dilated Cardiomyopathy. *Genet Test Mol Biomarkers* **2018**, *22*, 644-651.
59. Tan, H.; Qi, J.; Fan, B.Y.; Zhang, J.; Su, F.F.; Wang, H.T. MicroRNA-24-3p Attenuates Myocardial Ischemia/Reperfusion Injury by Suppressing RIPK1 Expression in Mice. *Cell Physiol Biochem* **2018**, *51*, 46–62.
60. Xiao, X.; Lu, Z.; Lin, V.; May, A.; Shaw, D.H.; Wang, Z.; Che, B.; Tran, K.; Du, H.; Shaw, P.X. MicroRNA miR-24-3p Reduces Apoptosis and Regulates Keap1-Nrf2 Pathway in Mouse Cardiomyocytes Responding to Ischemia/Reperfusion Injury. *Oxid Med Cell Longev* **2018**, *2018*, 7042105.
61. Gao, J.; Liu, Q.G. The role of miR-26 in tumors and normal tissues. *Oncol Lett* **2011**, *2*, 1019-1023.
62. Zheng, L.; Lin, S.; Lv, C. MiR-26a-5p regulates cardiac fibroblasts collagen expression by targeting ULK1. *Sci Rep* **2018**, *8*, 2104.
63. Bye, A.; Røsjø, H.; Nauman, J.; Silva, G.J.; Follestad, T.; Omland, T.; Wisløff, U. Circulating microRNAs predict future fatal myocardial infarction in healthy individuals - The HUNT study. *J Mol Cell Cardiol* **2016**, *97*, 162-168.
64. Hsu, A.; Chen, S.J.; Chang, Y.S.; Chen, H.C.; Chu, P.H. Systemic approach to identify serum microRNAs as potential biomarkers for acute myocardial infarction. *Biomed Res Int* **2014**, *2014*, 418628.
65. Xing, X.; Guo, S.; Zhang, G.; Liu, Y.; Bi, S.; Wang, X.; Lu, Q. miR-26a-5p protects against myocardial ischemia/reperfusion injury by regulating the PTEN/PI3K/AKT signaling pathway. *Braz J Med Biol Res* **2020**, *53*, e9106.
66. Chouvarine, P.; Geldner, J.; Giagnorio, R.; Legchenko, E.; Bertram, H.; Hansmann, G. Trans-Right-Ventricle and Transpulmonary MicroRNA Gradients in Human Pulmonary Arterial Hypertension. *Pediatr Crit Care Med* **2019**, [Epub ahead of print].
67. Garavelli, S.; Bruzzaniti, S.; Tagliabue, E.; Prattichizzo, F.; Di Silvestre, D.; Perna, F.; La Sala, L.; Ceriello, A.; Mozzillo, E.; Fattorusso, V.; Mauri, P.; Puca, A.A.; Franzese, A.; Matarese, G.; Galgani, M.; de Candia, P. Blood Co-Circulating Extracellular microRNAs and Immune Cell Subsets Associate with Type 1 Diabetes Severity. *Int J Mol Sci* **2020**, *21*, pii: E477.
68. Ye, Y.; Hu, Z.; Lin, Y.; Zhang, C.; Perez-Polo, JR. Downregulation of microRNA-29 by antisense inhibitors and a PPAR-gamma agonist protects against myocardial ischaemia-reperfusion injury. *Cardiovasc Res* **2010**, *87*, 535-544.
69. Moraes, L.N.; Fernandez, G.J.; Vechetti-Júnior, I.J.; Freire, P.P.; Souza, R.W.A.; Villacis, R.A.R.; Rogatto, S.R.; Reis, P.P.; Dal-Pai-Silva, M.; Carvalho, R.F. Integration of miRNA and mRNA expression profiles reveals microRNA-regulated networks during muscle wasting in cardiac cachexia. *Sci Rep* **2017**, *7*, 6998.
70. Zhao, Y.; Yuan, Y.; Qiu, C. Underexpression of CACNA1C Caused by Overexpression of microRNA-29a Underlies the Pathogenesis of Atrial Fibrillation. *Med Sci Monit* **2016**, *22*, 2175-2181.
71. Zhang, L.; Zhang, Y.; Xue, S.; Ding, H.; Wang, Y.; Qi, H.; Wang, Y.; Zhu, W.; Li, P. Clinical significance of circulating microRNAs as diagnostic biomarkers for coronary artery disease. *J Cell Mol Med* **2020**, *24*, 1146-1150.
72. Wander, P.L.; Boyko, E.J.; Hevner, K.; Parikh, V.J.; Tadesse, M.G.; Sorensen, T.K.; Williams, M.A.; Enquobahrie, D.A. Circulating early- and mid-pregnancy microRNAs and risk of gestational diabetes. *Diabetes Res Clin Pract* **2017**, *132*, 1-9.
73. Kong, L.; Zhu, J.; Han, W.; Jiang, X.; Xu, M.; Zhao, Y.; Dong, Q.; Pang, Z.; Guan, Q.; Gao, L.; Zhao, J.; Zhao, L. Significance of serum microRNAs in pre-diabetes and newly diagnosed type 2 diabetes: a clinical study. *Acta Diabetol* **2011**, *48*, 61-69.
74. Widlansky, M.E.; Jensen, D.M.; Wang, J.; Liu, Y.; Geurts, A.M.; Kriegel, A.J.; Liu, P.; Ying, R.; Zhang, G.; Casati, M.; Chu, C.; Malik, M.; Branum, A.; Tanner, M.J.; Tyagi, S.; Usa, K.; Liang, M. miR-29 contributes to normal endothelial function and can restore it in cardiometabolic disorders. *EMBO Mol Med* **2018**, *10*, pii: e8046.
75. Bulent Vatan, M.; Kalaycı Yigin, A.; Akdemir, R.; Tarik Agac, M.; Akif Cakar, M.; Aksoy, M.; Tatli, E.; Kilic, H.; Gunduz, H.; Guzel, D.; Karacan, K. Altered Plasma MicroRNA Expression in Patients with Mitral Chordae Tendineae Rupture. *J Heart Valve Dis* **2016,** *25*, 580-588.
76. Gumus, G.; Giray, D.; Bobusoglu, O.; Tamer, L.; Karpuz, D.; Hallioglu, O. MicroRNA values in children with rheumatic carditis: a preliminary study. *Rheumatol Int* **2018**, *38*, 1199-1205.
77. Rogg, E.M.; Abplanalp, W.T.; Bischof, C.; John, D.; Schulz, M.H.; Krishnan, J.; Fischer, A.; Poluzzi, C.; Schaefer, L.; Bonauer, A.; Zeiher, A.M.; Dimmeler, S. Analysis of Cell Type-Specific Effects of MicroRNA-92a Provides Novel Insights Into Target Regulation and Mechanism of Action. *Circulation* **2018**, *138*, 2545-2558.
78. Marques, F.Z.; Vizi, D.; Khammy, O.; Mariani, J.A.; Kaye, D.M. The transcardiac gradient of cardio-microRNAs in the failing heart. *Eur J Heart Fail* **2016**, *18*, 1000-1008.
79. Liu, Y.; Li, Q.; Hosen, M.R.; Zietzer, A.; Flender, A.; Levermann, P.; Schmitz, T.; Frühwald, D.; Goody, P.; Nickenig, G.; Werner, N.; Jansen, F. Atherosclerotic Conditions Promote the Packaging of Functional MicroRNA-92a-3p Into Endothelial Microvesicles. *Circ Res* **2019**, *124*, 575-587.
80. Wiese, C.B.; Zhong, J.; Xu, Z.Q.; Zhang, Y.; Ramirez Solano, M.A.; Zhu, W.; Linton, M.F.; Sheng, Q.; Kon, V.; Vickers, K.C. Dual inhibition of endothelial miR-92a-3p and miR-489-3p reduces renal injury-associated atherosclerosis. *Atherosclerosis* **2019**, *282*, 121-131.
81. Moncini, S.; Salvi, A.; Zuccotti, P.; Viero, G.; Quattrone, A.; Barlati, S.; De Petro, G.; Venturin, M.; Riva, P. The role of miR-103 and miR-107 in regulation of CDK5R1 expression and in cellular migration. *PLoS One* **2011**, *6*, e20038.
82. Huang, L.; Li, L.; Chen, X.; Zhang, H.; Shi, Z. MiR-103a targeting Piezo1 is involved in acute myocardial infarction through regulating endothelium function. *Cardiol J* **2016**, *23*, 556-562.
83. Deng, B.; Du, J.; Hu, R.; Wang, A.P.; Wu, W.H.; Hu, C.P.; Li, Y.J.; Li, X.H. MicroRNA-103/107 is involved in hypoxia-induced proliferation of pulmonary arterial smooth muscle cells by targeting HIF-1β. *Life Sci* **2016**, *147*, 117-124.
84. Trajkovski, M.; Hausser, J.; Soutschek, J.; Bhat, B.; Akin, A.; Zavolan, M.; Heim, M.H.; Stoffel, M. MicroRNAs 103 and 107 regulate insulin sensitivity. *Nature* **2011**, *474*, 649-653.
85. Assmann, T.S.; Recamonde-Mendoza, M.; Puñales, M.; Tschiedel, B.; Canani, L.H.; Crispim, D. MicroRNA expression profile in plasma from type 1 diabetic patients: Case-control study and bioinformatic analysis. *Diabetes Res Clin Pract* **2018**, *141*, 35-46.
86. Shaham, L.; Binder, V.; Gefen, N.; Borkhardt, A.; Izraeli, S. MiR-125 in normal and malignant hematopoiesis. *Leukemia* **2012**, *26*, 2011-2018.
87. Tiedt, S.; Prestel, M.; Malik, R.; Schieferdecker, N.; Duering, M.; Kautzky, V.; Stoycheva, I.; Böck, J.; Northoff, B.H.; Klein, M.; Dorn, F.; Krohn, K.; Teupser, D.; Liesz, A.; Plesnila, N.; Holdt, L.M.; Dichgans, M. RNA-Seq Identifies Circulating miR-125a-5p, miR-125b-5p, and miR-143-3p as Potential Biomarkers for Acute Ischemic Stroke. *Circ Res* **2017**, *121*, 970-980.
88. Jia, K.; Shi, P.; Han, X.; Chen, T.; Tang, H.; Wang, J. Diagnostic value of miR-30d-5p and miR-125b-5p in acute myocardial infarction. *Mol Med Rep* **2016**, *14*, 184-194.
89. Bayoumi, A.S.; Park, K.M.; Wang, Y.; Teoh, J.P.; Aonuma, T.; Tang, Y.; Su, H. ; Weintraub, N.L.; Kim, I.M. A carvedilol-responsive microRNA, miR-125b-5p protects the heart from acute myocardial infarction by repressing pro-apoptotic bak1 and klf13 in cardiomyocytes. *J Mol Cell Cardiol* **2018**, *114*, 72-82.
90. Lamadrid-Romero, M.; Solís, K.H.; Cruz-Reséndiz, M.S.; Pérez, J.E.; Díaz, N.F.; Flores-Herrera, H.; García-López, G.; Perichart, O.; Reyes-Muñoz, E.; Arenas-Huertero, F.; Eguía-Aguilar, P.; Molina-Hernández, A. Central nervous system development-related microRNAs levels increase in the serum of gestational diabetic women during the first trimester of pregnancy. *Neurosci Res* **2018**, *130*, 8-22.
91. Satake, E.; Pezzolesi, M.G.; Md Dom, Z.I.; Smiles, A.M.; Niewczas, M.A.; Krolewski, A.S. Circulating miRNA Profiles Associated With Hyperglycemia in Patients With Type 1 Diabetes. *Diabetes* **2018**, *67*, 1013-1023.
92. Samandari, N.; Mirza, A.H.; Kaur, S.; Hougaard, P.; Nielsen, L.B.; Fredheim, S.; Mortensen, H.B.; Pociot, F. Influence of Disease Duration on Circulating Levels of miRNAs in Children and Adolescents with New Onset Type 1 Diabetes. *Noncoding RNA* **2018**, *4*, pii: E35.
93. Yu, C.Y.; Yang, C.Y.; Rui, Z.L. MicroRNA-125b-5p improves pancreatic β-cell function through inhibiting JNK signaling pathway by targeting DACT1 in mice with type 2 diabetes mellitus. *Life Sci* **2019**, *224*, 67-75.
94. Wu, X.J.; Zhao, Z.F.; Kang, X.J.; Wang, H.J.; Zhao, J.; Pu, X.M. MicroRNA-126-3p suppresses cell proliferation by targeting PIK3R2 in Kaposi's sarcoma cells. *Oncotarget* **2016**, *7*, 36614-36621.
95. Matsha, T.E.; Kengne, A.P.; Hector, S.; Mbu, D.L.; Yako, Y.Y.; Erasmus, R.T. MicroRNA profiling and their pathways in South African individuals with prediabetes and newly diagnosed type 2 diabetes mellitus. *Oncotarget* **2018**, *9*, 30485-30498.
96. Tryggestad, J.B.; Vishwanath, A.; Jiang, S.; Mallappa, A.; Teague, A.M.; Takahashi, Y.; Thompson, D.M.; Chernausek, S.D. Influence of gestational diabetes mellitus on human umbilical vein endothelial cell miRNA. *Clin Sci (Lond)* **2016**, *130*, 1955-1967.
97. Lan, X.; Wu, L.; Wu, N.; Chen, Q.; Li, Y.; Du, X.; Wei, C.; Feng, L.; Li, Y.; Osoro, E.K.; Sun, M.; Ning, Q.; Yan, X.; Yang, X.; Li, D.; Lu, S. Long Noncoding RNA lnc-HC Regulates PPARγ-Mediated Hepatic Lipid Metabolism through miR-130b-3p. *Mol Ther Nucleic Acids* **2019**, *18*, 954-965.
98. Zhang, J.; Jazii, F.R.; Haghighi, M.M.; Alvares, D.; Liu, L.; Khosraviani, N.; Adeli, K. miR-130b is a potent stimulator of hepatic very-low-density lipoprotein assembly and secretion via marked induction of microsomal triglyceride transfer protein. *Am J Physiol Endocrinol Metab* **2020**, *318*, E262-E275.
99. Li, P.; Zhang, Q.; Wu, X.; Yang, X.; Zhang, Y.; Li, Y.; Jiang, F. Circulating microRNAs serve as novel biological markers for intracranial aneurysms. *J Am Heart Assoc* **2014**, *3*, e000972.
100. Tian, C.; Li, Z.; Yang, Z.; Huang, Q.; Liu, J.; Hong, B. Plasma MicroRNA-16 Is a Biomarker for Diagnosis, Stratification, and Prognosis of Hyperacute Cerebral Infarction. *PLoS One* **2016**, *11*, e0166688.
101. Prabu, P.; Rome, S.; Sathishkumar, C.; Aravind, S.; Mahalingam, B.; Shanthirani, C.S.; Gastebois, C.; Villard, A.; Mohan, V.; Balasubramanyam, M. Circulating MiRNAs of 'Asian Indian Phenotype' Identified in Subjects with Impaired Glucose Tolerance and Patients with Type 2 Diabetes. *PLoS One* **2015**, *10*, e0128372.
102. Feng, T.; Li, K.; Zheng, P.; Wang, Y.; Lv, Y.; Shen, L.; Chen, Y.; Xue, Z.; Li, B.; Jin, L.; Yao, Y. Weighted Gene Coexpression Network Analysis Identified MicroRNA Coexpression Modules and Related Pathways in Type 2 Diabetes Mellitus. *Oxid Med Cell Longev* **2019**, *2019*, 9567641.
103. Liang, H.W.; Yang, X.; Wen, D.Y.; Gao, L.; Zhang, X.Y.; Ye, Z.H.; Luo, J.; Li, Z.Y.; He, Y.; Pang, Y.Y.; Chen, G. Utility of miR 133a 3p as a diagnostic indicator for hepatocellular carcinoma: An investigation combined with GEO, TCGA, meta analysis and bioinformatics. *Mol Med Rep* **2018**, *17*, 1469-1484.
104. van Rooij, E.; Olson, E.N. MicroRNAs: powerful new regulators of heart disease and provocative therapeutic targets. *J Clin Invest* **2007**, *117*, 2369-2376.
105. Wang, J.; Xu, R.; Lin, F.; Zhang, S.; Zhang, G.; Hu, S.; Zheng, Z. MicroRNA: novel regulators involved in the remodeling and reverse remodeling of the heart. *Cardiology* **2009**, *113*, 81-88.
106. Kukreja, R.C.; Yin, C.; Salloum, F.N. MicroRNAs: new players in cardiac injury and protection. *Mol Pharmacol* **2011**, *80*, 558-564.
107. Duisters, R.F.; Tijsen, A.J.; Schroen, B.; Leenders, J.J.; Lentink, V.; van der Made, I.; Herias, V.; van Leeuwen, R.E.; Schellings, M.W.; Barenbrug, P.; Maessen, J.G.; Heymans, S.; Pinto, Y.M.; Creemers, E.E. miR-133 and miR-30 regulate connective tissue growth factor: implications for a role of microRNAs in myocardial matrix remodeling. *Circ Res* **2009**, *104*, 170-178.
108. Liu, W.; Ling, S.; Sun, W.; Liu, T.; Li, Y.; Zhong, G.; Zhao, D.; Zhang, P.; Song, J.; Jin, X.; Xu, Z.; Song, H.; Li, Q.; Liu, S.; Chai, M.; Dai, Q.; He, Y.; Fan, Z.; Zhou, Y.J.; Li, Y. Circulating microRNAs correlated with the level of coronary artery calcification in symptomatic patients. *Sci Rep* **2015**, *5*, 16099.
109. Jiang, Y.; Zhang, M.; He, H.; Chen, J.; Zeng, H.; Li, J.; Duan, R. MicroRNA/mRNA profiling and regulatory network of intracranial aneurysm. *BMC Med Genomics* **2013**, *6*, 36.
110. Liu, H.; Xiong, W.; Liu, F.; Lin, F.; He, J.; Liu, C.; Lin, Y.; Dong, S. Significant role and mechanism of microRNA-143-3p/KLLN axis in the development of coronary heart disease. *Am J Transl Res* **2019**, *11*, 3610-3619.
111. Li, C.; Li, J.; Xue, K.; Zhang, J.; Wang, C.; Zhang, Q.; Chen, X.; Gao, C.; Yu, X.; Sun, L. MicroRNA-143-3p promotes human cardiac fibrosis via targeting sprouty3 after myocardial infarction. *J Mol Cell Cardiol* **2019**, *129*, 281-292.
112. Yu, B.; Zhao, Y.; Zhang, H.; Xie, D.; Nie, W.; Shi, K. Inhibition of microRNA-143-3p attenuates myocardial hypertrophy by inhibiting inflammatory response. *Cell Biol Int* **2018**, *42*, 1584-1593.
113. Jiao, M.; You, H.Z.; Yang, X.Y.; Yuan, H.; Li, Y.L.; Liu, W.X.; Jin, M.; Du, J. Circulating microRNA signature for the diagnosis of childhood dilated cardiomyopathy. *Sci Rep* **2018**, *8*, 724.
114. Deng, L.; Blanco, F.J.; Stevens, H.; Lu, R.; Caudrillier, A.; McBride, M.; McClure, J.D.; Grant, J.; Thomas, M.; Frid, M.; Stenmark, K.; White, K.; Seto, A.G.; Morrell, N.W.; Bradshaw, A.C.; MacLean, M.R.; Baker, A.H. MicroRNA-143 Activation Regulates Smooth Muscle and Endothelial Cell Crosstalk in Pulmonary Arterial Hypertension. *Circ Res* **2015**, *117*, 870-883.
115. Shi, L.; Tian, C.; Sun, L.; Cao, F.; Meng, Z. The lncRNA TUG1/miR-145-5p/FGF10 regulates proliferation and migration in VSMCs of hypertension. *Biochem Biophys Res Commun* **2018**, *501*, 688-695.
116. Yang, X.; Niu, X.; Xiao, Y.; Lin, K.; Chen, X. MiRNA expression profiles in healthy OSAHS and OSAHS with arterial hypertension: potential diagnostic and early warning markers. *Respir Res* **2018**, *19*, 194.
117. Toro, R.; Blasco-Turrión, S.; Morales-Ponce, F.J.; Gonzalez, P.; Martínez-Camblor, P.; López-Granados, A.; Brugada, R.; Campuzano, O.; Pérez-Serra, A.; Rosa Longobardo, F.; Mangas, A.; Llorente-Cortes, V.; de Gonzalo-Calvo, D. Plasma microRNAs as biomarkers for Lamin A/C-related dilated cardiomyopathy. *J Mol Med (Berl)* **2018**, *96*, 845-856.
118. Yuan, M;, Zhang, L;, You, F.; Zhou, J.; Ma, Y.; Yang, F.; Tao, L. MiR-145-5p regulates hypoxia-induced inflammatory response and apoptosis in cardiomyocytes by targeting CD40. *Mol Cell Biochem* **2017**, *431*, 123-131.
119. Wu, G.; Tan, J.; Li, J.; Sun, X.; Du, L.; Tao, S. miRNA-145-5p induces apoptosis after ischemia-reperfusion by targeting dual specificity phosphatase 6. *J Cell Physiol* **2019**, *234*, 16281–16289.
120. Xie, X.; Peng, L.; Zhu, J.; Zhou, Y.; Li, L.; Chen, Y.; Yu, S.; Zhao, Y. miR-145-5p/Nurr1/TNF-α Signaling-Induced Microglia Activation Regulates Neuron Injury of Acute Cerebral Ischemic/Reperfusion in Rats. *Front Mol Neurosci* **2017**, *10*, 383.
121. Nunez Lopez, Y.O.; Retnakaran, R.; Zinman, B.; Pratley, R.E.; Seyhan, A.A. Predicting and understanding the response to short-term intensive insulin therapy in people with early type 2 diabetes. *Mol Metab* **2019**, *20*, 63-78.
122. Zhang, J.; Cui, C.; Xu, H. Downregulation of miR-145-5p elevates retinal ganglion cell survival to delay diabetic retinopathy progress by targeting FGF5. *Biosci Biotechnol Biochem* **2019**, *83*, 1655-1662.
123. Zamanian Azodi, M.; Rezaei-Tavirani, M.; Rezaei-Tavirani, M.; Robati, R.M. Gestational Diabetes Mellitus Regulatory Network Identifies hsa-miR-145-5p and hsa-miR-875-5p as Potential Biomarkers. *Int J Endocrinol Metab* **2019**, *17*, e86640.
124. Taganov, K.D.; Boldin, M.P.; Chang, K.J.; Baltimore, D. NF-kappaB-dependent induction of microRNA miR-146, an inhibitor targeted to signaling proteins of innate immune responses. *Proc Natl Acad Sci U S A* **2006**, *103*, 12481-12486.
125. Paterson, M.R.; Kriegel, A.J. MiR-146a/b: a family with shared seeds and different roots. *Physiol Genomics* **2017**, *49*, 243-252.
126. Zhang, X.; Ye, Z.H.; Liang, H.W.; Ren, F.H.; Li, P.; Dang, Y.W.; Chen, G. Down-regulation of miR-146a-5p and its potential targets in hepatocellular carcinoma validated by a TCGA- and GEO-based study. *FEBS Open Bio* **2017**, *7*, 504-521.
127. Wang, X.; Ha, T.; Liu, L.; Zou, J.; Zhang, X.; Kalbfleisch, J.; Gao, X.; Williams, D.; Li, C. Increased expression of microRNA-146a decreases myocardial ischaemia/reperfusion injury. *Cardiovasc Res* **2013**, *97*, 432-442.
128. Quan, X.; Ji, Y.; Zhang, C.; Guo, X.; Zhang, Y.; Jia, S.; Ma, W.; Fan, Y.; Wang, C. Circulating MiR-146a May be a Potential Biomarker of Coronary Heart Disease in Patients with Subclinical Hypothyroidism. *Cell Physiol Biochem* **2018**, *45*, 226-236.
129. Li, S.H. ; Chen, L. ; Pang, X.M.; Su, S.Y.; Zhou, X.; Chen, C.Y.; Huang, L.G.; Li, J.P.; Liu, J.L. Decreased miR-146a expression in acute ischemic stroke directly targets the Fbxl10 mRNA and is involved in modulating apoptosis. *Neurochem Int* **2017**, *107*, 156-67.
130. Barberio, M.D.; Kasselman, L.J.; Playford, M.P.; Epstein, S.B.; Renna, H.A.; Goldberg, M.; DeLeon, J.; Voloshyna, I.; Barlev, A.; Salama, M.; Ferrante, S.C.; Nadler, E.P.; Mehta, N.; Reiss, A.B.; Freishtat, R.J. Cholesterol efflux alterations in adolescent obesity: role of adipose-derived extracellular vesical microRNAs. *J Transl Med* **2019**, *17*, 232.
131. Gaudet, A.D.; Fonken, L.K.; Gushchina, L.V.; Aubrecht, T.G.; Maurya, S.K.; Periasamy, M.; Nelson, R.J.; Popovich, P.G. miR-155 Deletion in Female Mice Prevents Diet-Induced Obesity. *Sci Rep* **2016**, *6*, 22862.
132. Chen, L.; Zheng, S.Y.; Yang, C.Q.; Ma, B.M.; Jiang, D. MiR-155-5p inhibits the proliferation and migration of VSMCs and HUVECs in atherosclerosis by targeting AKT1. *Eur Rev Med Pharmacol Sci* **2019**, *23*, 2223-2233.
133. Zhu, M.; Wei, Y.; Geißler, C.; Abschlag, K.; Corbalán Campos, J.; Hristov, M.; Möllmann, J.; Lehrke, M.; Karshovska, E.; Schober, A. Hyperlipidemia-Induced MicroRNA-155-5p Improves β-Cell Function by Targeting Mafb. *Diabetes* **2017**, *66*, 3072-3084.
134. Li, S.; Lee, C.; Song, J.; Lu, C.; Liu, J.; Cui, Y.; Liang, H.; Cao, C.; Zhang, F.; Chen, H. Circulating microRNAs as potential biomarkers for coronary plaque rupture. *Oncotarget* **2017**, *8*, 48145-48156.
135. Mukai, N.; Nakayama, Y.; Murakami, S.; Tanahashi, T.; Sessler, D.I.; Ishii, S.; Ogawa, S.; Tokuhira, N.; Mizobe, T.; Sawa, T.; Nakajima, Y. Potential contribution of erythrocyte microRNA to secondary erythrocytosis and thrombocytopenia in congenital heart disease. *Pediatr Res* **2018**, *83*, 866-873.
136. Klimczak, D.; Kuch, M.; Pilecki, T.; Żochowska, D.; Wirkowska, A.; Pączek, L. Plasma microRNA-155-5p is increased among patients with chronic kidney disease and nocturnal hypertension. *J Am Soc Hypertens* **2017**, *11*, 831-841.e4.
137. Wang, M.; Sun, L.; Ding, W.; Cai, S.; Zhao, Q. Ablation alleviates atrial fibrillation by regulating the signaling pathways of endothelial nitric oxide synthase/nitric oxide via miR-155-5p and miR-24-3p. *J Cell Biochem* **2019**, *120*, 4451-4462.
138. Sun, X.; Sit, A.; Feinberg, M.W. Role of miR-181 family in regulating vascular inflammation and immunity. *Trends Cardiovasc Med* **2014**, *24*, 105-112.
139. Hulsmans, M.; Sinnaeve, P.; Van der Schueren, B.; Mathieu, C.; Janssens, S.; Holvoet, P. Decreased miR-181a expression in monocytes of obese patients is associated with the occurrence of metabolic syndrome and coronary artery disease. *J Clin Endocrinol Metab* **2012**, *97*, E1213-8.
140. Du, X.; Yang, Y.; Xu, C.; Peng, Z.; Zhang, M.; Lei, L.; Gao, W.; Dong, Y.; Shi, Z.; Sun, X.; Wang, Z.; Li, X.; Li, X.; Liu, G. Upregulation of miR-181a impairs hepatic glucose and lipid homeostasis. *Oncotarget* **2017**, *8*, 91362-91378.
141. Wu, J.; Fan, C.L.; Ma, L.J.; Liu, T.; Wang, C.; Song, J.X.; Lv, Q.S.; Pan, H.; Zhang, C.N.; Wang, J.J. Distinctive expression signatures of serum microRNAs in ischaemic stroke and transient ischaemic attack patients. *Thromb Haemost* **2017**, *117*, 992-1001.
142. Zhu, J.; Yao, K.; Wang, Q.; Guo, J.; Shi, H.; Ma, L.; Liu, H.; Gao, W.; Zou, Y.; Ge, J. Circulating miR-181a as a Potential Novel Biomarker for Diagnosis of Acute Myocardial Infarction. *Cell Physiol Biochem* **2016**, *40*, 1591-1602.
143. Nabih, E.S.; Andrawes, N.G. The Association Between Circulating Levels of miRNA-181a and Pancreatic Beta Cells Dysfunction via SMAD7 in Type 1 Diabetic Children and Adolescents. *J Clin Lab Anal* **2016**, *30*, 727-731.
144. He, J.F.; Luo, Y.M.; Wan, X.H.; Jiang, D. Biogenesis of MiRNA-195 and its role in biogenesis, the cell cycle, and apoptosis*. J Biochem Mol Toxicol* **2011**, *25*, 404-408.
145. van Rooij, E.; Sutherland, L.B.; Liu, N.; Williams, A.H.; McAnally, J.; Gerard, R.D.; Richardson, J.A.; Olson, E.N. A signature pattern of stress-responsive microRNAs that can evoke cardiac hypertrophy and heart failure. *Proc Natl Acad Sci U S A* **2006**, *103*, 18255-18260.
146. You, X.Y.; Huang, J.H.; Liu, B.; Liu, S.J.; Zhong, Y.; Liu, S.M. HMGA1 is a new target of miR-195 involving isoprenaline-induced cardiomyocyte hypertrophy. *Biochemistry (Mosc)* **2014**, *79*, 538-544.
147. Zampetaki, A.; Attia, R.; Mayr, U.; Gomes, R.S.; Phinikaridou, A.; Yin, X.; Langley, S.R.; Willeit, P.; Lu, R.; Fanshawe, B.; Fava, M.; Barallobre-Barreiro, J.; Molenaar, C.; So, P.W.; Abbas, A.; Jahangiri, M.; Waltham, M.; Botnar, R.; Smith, A.; Mayr, M. Role of miR-195 in aortic aneurysmal disease. *Circ Res* **2014**, *115*, 857-866.
148. Du, J.; Zheng, R.; Xiao, F.; Zhang, S.; He, K.; Zhang, J.; Shao, Y. Downregulated MicroRNA-195 in the Bicuspid Aortic Valve Promotes Calcification of Valve Interstitial Cells via Targeting SMAD7. *Cell Physiol Biochem* **2017**, *44*, 884-896.
149. Tagoma, A.; Alnek, K.; Kirss, A.; Uibo, R.; Haller-Kikkatalo, K. MicroRNA profiling of second trimester maternal plasma shows upregulation of miR-195-5p in patients with gestational diabetes. *Gene* **2018**, *672*, 137-142.
150. Collares, C.V.; Evangelista, A.F.; Xavier, D.J.; Rassi, D.M.; Arns, T.; Foss-Freitas, M.C.; Foss, M.C.; Puthier, D.; Sakamoto-Hojo, E.T.; Passos, G.A.; Donadi, E.A. Identifying common and specific microRNAs expressed in peripheral blood mononuclear cell of type 1, type 2, and gestational diabetes mellitus patients. *BMC Res Notes* **2013**, *6*, 491.
151. Massaro, J.D.; Polli, C.D.; Costa E Silva, M.; Alves, C.C.; Passos, G.A.; Sakamoto-Hojo, E.T.; Rodrigues de Holanda Miranda, W.; Bispo Cezar, N.J.; Rassi, D.M.; Crispim, F.; Dib, S.A.; Foss-Freitas, M.C.; Pinheiro, D.G.; Donadi, E.A. Post-transcriptional markers associated with clinical complications in Type 1 and Type 2 diabetes mellitus. *Mol Cell Endocrinol* **2019**, *490*, 1-14.
152. Li, M.; Luan, L.; Liu, Q.; Liu, Y.; Lan, X.; Li, Z.; Liu, W. MiRNA-199a-5p Protects Against Cerebral Ischemic Injury by Down-Regulating DDR1 in Rats. *World Neurosurg* **2019**, *131*, e486-e494.
153. Yan, M.; Yang, S.; Meng, F.; Zhao, Z.; Tian, Z.; Yang, P. MicroRNA 199a-5p induces apoptosis by targeting JunB. *Sci Rep* **2018**, *8*, 6699.
154. Lynch, S.M.; Ward, M.; McNulty, H.; Angel, C.Z.; Horigan, G.; Strain, J.J.; Purvis, J.; Tackett, M.; McKenna, D.J. Serum levels of miR-199a-5p correlates with blood pressure in premature cardiovascular disease patients homozygous for the MTHFR 677C > T polymorphism. *Genomics* **2020**, *112*, 669-676.
155. Tian, X.; Yu, C.; Shi, L.; Li, D.; Chen, X.; Xia, D.; Zhou, J.; Xu, W.; Ma, C.; Gu, L.; An, Y. MicroRNA-199a-5p aggravates primary hypertension by damaging vascular endothelial cells through inhibition of autophagy and promotion of apoptosis. *Exp Ther Med* **2018**, *16*, 595-602.
156. Zhou, Y.; Pang, B.; Xiao, Y.; Zhou, S.; He, B.; Zhang, F.; Liu, W.; Peng, H.; Li, P. The protective microRNA-199a-5p-mediated unfolded protein response in hypoxic cardiomyocytes is regulated by STAT3 pathway. *J Physiol Biochem* **2019**, *75*, 73-81.
157. Liu, Y.; Liu, G.; Zhang, H.; Wang, J. MiRNA-199a-5p influences pulmonary artery hypertension via downregulating Smad3. *Biochem Biophys Res Commun* **2016**, *473*, 859-866.
158. Wang, J.; Yu, G. A Systems Biology Approach to Characterize Biomarkers for Blood Stasis Syndrome of Unstable Angina Patients by Integrating MicroRNA and Messenger RNA Expression Profiling. *Evid Based Complement Alternat Med* **2013**, *2013*, 510208.
159. Yu, L.; Gu, T.; Shi, E.; Wang, Y.; Fang, Q.; Wang, C. Dysregulation of renal microRNA expression after deep hypothermic circulatory arrest in rats. *Eur J Cardiothorac Surg* **2016**, *49*, 1725-1731.
160. Hirt, M.N.; Werner, T.; Indenbirken, D.; Alawi, M.; Demin, P.; Kunze, A.C.; Stenzig, J.; Starbatty, J.; Hansen, A.; Fiedler, J.; Thum, T.; Eschenhagen, T. Deciphering the microRNA signature of pathological cardiac hypertrophy by engineered heart tissue- and sequencing-technology. *J Mol Cell Cardiol* **2015**, *81*, 1-9.
161. Aguado-Fraile, E.; Ramos, E.; Conde, E.; Rodríguez, M.; Martín-Gómez, L.; Lietor, A.; Candela, Á.; Ponte, B.; Liaño, F.; García-Bermejo, M.L. A Pilot Study Identifying a Set of microRNAs As Precise Diagnostic Biomarkers of Acute Kidney Injury. *PLoS One* **2015**, *10*, e0127175.
162. Ma, H.; Chen, P.; Sang, C.; Huang, D.; Geng, Q.; Wang, L. Modulation of apoptosis-related microRNAs following myocardial infarction in fat-1 transgenic mice vs wild-type mice. *J Cell Mol Med* **2018**, *22*, 5698-5707.
163. Qiao, X.R.; Wang, L.; Liu, M.; Tian, Y.; Chen, T. MiR-210-3p attenuates lipid accumulation and inflammation in atherosclerosis by repressing IGF2. *Biosci Biotechnol Biochem* **2020**, *84*, 321-329.
164. Derda, A.A.; Pfanne, A.; Bwangär, C.; Schimmel, K.; Kennel, P.J.; Xiao, K.; Schulze, P.C.; Bauersachs, J.; Thum, T. Blood-based microRNA profiling in patients with cardiac amyloidosis. *PLoS One* **2018**, *13*, e0204235.
165. Verjans, R.; Peters, T.; Beaumont, F.J.; van Leeuwen, R.; van Herwaarden, T.; Verhesen, W.; Munts, C.; Bijnen, M.; Henkens, M.; Diez, J.; de Windt, L.J.; van Nieuwenhoven, F.A.; van Bilsen, M.; Goumans, M.J.; Heymans, S.; González, A.; Schroen, B. MicroRNA-221/222 Family Counteracts Myocardial Fibrosis in Pressure Overload-Induced Heart Failure. *Hypertension* **2018**, *71*, 280-288.
166. Zhuang, X.; Li, R.; Maimaitijiang, A.; Liu, R.; Yan, F.; Hu, H.; Gao, X.; Shi, H. miR-221-3p inhibits oxidized low-density lipoprotein induced oxidative stress and apoptosis via targeting a disintegrin and metalloprotease-22. *J Cell Biochem* **2019**, *120*, 6304-6314.
167. Pereira-da-Silva, T.; Coutinho Cruz, M.; Carrusca, C.; Cruz Ferreira, R.; Napoleão, P.; Mota Carmo, M. Circulating microRNA profiles in different arterial territories of stable atherosclerotic disease: a systematic review. *Am J Cardiovasc Dis* **2018**, *8*, 1-13.
168. Coffey, S.; Williams, M.J.; Phillips, L.V.; Galvin, I.F.; Bunton, R.W.; Jones, G.T. Integrated microRNA and messenger RNA analysis in aortic stenosis. *Sci Rep* **2016**, *6*, 36904.
169. Coskunpinar, E.; Cakmak, H.A.; Kalkan, A.K.; Tiryakioglu, N.O.; Erturk, M.; Ongen, Z. Circulating miR-221-3p as a novel marker for early prediction of acute myocardial infarction. *Gene* **2016**, *591*, 90-96.
170. Sørensen, S.S.; Nygaard, A.B.; Nielsen, M.Y.; Jensen, K.; Christensen, T. miRNA expression profiles in cerebrospinal fluid and blood of patients with acute ischemic stroke. *Transl Stroke Res* **2014**, *5*, 711-718.
171. Gusar, V.A.; Timofeeva, A.V.; Zhanin, I.S.; Shram, S.I.; Pinelis, V.G. Estimation of Time-Dependent microRNA Expression Patterns in Brain Tissue, Leukocytes, and Blood Plasma of Rats under Photochemically Induced Focal Cerebral Ischemia. *Mol Biol (Mosk)* **2017**, *51*, 683-695.
172. Nie, X.; Chen, Y.; Tan, J.; Dai, Y.; Mao, W.; Qin, G.; Ye, S.; Sun, J.; Yang, Z.; Chen, J. MicroRNA-221-3p promotes pulmonary artery smooth muscle cells proliferation by targeting AXIN2 during pulmonary arterial hypertension. *Vascul Pharmacol* **2019**, *116*, 24-35.
173. Villard, A.; Marchand, L.; Thivolet, C.; Rome, S. Diagnostic Value of Cell-free Circulating MicroRNAs for Obesity and Type 2 Diabetes: A Meta-analysis. *J Mol Biomark Diagn* **2015**, *6*, pii: 251.
174. Wang, L.; Xu, L.; Xu, M.; Liu, G.; Xing, J.; Sun, C.; Ding, H. Obesity-Associated MiR-342-3p Promotes Adipogenesis of Mesenchymal Stem Cells by Suppressing CtBP2 and Releasing C/EBPα from CtBP2 Binding. *Cell Physiol Biochem* **2015**, *35*, 2285-2298.
175. Hezova, R.; Slaby, O.; Faltejskova, P.; Mikulkova, Z.; Buresova, I.; Raja, K.R.; Hodek, J.; Ovesna, J.; Michalek, J. microRNA-342, microRNA-191 and microRNA-510 are differentially expressed in T regulatory cells of type 1 diabetic patients. *Cell Immunol* **2010***, 260*, 70-74.
176. Eissa, S.; Matboli, M.; Bekhet, M.M. Clinical verification of a novel urinary microRNA panal: 133b, -342 and -30 as biomarkers for diabetic nephropathy identified by bioinformatics analysis. *Biomed Pharmacother* **2016**, *83*, 92-99.
177. Cheng, S.; Cui, Y.; Fan, L.; Mu, X.; Hua, Y. T2DM inhibition of endothelial miR-342-3p facilitates angiogenic dysfunction via repression of FGF11 signaling. *Biochem Biophys Res Commun* **2018**, *503*, 71-78.
178. Khalyfa, A.; Kheirandish-Gozal, L.; Bhattacharjee, R.; Khalyfa, A.A.; Gozal, D. Circulating microRNAs as Potential Biomarkers of Endothelial Dysfunction in Obese Children. *Chest* **2016**, *149*, 786-800.
179. Hoekstra, M. MicroRNA-499-5p: a therapeutic target in the context of cardiovascular disease. *Ann Transl Med* **2016**, *4*, 539.
180. Zhao, L.; Wang, B.; Zhang, W.; Sun, L. Effect of miR-499a-5p on damage of cardiomyocyte induced by hypoxia-reoxygenation via downregulating CD38 protein. *J Cell Biochem* **2020**, *121*, 996-1004.
181. Neshati, V.; Mollazadeh, S.; Fazly Bazzaz, B.S.; de Vries, A.A.F.; Mojarrad, M.; Naderi-Meshkin, H.; Neshati, Z.; Mirahmadi, M.; Kerachian, M.A. MicroRNA-499a-5p Promotes Differentiation of Human Bone Marrow-Derived Mesenchymal Stem Cells to Cardiomyocytes. *Appl Biochem Biotechnol* **2018**, *186*, 245-255.
182. Boštjančič, E.; Zidar, N.; Glavač, D. MicroRNAs and cardiac sarcoplasmic reticulum calcium ATPase-2 in human myocardial infarction: expression and bioinformatic analysis. *BMC Genomics* **2012**, *13*, 552.
183. Salinas, J.; Lin, H.; Aparico, H.J.; Huan, T.; Liu, C.; Rong, J.; Beiser, A.; Himali, J.J.; Freedman, J.E.; Larson, M.G.; Rosand, J.; Soreq, H.; Levy, D.; Seshadri, S. Whole blood microRNA expression associated with stroke: Results from the Framingham Heart Study. *PLoS One* **2019**, *14*, e0219261.
184. Baldeón Rojas, L.; Weigelt, K.; de Wit, H.; Ozcan, B.; van Oudenaren, A.; Sempértegui, F.; Sijbrands, E.; Grosse, L.; van Zonneveld, A.J.; Drexhage, H.A.; Leenen, P.J. Study on inflammation-related genes and microRNAs, with special emphasis on the vascular repair factor HGF and miR-574-3p, in monocytes and serum of patients with T2D. *Diabetol Metab Syndr* **2016**, *8*, 6.
