# Supplementary material for: Association Analysis in Young and Middle-Aged Mothers—Relation between Expression of Cardiovascular Disease Associated MicroRNAs and Abnormal Clinical Findings
Source: J Pers Med. 2021 Jan 11;11(1):39. doi: 10.3390/jpm11010039 (PMC7826744; doi:10.3390/jpm11010039)
Supplement: Supplementary file 1 [file jpm-11-00039-s001.zip › Supplementary Material/Supplementary Table 2.docx]

**Supplementary Table S2.**

**Table S2. The association analysis between occasional physical activity and microRNA gene expression**

| MicroRNA | Median  Women without sport activity  (n=295) | Median  Women with any sport activity  (n=183) | Mann-Whitney test  P value |
| --- | --- | --- | --- |
| miR-1-3p | 0.116 | 0.084 | 0.322 |
| miR-16-5p | 1.093 | 1.053 | 0.254 |
| miR-17-5p | 1.316 | 1.229 | 0.325 |
| miR-20a-5p | 1.305 | 1.164 | 0.391 |
| miR-20b-5p | 1.332 | 1.236 | 0.447 |
| miR-21-5p | 0.268 | 0.257 | 0.439 |
| miR-23a-3p | 0.175 | 0.138 | 0.220 |
| miR-24-3p | 0.244 | 0.214 | 0.584 |
| miR-26a-5p | 0.500 | 0.440 | 0.213 |
| miR-29a-3p | 0.283 | 0.264 | 0.383 |
| miR-92a-3p | 1.809 | 1.824 | 0.770 |
| miR-100-5p | 0.0015 | 0.0014 | 0.683 |
| miR-103a-3p | 1.282 | 1.177 | 0.253 |
| miR-125b-5p | 0.0036 | 0.0031 | 0.367 |
| miR-126-3p | 0.227 | 0.210 | 0.620 |
| miR-130b-3p | 0.480 | 0.456 | 0.359 |
| miR-133a-3p | 0.107 | 0.089 | 0.235 |
| miR-143-3p | 0.025 | 0.020 | 0.231 |
| miR-145-5p | 0.094 | 0.089 | 0.531 |
| miR-146a-5p | 1.024 | 0.939 | 0.442 |
| miR-155-5p | 0.963 | 0.921 | 0.815 |
| miR-181a-5p | 0.216 | 0.202 | 0.193 |
| miR-195-5p | 0.079 | 0.067 | 0.875 |
| miR-199a-5p | 0.046 | 0.040 | 0.370 |
| miR-210-3p | 0.092 | 0.091 | 0.886 |
| miR-221-3p | 0.514 | 0.458 | 0.219 |
| miR-342-3p | 2.500 | 2.485 | 0.873 |
| miR-499a-5p | 0.212 | 0.164 | 0.356 |
| miR-574-3p | 0.137 | 0.125 | 0.331 |
